# Alpha protein kinase 3 gene therapy restores heart function in mouse and human models of cardiomyopathy

**DOI:** 10.1038/s44161-026-00843-1

**Published:** 2026-08-19

**Authors:** James W. McNamara, Ellen B. Keen, Rebecca Sutton, Yinghan She, Hannah Huckstep, Lina H. H. Le, Neda R. Mehdiabadi, Madhavan S. Venkatesh, Brendan Griffen, Richard J. Mills, Christina V. Theodoris, James E. Hudson, Drew M. Titmarsh, Sean J. Humphrey, Enzo R. Porrello, David A. Elliott

**Affiliations:** 1 Murdoch Children’s Research Institute, Royal Children’s Hospital, Melbourne, Victoria Australia; 2 Melbourne Centre for Cardiovascular Genomics and Regenerative Medicine, Royal Children’s Hospital, Melbourne, Victoria Australia; 3 Department of Anatomy and Physiology, University of Melbourne, Melbourne, Victoria Australia; 4 Novo Nordisk Foundation Centre for Stem Cell Medicine (reNEW), Murdoch Children’s Research Institute, Melbourne, Victoria Australia; 5 Australian Regenerative Medicine Institute, Monash University, Melbourne, Victoria Australia; 6 Gladstone Institute of Cardiovascular Disease, San Francisco, CA USA; 7 Gladstone Institute of Data Science and Biotechnology, San Francisco, CA USA; 8 Department of Computational and Systems Biology, University of California, Los Angeles, CA USA; 9 Dynomics Inc, San Francisco, CA USA; 10 Dynomics Pty Ltd, Brisbane, Queensland Australia; 11 Department of Paediatrics, University of Melbourne, Melbourne, Victoria Australia; 12 Department of Pediatrics, Institute for Human Genetics, Cardiovascular Research Institute, University of California, Los Angeles, San Francisco, CA USA; 13 School of Biomedical Sciences, The University of Queensland, Brisbane, Queensland Australia; 14 QIMR Berghofer, Brisbane, Queensland Australia; 15 Present Address: Victor Chang Cardiac Research Institute, Darlinghurst, New South Wales Australia

**Keywords:** Cardiac hypertrophy, Gene therapy, Pluripotent stem cells, Molecular medicine

## Abstract

Truncating variants in the *ALPK3* gene (encoding alpha protein kinase 3) cause severe cardiomyopathy for which no curative treatment exists^1–3^. Here we establish an adeno-associated virus (AAV)-mediated gene replacement therapy to deliver full-length human *ALPK3*. AAV-*ALPK3* prevented disease in neonatal *Alpk3-*mutant mice and reversed established pathology in adults, with proteomic analysis demonstrating reversal of more than 95% of the molecular disease signature. Beyond ALPK3 deficiency, we explored broader therapeutic potential based on ALPK3’s regulatory role in proteostasis, a pathway commonly disrupted across cardiomyopathies. ALPK3 expression is reduced in cardiomyocytes with *TTN*-truncating variants, the most prevalent cause of dilated cardiomyopathy, and the encoded titin protein has a protein quality control network in common with ALPK3. AAV-*ALPK3* restored contractile function in human cardiac organoids with an *ALPK3*- or *TTN*-truncating variant. These findings provide proof of concept for *ALPK3* gene therapy in patients with *ALPK3* cardiomyopathy and reveal potential for indication expansion to cardiomyopathies associated with *TTN*-truncating variants, which are not amenable to gene replacement therapy due to size limitations.

## Abstract (web summary)

McNamara et al. demonstrate that adeno-associated virus-mediated delivery of full-length human *ALPK3* prevents cardiomyopathy in neonatal mouse models and reverses established disease in adult mice, restoring cardiac structure, function and proteostasis, as well as contractile function in human cardiac organoids carrying *ALPK3*- or titin-truncating variants.

## Main

Advances in our understanding of cardiomyopathy genetics have led to an emerging wave of precision therapies targeting the cause of disease rather than management of secondary symptoms^4,5^. The success of the myosin inhibitor mavacamten provides a prominent example of targeting the underlying mechanisms driving disease to improve clinical outcomes for patients with cardiomyopathy^4^. Several gene replacement therapies are also showing promise in clinical trials for some of the most common inherited forms of hypertrophic and arrhythmogenic cardiomyopathy caused by mutations in *MYBPC3* (refs. ^6,7^) and *PKP2* (refs. ^8,9^), as well as for nonischemic and ischemic heart failure via cardiotropic delivery of constitutively active protein phosphatase 1 inhibitor 1 (ref. ^10^) and YAP^11^, respectively. Therefore, expansion of gene therapies to other inherited cardiomyopathies may represent an opportunity to provide life-changing treatment for patients.

Loss-of-function mutations in the *ALPK3* gene encoding myogenic alpha protein kinase 3 have emerged as an important genetic cause of cardiomyopathy, accounting for around 2% of hypertrophic cardiomyopathy cases—a target population comparable in size to populations with Fabry disease or Friedreich ataxia that have established gene therapy targets^1,3^. Biallelic variants cause a severe, and often lethal, form of neonatal cardiomyopathy^1,3^, while autosomal dominant variants are strongly linked to adult-onset hypertrophic (and to a lesser extent dilated and arrhythmogenic) cardiomyopathy^2,12^. Importantly, ALPK3 orchestrates a protein quality control hub at the sarcomere to maintain turnover of contractile proteins^13–15^. Given the loss-of-function nature of *ALPK3* variants, we hypothesized that a gene replacement therapy could provide functional benefit for *ALPK3* cardiomyopathy.

**Fig. 1:**
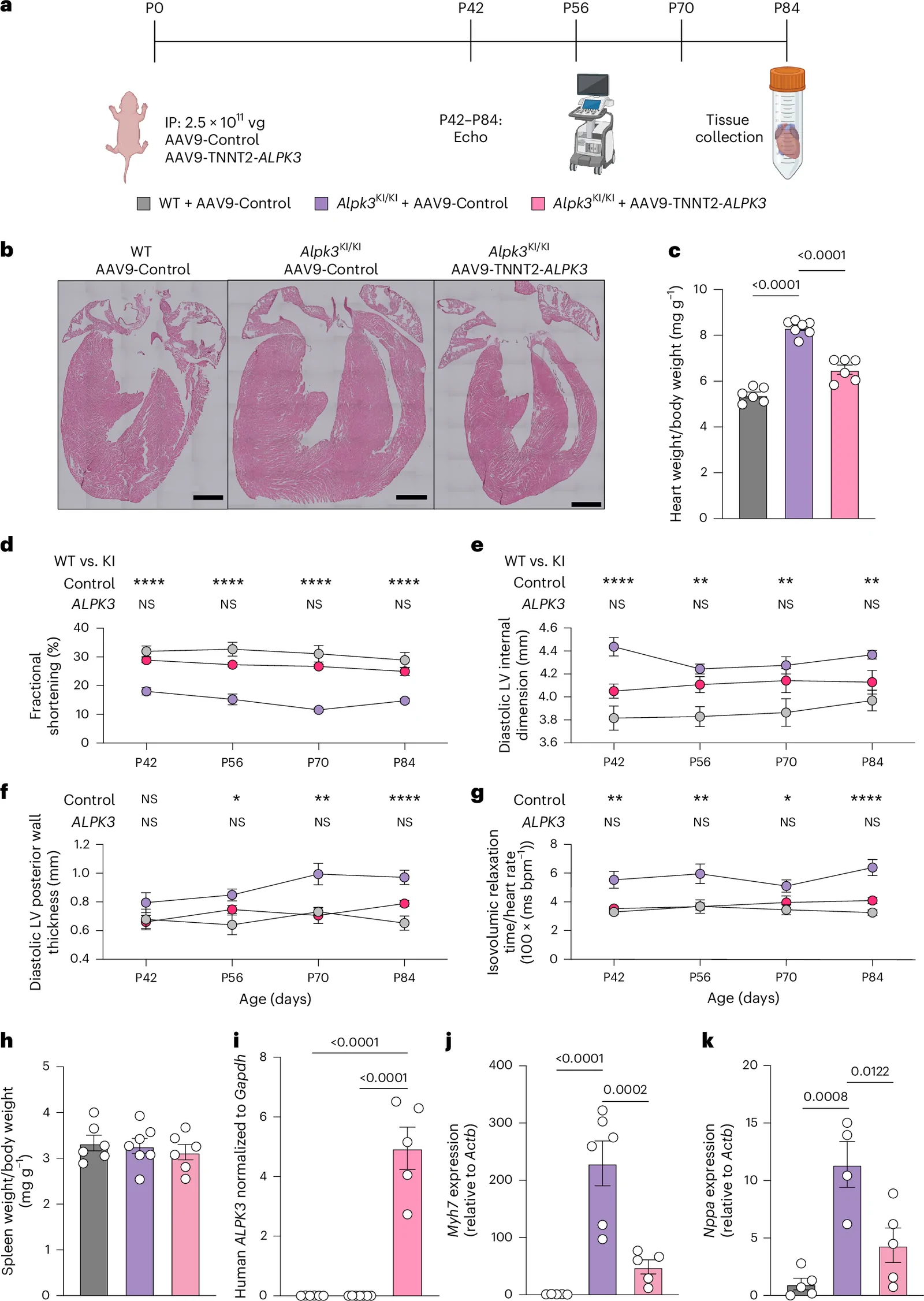
Neonatal delivery of AAV9-TNNT2-*ALPK3* prevents cardiomyopathy in *Alpk3*^*W1538X*^ mice. **a**, Experimental schematic for testing AAV9-TNNT2-*ALPK3* in CRISPR knock-in (*Alpk3*^KI/KI^) mice carrying the patient-derived *Alpk3*^*W1538X*^ variant. Echo, echocardiography; IP, intravenous; vg, viral genomes. **b**, Representative hematoxylin and eosin-stained heart sections (scale bars, 1 mm). **c**, Heart weight to body weight ratios for WT and *Alpk3*^*W1538X*^ mice at the completion of the study (WT, *n* = 6; *Alpk3*^*W1538X*^ AAV9-Control, *n* = 7; *Alpk3*^*W1538X*^ AAV9-TNNT2-*ALPK3*, *n* = 6; one-way ANOVA with Tukey’s multiple comparisons test). **d**–**g**, Select echocardiography measurements, including fractional shortening (**d**), diastolic left ventricular (LV) internal dimension (**e**), diastolic posterior wall thickness (**f**) and heart rate-normalized isovolumic relaxation time (**g**) across the timescale of the experiment (WT, *n* = 6; *Alpk3*^*W1538X*^ AAV9-Control, *n* = 7; *Alpk3*^*W1538X*^ AAV9-TNNT2-*ALPK3*, *n* = 6; two-way ANOVA with Tukey’s multiple comparisons test). bpm, beats per minute. **h**, Spleen weight as a surrogate measure of organ toxicity (WT, *n* = 6; *Alpk3*^*W1538X*^ AAV9-Control, *n* = 7; *Alpk3*^*W1538X*^ AAV9-TNNT2-*ALPK3*, *n* = 6). **i**, Human *ALPK3* gene expression from left ventricular tissue (WT, *n* = 6; *Alpk3*^*W1538X*^ AAV9-Control, *n* = 7; *Alpk3*^*W1538X*^ AAV9-TNNT2-*ALPK3*, *n* = 5; one-way ANOVA with Tukey’s multiple comparisons test). **j**,**k**, Expression levels of cardiac hypertrophy marker *Myh7* (WT, *n* = 5; *Alpk3*^*W1538X*^ AAV9-Control, *n* = 6; *Alpk3*^*W1538X*^ AAV9-TNNT2-*ALPK3*, *n* = 5; one-way ANOVA with Tukey’s multiple comparisons test) (**j**) and *Nppa* (WT, *n* = 5; *Alpk3*^*W1538X*^ AAV9-Control, *n* = 4; *Alpk3*^*W1538X*^ AAV9-TNNT2-*ALPK3*, *n* = 5; one-way ANOVA with Tukey’s multiple comparisons test) (**k**). *P < 0.05; **P < 0.01; ****P < 0.0001; NS, not significant. Data are presented as mean ± s.e.m. Panel **a** created in BioRender; Mcnamara, J. https://biorender.com/ethxb94 (2026). Source data

**Extended Data Fig. 1:**
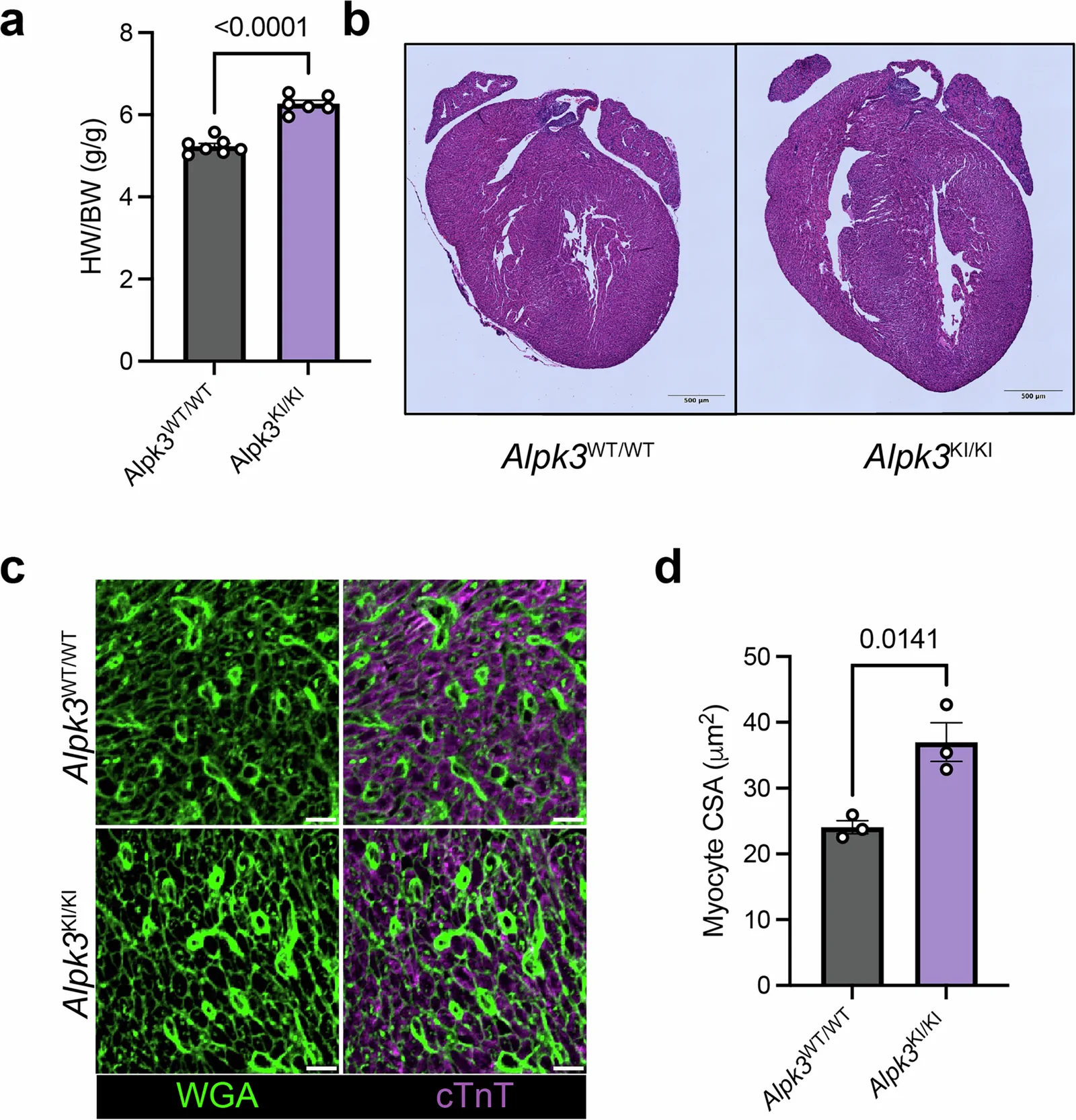
Neonatal onset of cardiomyopathy in *Alpk3*^W1538X^ mice. **a**, Heart to body weight ratio of P0 WT and *Alpk3*^KI/KI^ mice (WT, n = 7; *Alpk3*^KI/KI^, n = 6; two-tailed unpaired t-test). **b**, Representative H&E sections of P0 WT and *Alpk3*^KI/KI^ mice. **c**, wheat germ agglutinin (green) and cardiac troponin T (purple) stained P0 WT and *Alpk3*^KI/KI^ hearts (scale bar = 10 µm). **d**, Quantification of myocyte cross sectional area (n = 3 per group; ~100 myocytes measured per mouse; two-tailed unpaired t-test). Data is presented as mean ± s.e.m. Source data

**Extended Data Fig. 2:**
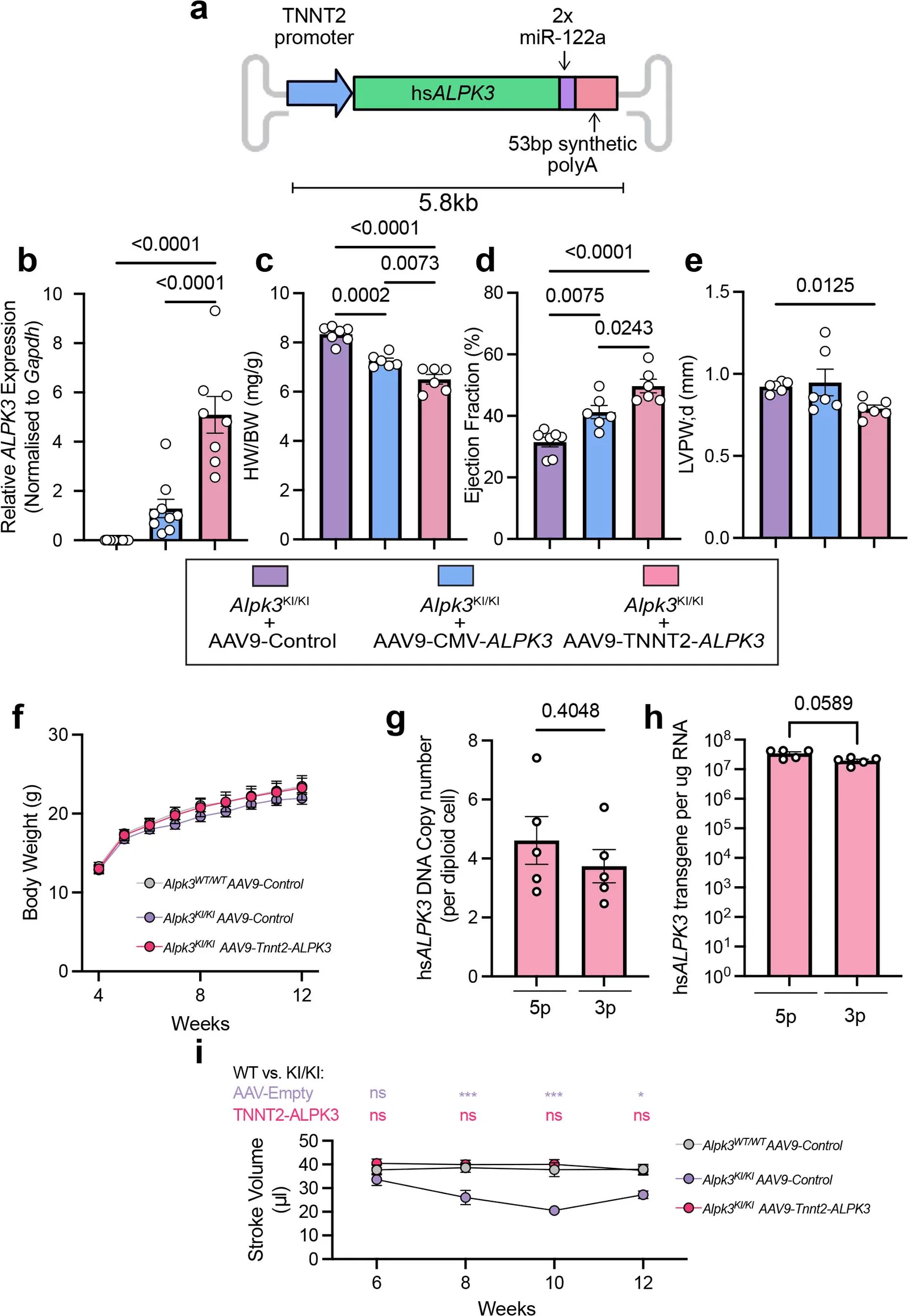
Characterization of neonatal protection study. **a**, Schematic of the AAV TNNT2-*ALPK3* vector. **b**, qRT-PCR analysis of *ALPK3* gene expression between AAV9-Control, AAV9-CMV-*ALPK3*, and AAV9-TNNT2*-ALPK3* hearts (AAV9-Control, n = 14; AAV9-CMV-ALPK3, n = 9; AAV9-TNNT2-ALPK3, n = 8; n represents individual mice with data from WT and *Alpk3*^KI/KI^ mice combined; using a one-way ANOVA with Tukey’s multiple comparisons test. **c**, Heart to body weight ratio, **d**, Ejection Fraction, and **e**, diastolic left ventricular posterior wall thickness of *Alpk3*^KI/KI^ mice treated at P0 with AAV9-Control, AAV9-CMV-*ALPK3*, AAV9-TNNT2-*ALPK3*. **f**, body weight measurements over course of experiment (*ALPK3*^W1538X^ AAV9-Control, n = 7; *ALPK3*^W1538X^ AAV9-CMV-ALPK3, n = 6; *ALPK3*^W1538X^ AAV9-TNNT2-*ALPK3*, n = 6; using a one-way ANOVA with Tukey’s multiple comparisons test). **g**, qPCR measurement of hs*ALPK3* vector DNA copy number per diploid cell at 5’ and 3’ regions of gene (n = 5 per group; n represents DNA preparations from independent mice; two-tailed unpaired t-test). **h**, qRT-PCR measurement of hs*ALPK3* transgene copy number per µg RNA at 5’ and 3’ regions of gene (n = 5 per group; n represents RNA preparations from independent mice; two-tailed unpaired t-test). **i**, Stroke volume from echocardiography (WT, n = 6; *ALPK3*^W1538X^ AAV9-Control, n = 7; *ALPK3*^W1538X^ AAV9-*ALPK3*, n = 6; using a two-way ANOVA with Tukey’s multiple comparisons test; *p < 0.05, ***p < 0.001). Data are the mean ± s.e.m. Source data

**Extended Data Fig. 3:**
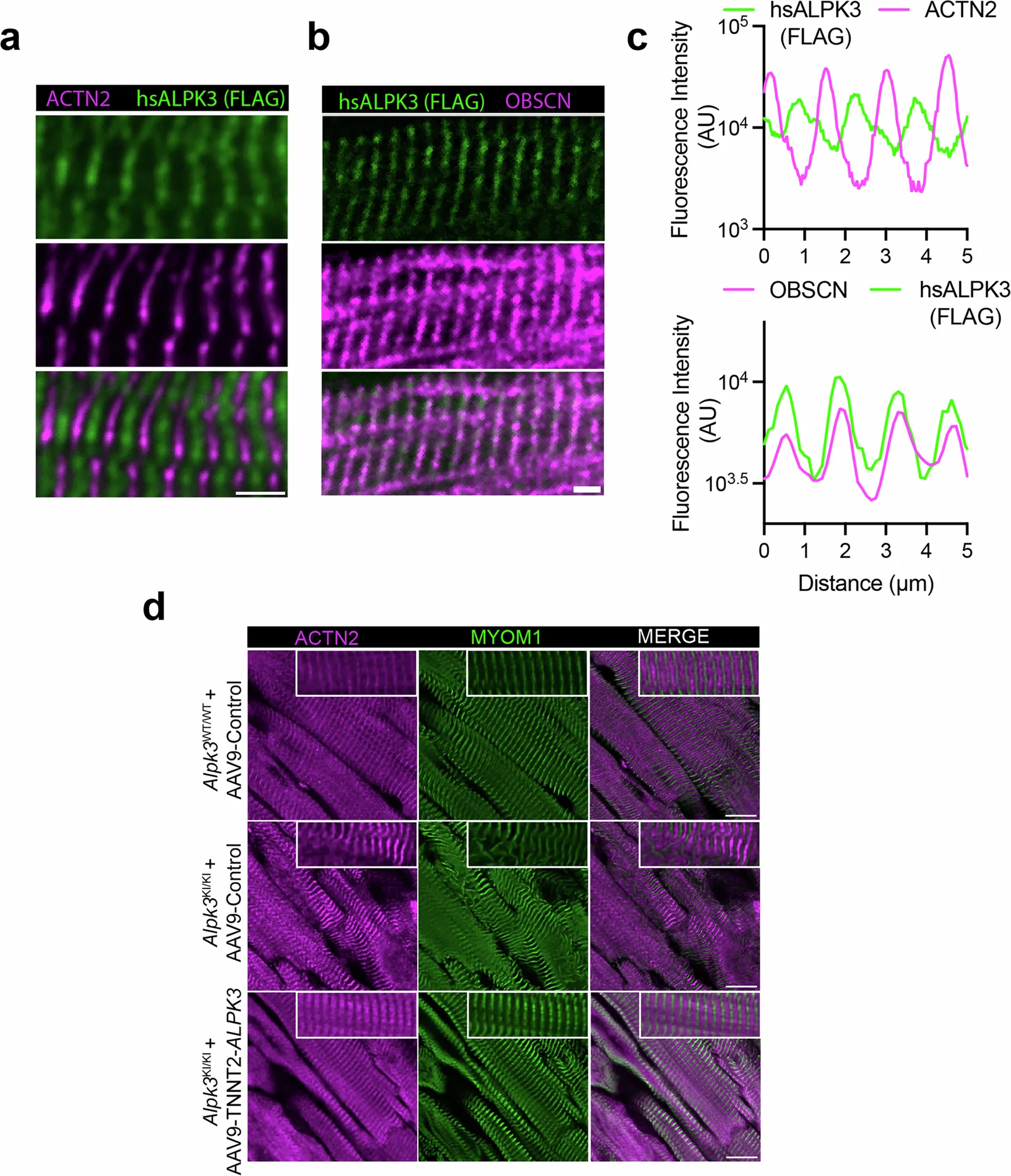
Histological assessment of neonatal protection study. **a**, Staining of mouse hearts treated with AAV9-CMV-*ALPK3-DDK* with anti-flag antibody (green) and ACTN2 (magenta) shows interdigitation (scale bar = 5 µm). **b**, Staining of mouse hearts treated with AAV9-CMV-*ALPK3-DDK* with anti-flag antibody (green) and MYOM1 (magenta) shows interdigitation (scale bar = 10 µm). **c**, Representative line scans of **a** and **b**. **d**, Staining of heart sections with ACTN2 (magenta, z-disk) and MYOM1 (green, m-band), scale bar= 10 µm.

We first tested the efficacy of adeno-associated virus carrying *ALPK3* (AAV9-*ALPK3*) in an in vivo mouse model of *ALPK3* cardiomyopathy (Fig. 1a). We used *Alpk3*^*W1538X*^ mice, which model a severe *ALPK3*-truncating variant, *ALPK3*^*W1765X*^, found in patients^1^. Homozygous mice are born with enlarged hearts (Extended Data Fig. 1a–d), phenocopying the severity and early-onset nature of disease in humans with this variant^1,13^. Pilot studies demonstrated fivefold-stronger expression of human *ALPK3* using the cardiotropic *TNNT2* promoter (Extended Data Fig. 2a) compared with the ubiquitous CMV promoter (Fig. 1i and Extended Data Fig. 2b–e). This likely results from the optimization of this therapeutic vector relative to the construct with a CMV promoter, including replacement of the bovine growth hormone polyadenylation sequence with a 53-bp synthetic polyadenylation signal sequence and removal of the nonregulatory spacer sequence, ultimately resulting in a 300-nucleotide reduction in vector genome size. Importantly, the transgene-encoded Flag-tagged ALPK3 protein correctly localized to the M band of the sarcomere, as evidenced by interdigitation with ACTN2 (Extended Data Fig. 3a,c) and colocalization with OBSCN (Extended Data Fig. 3b,c). Based on these findings, AAV9 with *ALPK3* under the control of the cardiomyocyte-specific *TNNT2* promoter (AAV9-TNNT2-*ALPK3*) was used for all subsequent in vivo experiments. In these experiments, we delivered 2.5 × 10^11^ viral genomes of this construct via intraperitoneal injection at postnatal day 0 (P0) (Extended Data Fig. 2a), which had no impact on body mass (Extended Data Fig. 2f). This AAV9-TNNT2-*ALPK3* vector (5.8 kb) exceeds the conventional 4.7-kb packaging capacity of the AAV genome. However, quantitative PCR (qPCR) showed no significant difference in the number of DNA copies for the two ends of the transgene, suggesting equivalent delivery of both ends of the viral genome (Extended Data Fig. 2g). Similarly, no significant difference was found in the number of transgene RNA copies for the 5ʹ and 3ʹ ends of the sequence (Extended Data Fig. 2h), consistent with the established biology of *ALPK3-*truncating variants that suggests that 3ʹ-truncated *ALPK3* mRNA undergoes nonsense-mediated decay^3^. Thus, AAV9-TNNT2-*ALPK3* efficiently delivers full-length *ALPK3* mRNA within cardiac tissue.

**Extended Data Fig. 4:**
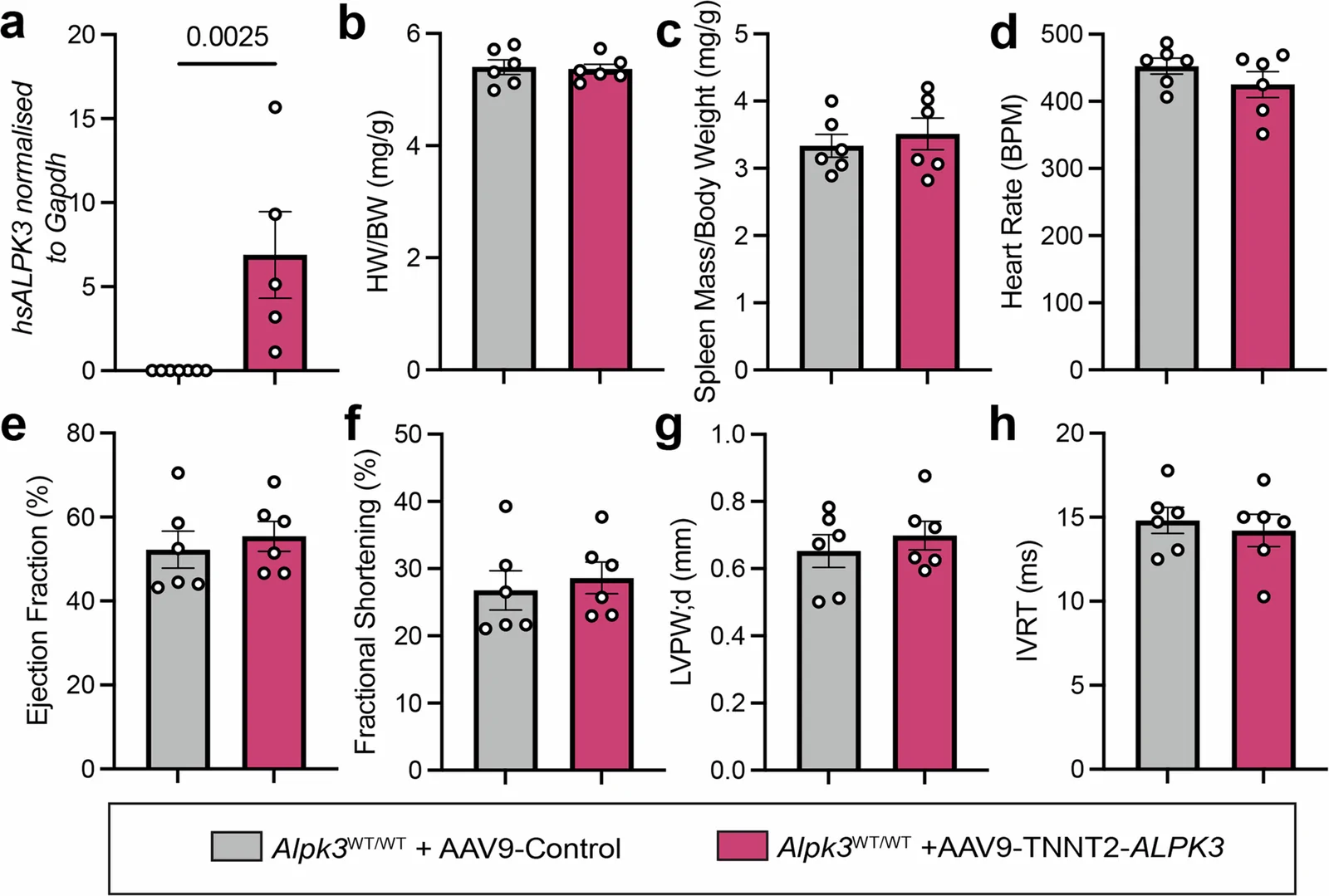
Negligible impact of AAV9-TNNT2-*ALPK3* in wild-type mice. **a**, Over-expression of *ALPK3* in wild-type mice measured by qPCR (WT AAV9-Control, n = 7; WT AAV9-ALPK3, n = 5; two-tailed non-parametric Mann-Whitney t-test). **b**, Heart to body weight ratio **c**, spleen to body weight ratio (n = 6 per group). Select echocardiography data: **d**, heart rate; **e**, ejection fraction; **f**, fractional shortening; **g**, diastolic left ventricular posterior wall thickness, and **h**, isovolumic relaxation time (n = 6 per group; two-tailed Welch’s unpaired t-test was performed.). Data are the mean ± s.e.m. Source data

Histological comparison of wild-type (WT) and *Alpk3*^*W1538X*^ mice demonstrated that treatment with AAV9-TNNT2-*ALPK3* reversed the morphological abnormalities of the heart in *Alpk3*^*W1538X*^ mice (Fig. 1b). By 12 weeks of age, treatment with AAV9-TNNT2-*ALPK3* significantly lowered heart mass in *Alpk3*^*W1538X*^ mice toward that in WT controls, reducing it by 63% (Fig. 1c). Biweekly echocardiography (performed blinded to treatment) revealed near-complete restoration of almost all functional and structural parameters (Fig. 1d–g and Supplementary Table 1), including fractional shortening (P84: 31% ± 4% for *Alpk3*^*W1538X*^ AAV9-Control versus 50% ± 5% for *Alpk3*^*W1538X*^ AAV9*-*TNNT2-*ALPK3*; *P* < 0.0001), stroke volume (P84: 27 ± 4 µl versus 37 ± 5 µl; *P* = 0.0017; Extended Data Fig. 2i), wall thickness (diastolic posterior wall thickness at P84: 0.97 ± 0.13 mm versus 0.79 ± 0.05 mm; *P* = 0.0341 at P84) and diastolic function (isovolumic relaxation time at P84: 32 ± 7 ms versus 19 ± 2 ms; *P* < 0.0001). As previously described^13^, sarcomeric structure in *Alpk3*^*W1538X*^ AAV9-Control mice was compromised, as demonstrated by disorganization of ACTN2 and MYOM1 staining (Extended Data Fig. 3d). Critically, sarcomeric structure appeared similar to that of WT hearts in *Alpk3*^*W1538X*^ mice treated with AAV9*-*TNNT2-*ALPK3*. We observed no gross signs of off-target organ pathologies indicated by spleen mass (Fig. 1h). Treatment with AAV9*-*TNNT2-*ALPK3* also significantly reduced the expression of cardiac hypertrophy markers *Myh7* and *Nppa* (Fig. 1j,k). No impact on cardiac function was observed in WT mice treated with AAV9*-*TNNT2-*ALPK3* compared with AAV9-Control (Extended Data Fig. 4a–h), demonstrating that ALPK3 overexpression has no negative impact on cardiac function. These results demonstrate that neonatal administration of a viral construct with cardiotropic expression of full-length *ALPK3* effectively prevents cardiac dysfunction and progression of *ALPK3* cardiomyopathy in vivo.

**Fig. 2:**
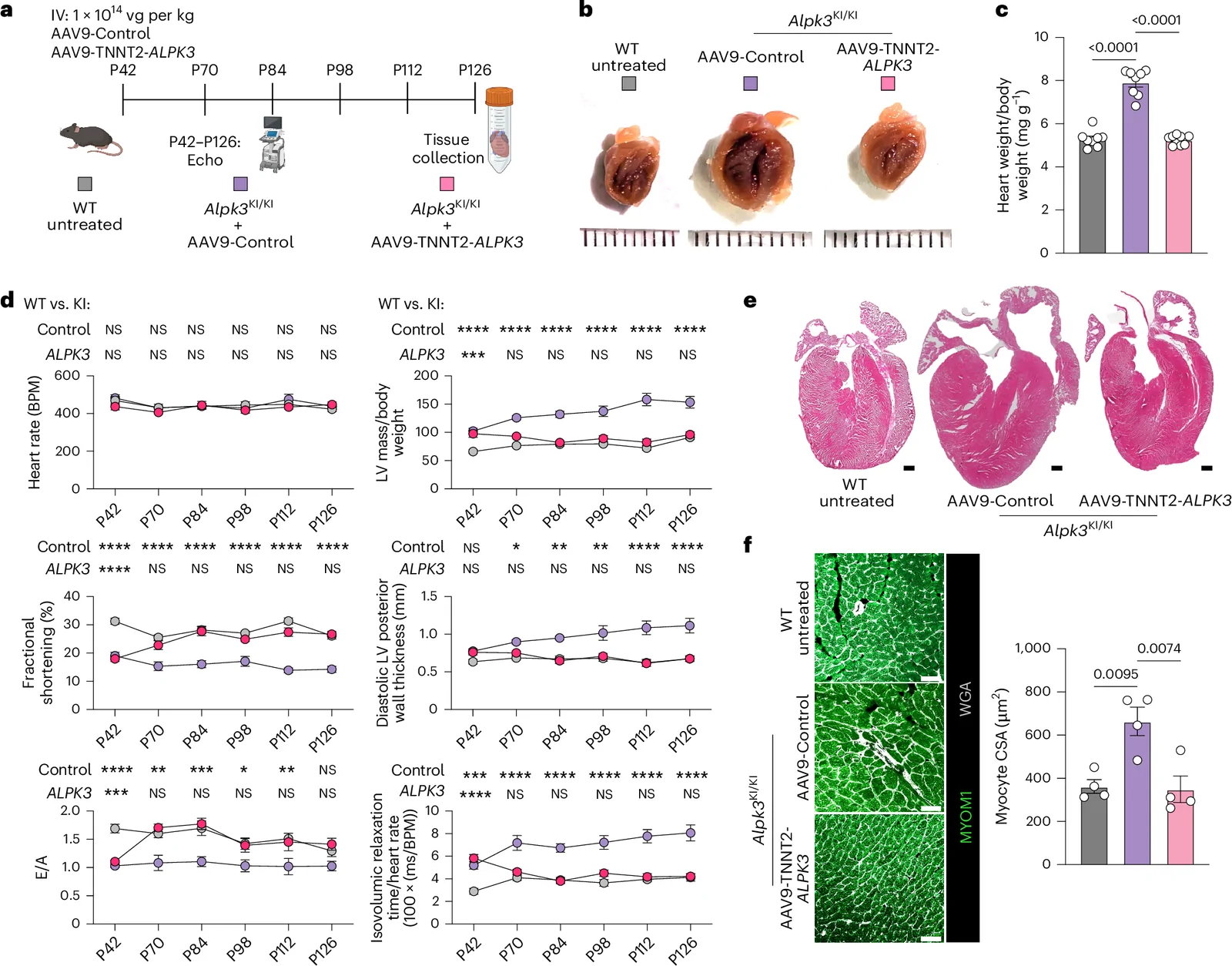
Adult delivery of AAV-TNN2-*ALPK3* reverts established cardiac pathophysiology in *Alpk3*^*W1538X*^ mice. **a**, Schematic of the experiment testing AAV9-TNNT2-*ALPK3* in *Alpk3*^*W1538X*^ knock-in mice carrying the patient-derived *Alpk3*^*W1538X*^ variant. **b**, Representative gross heart morphology at P126 (scale divisions, 1 mm). **c**, Heart weight to body weight measure at P126 (WT, *n* = 8; *Alpk3*^*W1538X*^ AAV9-Control, *n* = 8; *Alpk3*^*W1538X*^ AAV9-*ALPK3*, *n* = 9; one-way ANOVA with Tukey’s multiple comparisons test). **d**, Select echocardiography measurements of heart rate, corrected left ventricular mass/body weight, fractional shortening, diastolic left ventricular posterior wall thickness, E/A ratio and heart rate-normalized isovolumic relaxation time (WT, *n* = 8; *Alpk3*^*W1538X*^ AAV9-Control, *n* = 8; *Alpk3*^*W1538X*^ AAV9-TNNT2-*ALPK3*, *n* = 9; two-way ANOVA with Tukey’s multiple comparisons test). **e**, Representative hematoxylin and eosin-stained heart sections (scale bars, 500 µm). **f**, Wheat germ agglutinin (WGA; gray) and MYOM1 (myomesin-1; green) staining of heart sections and associated quantification (*n* = 4 per group (average of 100 myocytes per animal from three images); one-way ANOVA with Tukey’s multiple comparisons test). Scale bars, 20 µm. *P < 0.05; **P < 0.01; ***P < 0.001; ****P < 0.0001. Data are presented as mean ± s.e.m. Panel **a** created in BioRender; McNamara, J. https://biorender.com/yo8rsl9 (2026). Source data

**Fig. 3:**
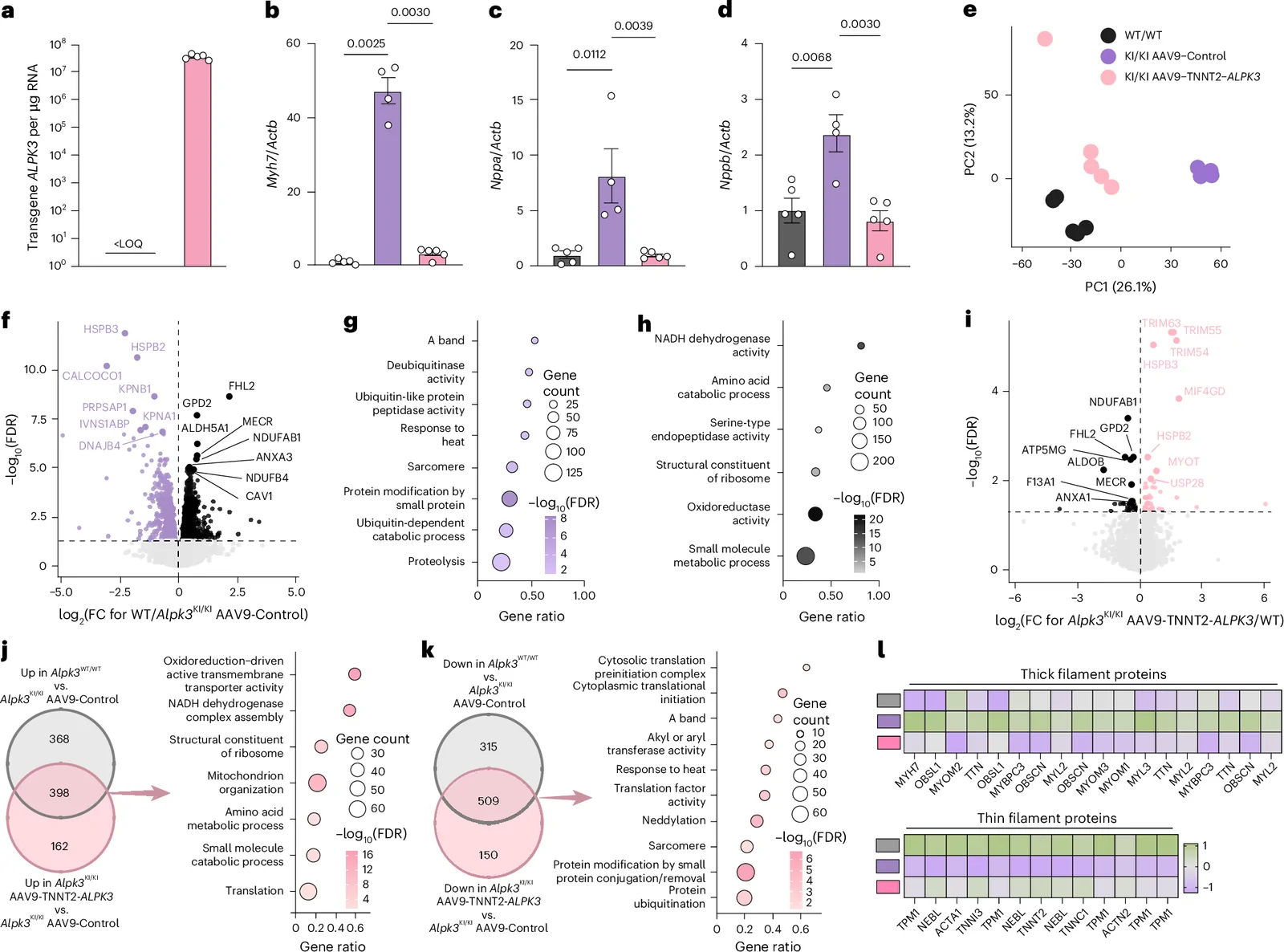
Molecular analysis reveals reversal of proteostasis dysregulation following treatment with AAV9-TNNT2-*ALPK3*. **a**, qPCR analysis of *ALPK3* transgene mRNA copies per µg of RNA (*n* = 5 per group). LOQ, limit of quantification. **b**–**d**, Expression levels of cardiac hypertrophy markers *Myh7* (**b**), *Nppa* (**c**) and *Nppb* (**d**) (WT, *n* = 5; *Alpk3*^*W1538X*^ AAV9-Control, *n* = 4; *Alpk3*^*W1538X*^ AAV9-TNNT2-*ALPK3*, *n* = 5; *n* represents individual mice; one-way ANOVA with Tukey’s multiple comparisons test). **e**, Principal-component analysis of proteomics samples from the adult treatment groups. **f**, Volcano plot of differentially expressed proteins between WT and *Alpk3*^*W1538X*^ AAV9-Control mice. FC, fold change. **g**,**h**, GO terms of proteins enriched (**g**) or suppressed (**h**) in *Alpk3*^*W1538X*^ AAV9-Control versus WT mice. **i**, Volcano plot of differentially expressed proteins between WT and *Alpk3*^*W1538X*^ AAV9-TNNT2-*ALPK3* mice. **j**, Overlap of proteins enriched in comparisons of WT versus *Alpk3*^*W1538X*^ AAV9-Control mice and *Alpk3*^*W1538X*^ AAV9-TNNT2-*ALPK3* versus *Alpk3*^*W1538X*^ AAV9-Control mice and associated GO terms. **k**. Overlap of proteins suppressed in comparisons of WT versus *Alpk3*^KI/KI^ AAV9-Control and *Alpk3*^KI/KI^ AAV9-TNNT2-*ALPK3* versus *Alpk3*^*W1538X*^ AAV9-Control mice and associated GO terms. **l**, Heatmap of the protein expression of sarcomeric thick and thin filament proteins between groups; color scale represents *z*-score for each protein. *n* = 5 per group for proteomic analyses. Data are presented as mean ± s.e.m. Source data

**Extended Data Fig. 5:**
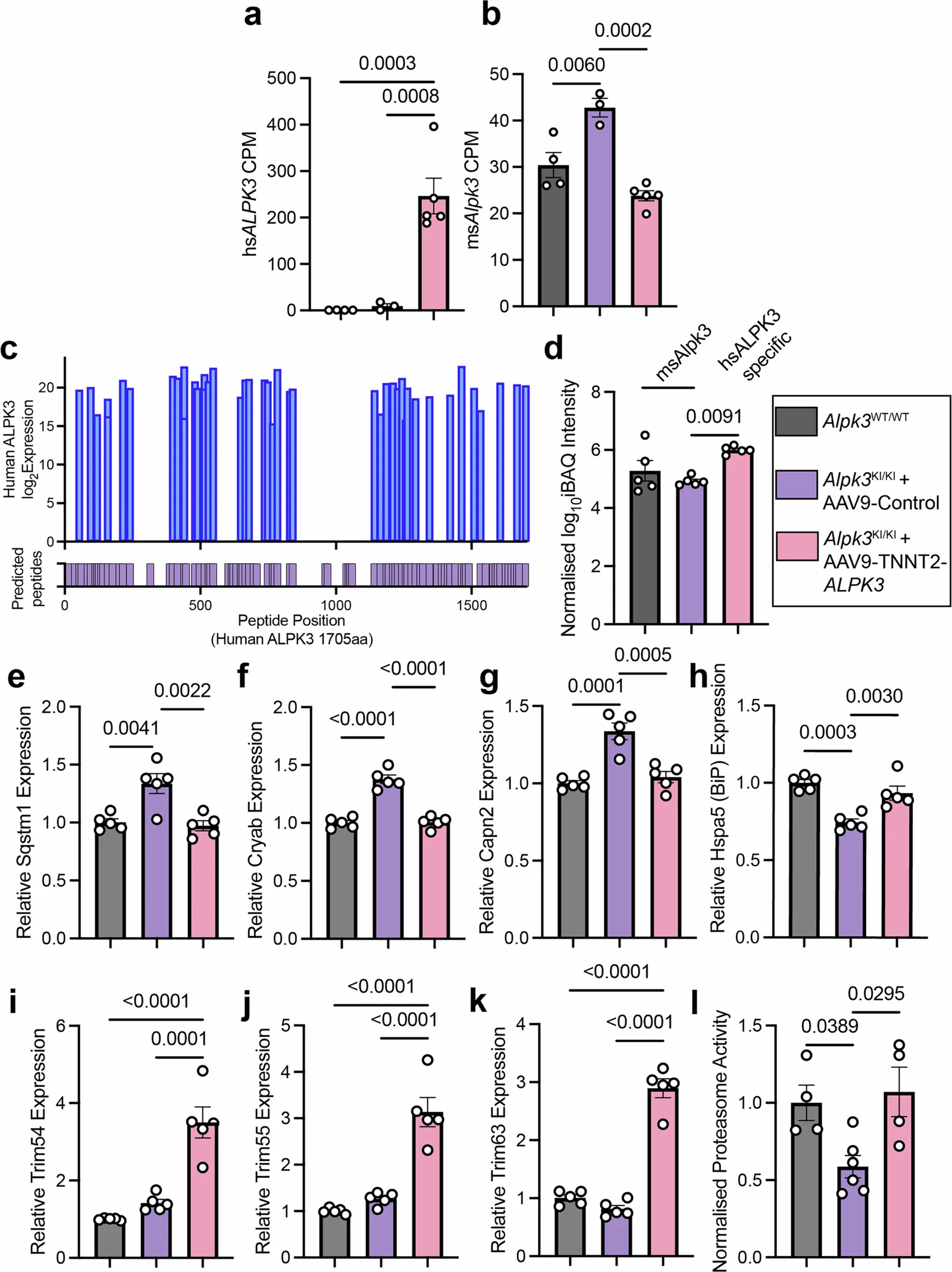
Molecular assessment of ALPK3 gene therapy in adult mice. **a**, RNA sequencing expression (Counts per million, CPM) of hs*ALPK3* and **b**, ms*Alpk3* (WT, n = 4; *Alpk3*^KI/KI^ AAV9-Control, n = 3; *Alpk3*^KI/KI^ AAV9-ALPK3, n = 5; one-way ANOVA with Tukey’s multiple comparison test). **c**, Quantitative expression of human specific peptides detected (in blue) in proteomics analysis of *Alpk3*^KI/KI^ AAV9-TNNT2-*ALPK3* samples relative to predicted tryptic peptides (in purple). **d**, Quantitative comparison (measured by iBAQ intensity) of mouse Alpk3 in WT and *Alpk3*^KI/KI^ AAV9-Control and human-specific peptides in *Alpk3*^KI/KI^ AAV9-TNNT2-*ALPK3* (WT, n = 5; *Alpk3*^KI/KI^ AAV9-Control, n = 5; *Alpk3*^KI/KI^ AAV9-ALPK3, n = 5; one-way ANOVA with Tukey’s multiple comparison test). Representative expression of proteins involved in the proteostasis regulation **e**, Sqstm1; **f**, Cryab; **g**, Capn2, and **h** Hspa5/Bip. Expression of sarcomere-targeted E3 ligases **i**, Trim54/Murf3; **j**, Trim55/Murf2; **k**, Trim63/Murf1 (for e-k (WT, n = 5; *Alpk3*^KI/KI^ AAV9-Control, n = 5; *Alpk3*^KI/KI^ AAV9-ALPK3, n = 5; one-way ANOVA with Tukey’s multiple comparison test). **l**, Quantification of proteasome activity assay in cytosolic mouse hearts (WT, n = 4; *Alpk3*^KI/KI^ AAV9-Control, n = 6; *Alpk3*^KI/KI^ AAV9-ALPK3, n = 4; n represents individual mouse hearts; one-way ANOVA with Tukey’s multiple comparison test).). Data are the mean ± s.e.m. Source data

To determine whether this *ALPK3* gene therapy could restore function in *Alpk3*^*W1538X*^ mice with cardiomyopathy, we treated 6-week-old mice and assessed cardiac function biweekly (blinded to treatment) until 18 weeks (P126) of age (Fig. 2a). Before randomization of *Alpk3*^*W1538X*^ mice at 6 weeks of age, systolic function (fractional shortening: 59% ± 5% for WT versus 36% ± 6% for *Alpk3*^*W1538X*^; *P* < 0.0001), left ventricular mass (66 ± 3 mg versus 100 ± 4 mg; *P* < 0.0001) and diastolic function (13.2 ± 0.6 ms versus 24.9 ± 0.6 ms; *P* < 0.0001) measurements demonstrated that the adult mice had established cardiomyopathy. qPCR analysis detected ~4 vector DNA copies per diploid cell within the heart (Extended Data Fig. 2b), which resulted in ~3.6 × 10^7^ copies per µg RNA of the *ALPK3* transgene (Fig. 3a and Extended Data Fig. 2h) or ~200 copies per million reads of human-specific *ALPK3* transcripts by RNA sequencing (Extended Data Fig. 5a). Interestingly, *ALPK3* gene therapy restored the expression of mouse *Alpk3* to WT levels, suggesting regulatory feedback (Extended Data Fig. 5b). AAV9-TNNT2-*ALPK3* clearly restored myocardial structure compared with AAV9-Control in *Alpk3*^*W1538X*^ mice (Fig. 2b,e) and completely abrogated the increased heart mass (Fig. 2c). AAV9-TNNT2-*ALPK3* delivery in 6-week-old mice was sufficient to fully rescue systolic function back to WT levels at all timepoints after treatment (Fig. 2d). Unexpectedly, AAV9*-*TNNT2-*ALPK3* also reverted established hypertrophy to WT levels as early as 4 weeks after treatment, as evidenced by the regression of both left ventricular mass and left ventricular wall thickness, and completely restored diastolic function (Fig. 2d; see Supplementary Table 2 for full echocardiographic data). Improved heart function was accompanied by restoration of myocardial structure as assessed by hematoxylin and eosin staining (Fig. 2e) and reversal of cellular hypertrophy as assessed by wheat germ agglutinin staining (Fig. 2f). As hypertrophy is already established at birth (Extended Data Fig. 1c,d), these findings indicate true reversal of cellular and organ-level pathology rather than a protective effect preventing disease progression.

**Extended Data Fig. 6:**
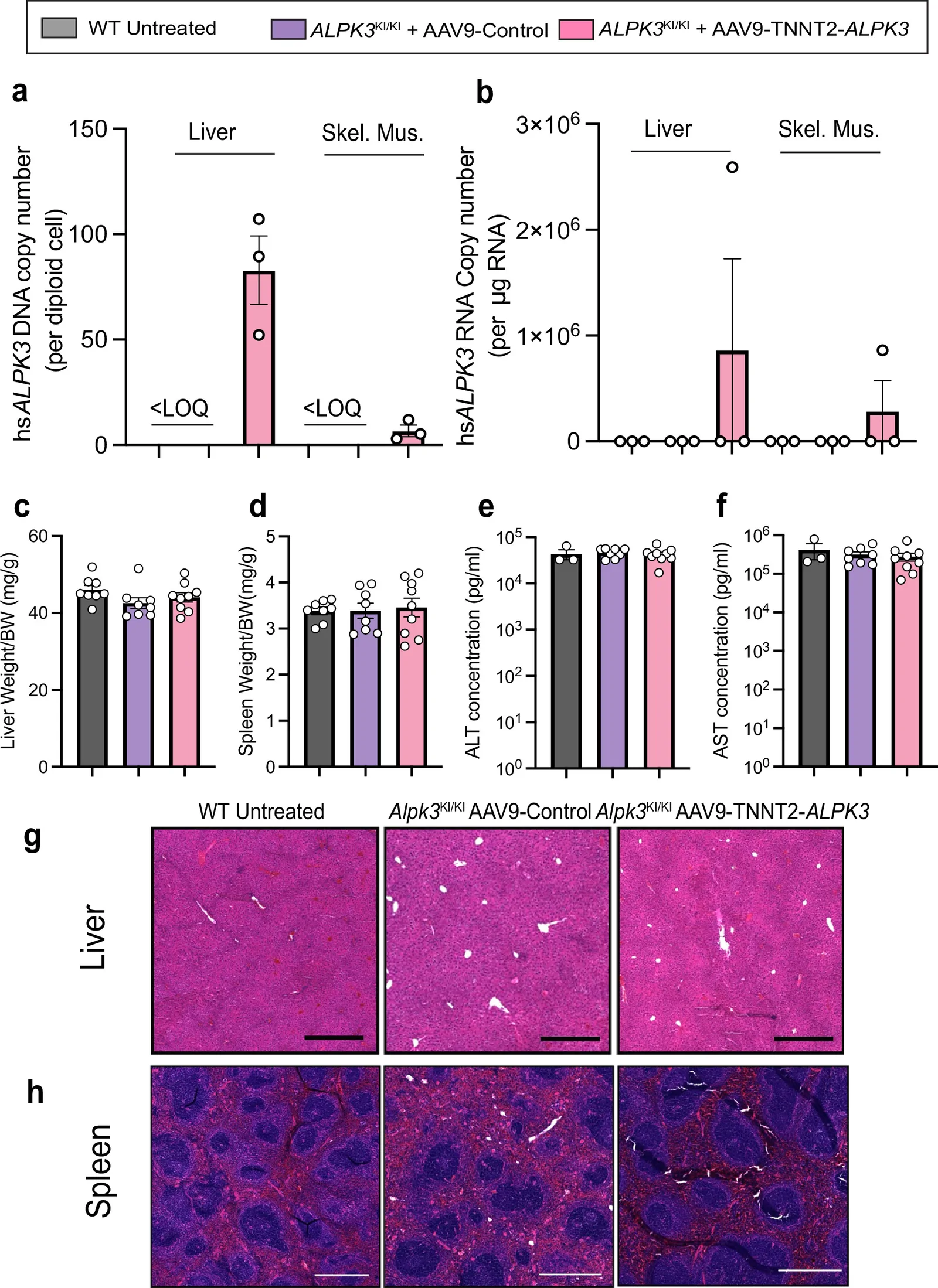
Absence of off-target organ toxicity in adult *Alpk3*^KI/KI^ mice treated with ALPK3 gene therapy. **a**, qPCR measurement of hs*ALPK3* vector DNA copy number per diploid cell in liver and tibialis anterior skeletal muscle (n = 3 DNA preparations per group from independent mice). **b**, qRT-PCR measurement of hs*ALPK3* transgene copy number per µg RNA in liver and skeletal muscle (n = 3 RNA preparations per group from independent mice). **c**, liver and **d**, spleen to body weight ratios (WT, n = 8; *ALPK3*^W1538X^ AAV9-Control, n = 8; *ALPK3*^W1538X^ AAV9-*ALPK3*, n = 9). **e**. Serum alanine aminotransferase (ALT) and **f**, aspartate aminotransferase (AST) levels measured via ELISA (WT, n = 3; *ALPK3*^W1538X^ AAV9-Control, n = 8; *ALPK3*^W1538X^ AAV9-*ALPK3*, n = 9). **g**, Representative H&E stained livers and **h**, spleens across all groups (scale bar = 500 µm). Data are the mean ± s.e.m. Source data

Biodistribution studies in the liver and skeletal muscle showed that, despite robust vector DNA detection (Extended Data Fig. 6a), *ALPK3* transgene mRNA was detected in only one of three samples and at levels an order of magnitude lower than those in cardiac tissue (Extended Data Fig. 6b and Fig. 3a). Thus, our vector design including a cardiac-specific *TNNT2* promoter and a liver-specific silencing miR-122a sequence effectively limits off-target transgene expression (Extended Data Fig. 2a). No evidence of liver or spleen toxicity was observed based on organ mass (Extended Data Fig. 6c,d), serum alanine aminotransferase and aspartate aminotransferase levels (Extended Data Fig. 6e,f) or histological analyses (Extended Data Fig. 6g,h). Together, these data demonstrate that AAV-mediated delivery of *ALPK3* can reverse established pathology with minimal off-target toxicity, supporting its therapeutic potential in established cardiomyopathy.

**Extended Data Fig. 7:**
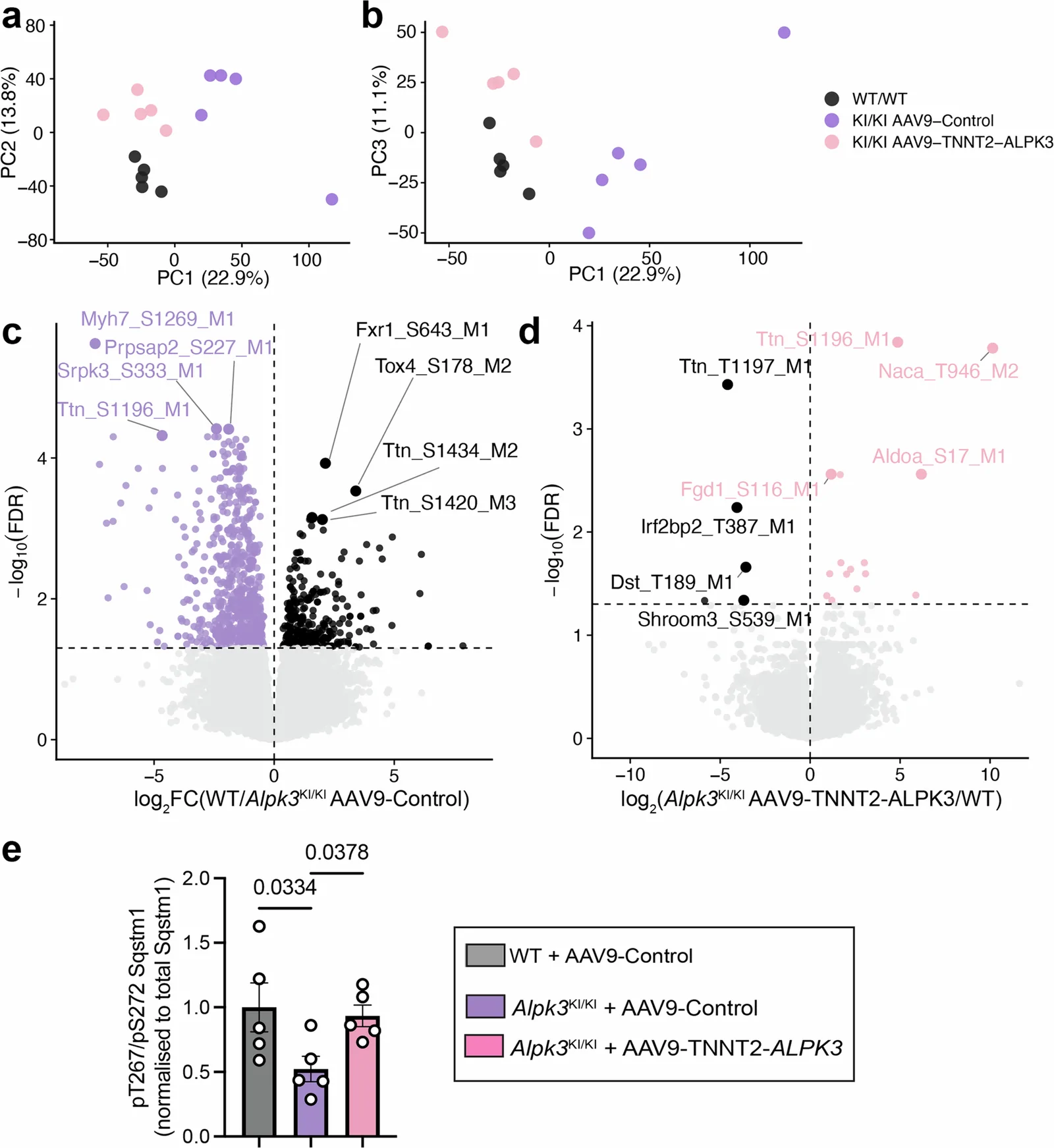
Phosphoproteomic analysis of ALPK3 gene therapy hearts in adult mice. **a**, Principal component analysis (PC2 vs. PC1) across groups. **b**, PCA plots of PC3 vs. PC1 across groups. **c**, Volcano plot of differentially expressed phosphopeptides between WT and *Alpk3*^KI/KI^ AAV9-Control. **d**, Volcano plot of differentially expressed phosphopeptides between WT and *Alpk3*^KI/KI^ AAV9- AAV9-TNNT2-*ALPK3*. **e**. Quantitative analysis pT267/pS272 Sqstm1 normalised to total Sqstm1 (n = 5 per group; One-way ANOVA with Tukey’s multiple comparison test). Data are the mean ± s.e.m. Source data

Previous studies have shown that ALPK3 regulates a sarcomeric protein quality control hub to maintain cytoskeletal integrity^13–15^. Therefore, we hypothesized that the therapeutic effects of AAV9-TNNT2-*ALPK3* are mediated through this mechanism. To address this, we performed qPCR together with quantitative proteomics and phosphoproteomics in adult *Alpk3*^*W1538X*^ mice treated with either AAV9-TNNT2*-ALPK3* or AAV9-Control, alongside untreated WT controls. The expression levels of cardiac stress markers (*Myh7*, *Nppa* and *Nppb*) were normalized to WT levels following *ALPK3* gene therapy (Fig. 3b–d). Principal-component analysis revealed a global proteomic shift toward the WT state in treated hearts (Fig. 3e). Importantly, human-specific ALPK3 peptides were detected across the length of the 1,705-residue protein, with similar expression levels at the N and C termini of the protein, suggesting that full-length protein is produced using this approach (Extended Data Fig. 5c). Due to technical artifacts associated with the detection of tryptic peptides by mass spectrometry (MS), some regions were found below the detection limit. These findings are broadly consistent with our nucleic acid-based assessment of viral genome truncations (Extended Data Fig. 2g,h). Comparison of mouse ALPK3 peptides in control groups and human-specific ALPK3 peptides in *Alpk3*^*W1538X*^ AAV9-TNNT2-*ALPK3* mice supported this, with ALPK3 protein from the transgene detected at levels equivalent to or higher than those of endogenous protein levels (Extended Data Fig. 5d). Of the ~6,000 detected proteins, 1,612 proteins were differentially expressed between WT and *Alpk3*^*W1538X*^ AAV9-Control mice (Fig. 3f). Proteins enriched in *Alpk3*^*W1538X*^ AAV9-Control mice were related to the sarcomeric A band, ubiquitin protein modification, heat shock proteins and proteolysis (Fig. 3g), whereas downregulated proteins were related to the mitochondrion, protein translation and amino acid catabolism (Fig. 3h). Strikingly, only 81 proteins remained differentially expressed between WT and *Alpk3*^*W1538X*^ AAV9-TNNT2-*ALPK3* mice, representing a 95% reversal of the proteomic disease signature (Fig. 3i). This effect was even more pronounced in the phosphoproteome, with 98% normalization (Extended Data Fig. 7a–d). Taken together, these data suggest that *ALPK3* gene therapy acts in part by improving proteostasis.

To further confirm that *ALPK3* gene therapy restores cardiac proteostasis, we assessed the overlap of proteins differentially expressed between WT versus *Alpk3*^*W1538X*^ AAV9-Control mice and *Alpk3*^*W1538X*^ AAV9-TNNT2-*ALPK3* versus *Alpk3*^*W1538X*^ AAV9-Control mice. A total of 398 proteins were upregulated in both comparisons, with Gene Ontology (GO) terms related to translation, amino acid metabolism and the mitochondrion (Fig. 3j). This overlap closely mimics the GO terms for proteins repressed in *Alpk3*^*W1538X*^ AAV9-Control mice (Fig. 3h). Similarly, the 509 shared proteins with reduced expression were related to GO terms including heat shock proteins, the sarcomeric A band and protein ubiquitination (Fig. 3k), closely resembling the GO terms for proteins enriched in *Alpk3*^*W1538X*^ AAV9-Control mice (Fig. 3g). The data confirm a normalization of the proteostasis machinery, with pathways spanning the entire proteostatic spectrum from translation and folding to degradation (Extended Data Fig. 5e–h). Deletion of *Alpk3* was previously demonstrated to result in the accumulation of thick filament proteins^15^, which was supported by our proteomics analysis of the more clinically relevant *Alpk3*^*W1538X*^ mouse model. To further interrogate this, we assessed sarcomeric protein expression and also observed accumulation of all thick filament proteins in *Alpk3*^*W1538X*^ AAV9-Control mice compared with WT mice (Fig. 3l). Further, all thin filament proteins showed reduced abundance in *Alpk3*^*W1538X*^ AAV9-Control mice compared with WT mice. This creates a catastrophic imbalance within the contractile machinery of the heart and likely contributes to the severe contractile dysfunction in this model. Strikingly, AAV9-TNNT2-*ALPK3* restored this balance to the WT state, suggesting that ALPK3 directly regulates sarcomeric proteostasis (Fig. 3l). Supporting this finding of sarcomeric proteostasis, the sarcomere-localized E3 ligases TRIM54, TRIM55 and TRIM63 were specifically upregulated following AAV9-TNNT2-*ALPK3* treatment (Extended Data Fig. 5i–k). In addition, the dynamic regulation of phosphorylation at T267/pS272 of SQSTM1, previously identified as ALPK3-dependent phosphosites^13^, across groups was consistent with the rescue of dysregulated proteostasis by *ALPK3* gene therapy (Extended Data Fig. 7e). While this does not directly implicate ALPK3 kinase activity, it further suggests that the proteostasis machinery is highly responsive to ALPK3 expression. Finally, proteasome function was significantly inhibited in the *Alpk3*^*W1538X*^ model (Extended Data Fig. 5l) and was restored in mice treated with *ALPK3* gene therapy. In sum, truncating variants in *Alpk3* impair both global and sarcomere-targeted proteostasis, and *ALPK3* gene therapy directly rescues this phenotype. This finding suggests that *ALPK3* gene therapy has the potential to restore protein quality control in diverse cardiomyopathies.

**Fig. 4:**
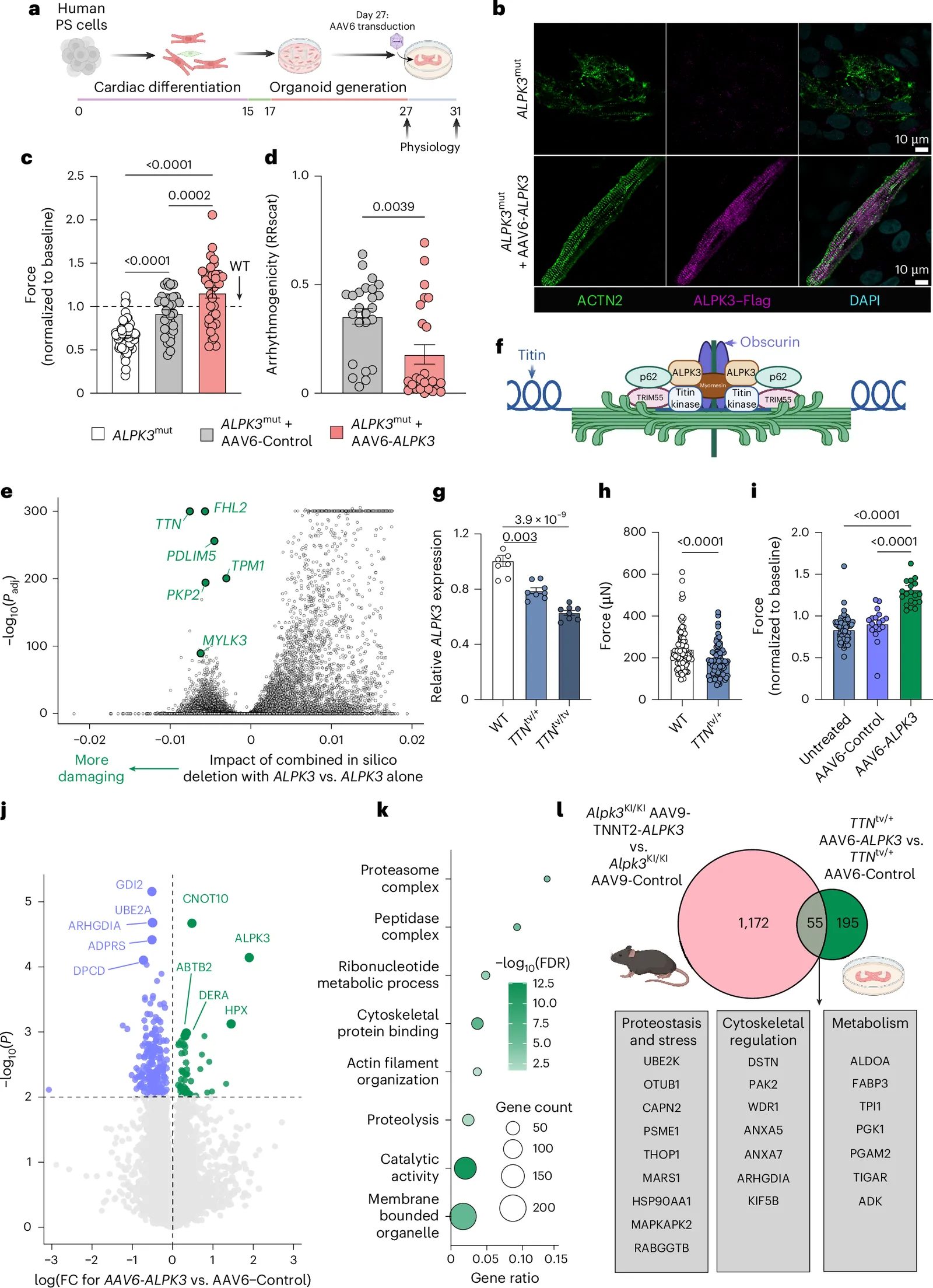
Contractile function of human pluripotent stem cell-derived cardiac organoid models of cardiomyopathy is restored by AAV-*ALPK3* treatment. **a**, Schematic of organoid experiments to test restoration of function by AAV-*ALPK3* restoration of function in cardiac organoids. PS, pluripotent stem. **b**, Delivery of AAV6-*ALPK3* to *ALPK3*^mut^ human pluripotent stem cell-derived cardiac monolayers resulted in correct localization of ALPK3 to the M band and restored cardiomyocyte structure. **c**, Organoid force at 96 h of treatment normalized to baseline (WT is dashed line, *n* = 85 untreated, 32 AAV6-Control and 34 AAV6-*ALPK3* individual organoids over three independent experiments; one-way ANOVA with Tukey’s multiple comparisons test). **d**, AAV6-*ALPK3* also reduced the frequency of arrhythmic events as measured by abnormalities in the peak-to-peak intervals (*n* = 24 individual organoids over three independent experiments; two-tailed unpaired *t* test). **e**, Geneformer-based AI prediction of the most damaging gene deletions in tandem with *ALPK3* deletion. *P*_adj_, adjusted *P* value. **f**, Graphical representation of the ALPK3–TTN protein quality control hub at the M band. **g**, Pseudobulk analysis of snRNA-seq data from a WT, *TTN*^tv/+^, *TTN*^tv/tv^ isogenic allelic series of human iPS-cell derived cardiac cells demonstrates a TTN dose-dependent reduction in *ALPK3* expression (*n* = 8 single-nucleus experiments per group; multiple *t* tests with Benjamini–Hochberg false discovery test). **h**, Baseline force analysis comparing *TTN*^tv/+^ organoids and their isogenic controls (*n* = 86 for control and 84 for *TTN*^tv/+^ organoids from four independent experiments; two-tailed Welch’s *t* test). **i**, Organoid force at 96 h of treatment normalized to baseline for *TTN*^tv/+^ cardiac organoids treated with AAV6-*ALPK3* (untreated, *n* = 70; AAV6-Control, *n* = 17; AAV6-*ALPK3*, *n* = 12; four independent experiments; ordinary one-way ANOVA with Tukey’s multiple comparison test). **j**,**k**, Volcano plot of differentially expressed proteins from *TTN*^tv/+^ organoids treated with AAV6-*ALPK3* versus AAV6-Control (**j**) and associated GO terms (**k**) (*n* = 4 per group). **l**, Overlap of differentially expression proteins identified from proteomics analysis of *TTN*^tv/+^ human organoids and mice (*Alpk3*^*W1538X*^ AAV9-TNNT2-*ALPK3* versus *Alpk3*^*W1538X*^ AAV9-Control). Mouse in panel **l** created in BioRender; McNamara, J. https://biorender.com/ifd59wl (2026). Source data

**Extended Data Fig. 8:**
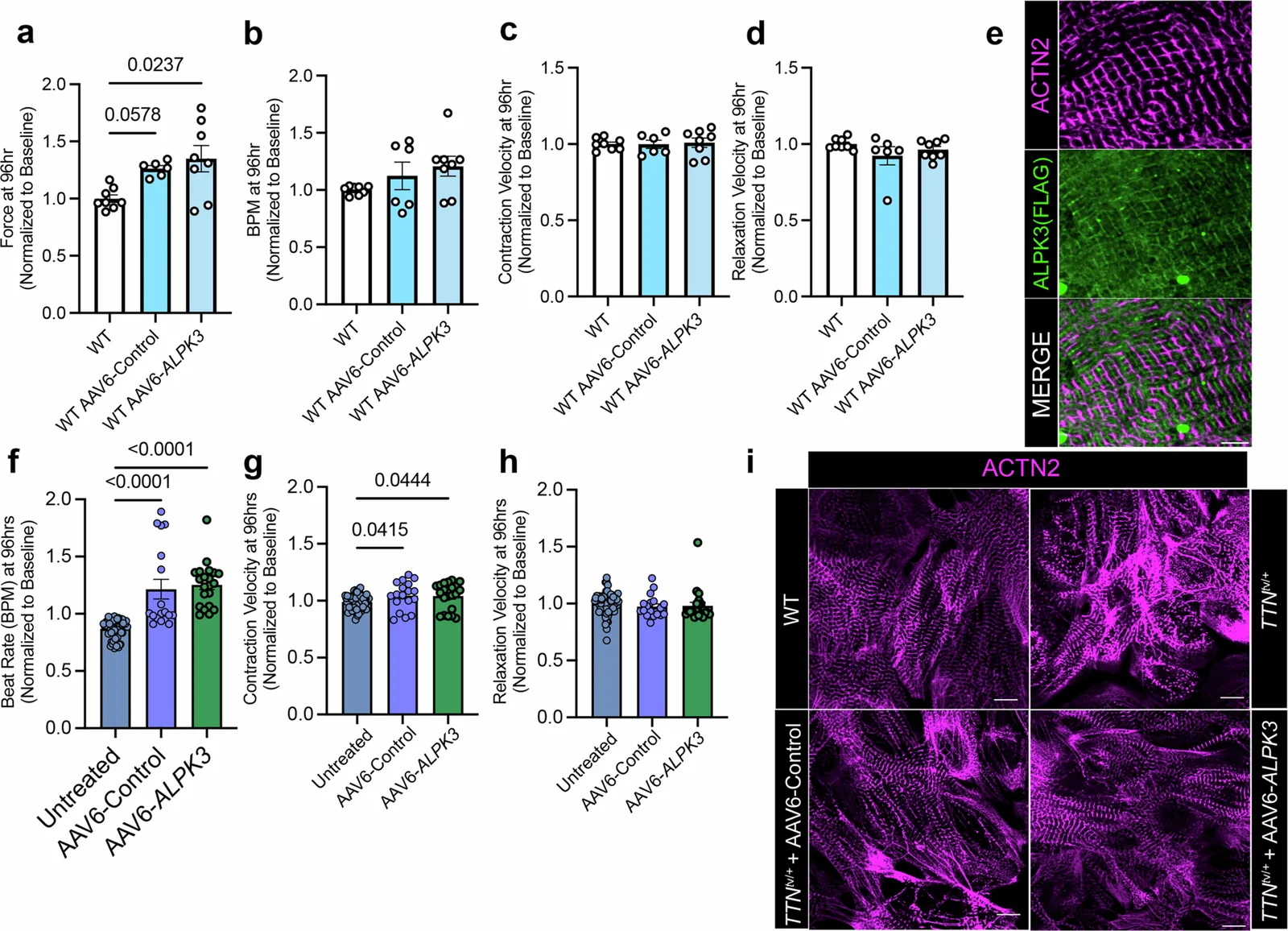
Assessment of ALPK3 gene therapy in control and *TTN*^tv/+^ cardiac organoids. **a**, Comparison of force, **b**, beating rate, **c**, contraction velocity, and **d**, relaxation velocity of untreated, AAV-Control, AAV-*ALPK3* WT cardiac organoids (WT, n = 8; WT AAV6-Control, n = 6; WT AAV6-Control; One-way ANOVA with Tukey’s multiple comparison test). **e**. Immunostaining of ACTN2 (purple) and ALPK3-FLAG (green= 5 µm) in WT iPSC-CM monolayers treated with AAV-CMV-*ALPK3-DDK* (scale bare. Additional *TTN*^tv/+^ cardiac organoid phenotyping data for relevant to Fig. 4i for **f**, beating rate, **g**, contraction velocity, and **h**, relaxation velocity (untreated, n = 71; AAV6-Control, n = 18; AAV-*ALPK3*, n = 21; four independent experiments; ordinary One-way ANOVA with Tukey’s multiple comparison test). **i**, ACTN2 staining of WT and *TTN*^tv/+^ cardiac monolayers either untreated or treated with AAV-Control or AAV-*ALPK3* (scale bar = 10 µm). Data are the mean ± s.e.m. Source data

To independently validate this therapeutic approach in a human model system, we differentiated a previously characterized^13^ human pluripotent stem cell line carrying a loss-of-function variant in *ALPK3* (*ALPK3*^mut^) into cardiac monolayers (Fig. 4a)^16^. The *ALPK3*^mut^ line exhibits severe sarcomere disorganization due to ALPK3 deficiency (Fig. 4b and ref. ^13^). *ALPK3*^mut^ cardiac cells were transduced with AAV-*ALPK3* (serotype 6) encoding a C-terminal Flag tag. As observed in vivo, the Flag epitope localized correctly to the sarcomeric M band, confirming proper translation and trafficking of full-length ALPK3 (Fig. 4b). Importantly, AAV-mediated *ALPK3* expression dramatically reversed the severe sarcomeric disorganization caused by the loss of *ALPK3*, as evidenced by restoration of ACTN2 organization (Fig. 4b). We next assessed whether AAV-mediated *ALPK3* expression could restore function in *ALPK3*^mut^ cardiac cells using a cardiac organoid platform^13,16,17^. Consistent with our previous findings^13^*, ALPK3*^mut^ organoids exhibited reduced systolic force generation and increased propensity for asynchronous beating relative to isogenic controls (Fig. 4c,d). We transduced organoids with 5 × 10^5^ viral genomes per cell on day 27 and assessed function at baseline and 96 h after transduction. While AAV6-Control (in which the *ALPK3* sequence is replaced with a noncoding sequence) induced a modest increase in contractile force, treatment with AAV6-*ALPK3* restored active force production to WT levels (Fig. 4c). AAV6-*ALPK3* treatment also reduced the prevalence of asynchronous beating events compared with AAV6-Control (Fig. 4d). The impact of ALPK3 was specific to *ALPK3*^mut^ organoids as WT cardiac organoids demonstrated no functional differences between AAV6-Control and AAV6-*ALPK3* (Extended Data Fig. 8a–d). Overexpression of ALPK3 in a WT background still resulted in the correct localization of ALPK3 to the cardiac M band (Extended Data Fig. 8e). These findings demonstrate that AAV6-*ALPK3* efficiently rescues contractile function in a human model of *ALPK3* cardiomyopathy.

Mechanistically, we and others have discovered that ALPK3 orchestrates a protein quality control hub at the sarcomere to maintain the turnover of contractile proteins^13–15^. We therefore reasoned that AAV6-*ALPK3* may have broader utility across other genetic cardiomyopathies that involve disrupted proteostasis^18^. To unbiasedly identify genetic cardiomyopathies that might benefit from *ALPK3* gene therapy, we applied the foundational artificial intelligence (AI) model Geneformer^19^ on a publicly available single-cell RNA sequencing dataset from fetal cardiomyocytes^20^ to assess genes that significantly alter the cardiomyocyte transcriptome upon in silico deletion in tandem with *ALPK3* compared with deletion of *ALPK3* alone (Fig. 4e). The most significantly damaging combination was deletion of *ALPK3* with *TTN* (encoding titin), while several other cardiomyopathy-linked genes were also detected (for example, *FHL2*, *PDLIM5*, *PKP2*, *TPM1*). *TTN-*truncating variants account for up to 25% of dilated cardiomyopathy cases, and, importantly, the M-band region of TTN has an interaction hub with ALPK3 involving sarcomeric proteins, as well as autophagy and proteasomal regulators at the M band of the sarcomere (Fig. 4f)^13,21^. Single-nucleus RNA sequencing (snRNA-seq) across human pluripotent stem cell-derived cardiomyocytes with an allelic series of truncating variants of *TTN* (including *TTN* c.72663delA; *TTN*^tv^) revealed a gene dose-dependent impact of *TTN*^tv^ on *ALPK3* expression, suggesting a potential regulatory link between these two genes (Fig. 4g). Cardiac organoids from the same heterozygous *TTN*^tv/+^ cell line demonstrated a significant deficit in force production compared with isogenic controls (Fig. 4h). AAV6-*ALPK3* significantly restored force production to WT levels in *TTN*^tv/+^ human cardiac organoids without impacting other physiological parameters (Fig. 4i and Extended Data Fig. 8f–h), confirming the hypothesis that *ALPK3* gene therapy has potential to treat a broader spectrum of cardiomyopathies. Immunofluorescence analysis further demonstrated that AAV6-*ALPK3* alleviated the proteinopathy of *TTN*^tv^ organoids, which has been well described (Extended Data Fig. 8i)^22^. Proteomic profiling of single *TTN*^tv/+^ cardiac organoids treated with either AAV6-Control or AAV6-*ALPK3* demonstrated that *ALPK3* plays a role of these genes in the regulation of several proteostasis and cytoskeletal pathways, such as proteolysis, the proteasome and ribonucleotide synthesis (Fig. 4j,k). To define the ALPK3-regulated proteome, we intersected significantly regulated proteins between this dataset and that of *Alpk3*^*W1538X*^ mice treated with control or *ALPK3* gene therapy. This revealed 55 shared proteins, which were largely related to cellular proteostasis, as well as cytoskeletal and metabolic regulation (Fig. 4l). Taken together, these data demonstrate the therapeutic potential of modulating proteostasis stress in genetic cardiomyopathy.

The use of an oversized AAV vector such as AAV9-TNNT2-*ALPK3* is an important consideration for a clinical development program. Importantly, our oversized construct resulted in an expression of full-length ALPK3 and showed high efficacy, supporting the clinical translatability of this approach. The feasibility of clinical development of oversized AAV constructs is further supported by the clinical approval and therapeutic success of Roctavian for the treatment of hemophilia^23^. An alternative strategy for larger genes is to miniaturize the protein while retaining key functional domains, an approach that has been championed in gene therapies for Duchenne muscular dystrophy^24^. Recent work has described a miniALPK3 construct delivered by the myoAAV capsid, which partially restored cardiac function in *Alpk3-*knockout mice treated neonatally via retro-orbital injection^25^. However, the miniALPK3 construct offered only partial functional rescue in a neonatal protection model (fractional shortening: WT, ~ 45%; *Alpk3*⁻^/^⁻, ~ 22%; *Alpk3*⁻^/^⁻ + myoAAV-miniALPK3, ~ 37%)^25^. In comparison, neonatal injection at a similar dose using full-length AAV9-TNNT2-*ALPK3* in this study, although the construct was delivered via the less efficient intraperitoneal injection route, resulted in near-complete prevention of cardiac dysfunction (fractional shortening: WT, ~ 27%; *Alpk3*^*W1538X*^, ~ 14%; *Alpk3*^*W1538X*^ + AAV9-TNNT2-*ALPK3*, ~ 27%). Furthermore, we observed complete restoration of function in an adult disease-reversal model (fractional shortening (%): WT ~ 26%; *Alpk3*^KI/KI^ ~ 14%; *Alpk3*^KI/KI^ + AAV9-TNNT2-ALPK3 ~ 27%). While this is based on a cross-study comparison between two independent studies that used different mouse models, viral capsids and delivery routes, these findings raise the possibility that the miniALPK3 construct may not fully recapitulate the activity of full-length ALPK3. Further studies would be required to systematically assess the functionality of the intrinsically disordered region deleted in the miniALPK3 construct. Additionally, miniaturized gene therapies have the potential for the generation of immune-modulatory ‘neo-epitopes’ at the junctions created by domain fusion of the miniaturized protein. For example, in microdystrophin constructs developed for Duchenne muscular dystrophy, the miniaturized proteins have been reported to provoke immune responses, resulting in inflammatory myopathy and transient transgene expression^26^. Although this does not preclude the success of miniaturized constructs, it highlights the importance of carefully evaluating immunogenicity and long-term safety. Whether similar considerations will apply to miniALPK3 constructs in human therapy remains to be determined. Therefore, while reducing vector genome size is an important consideration for the development of *ALPK3* gene therapies, our data support the use of full-length *ALPK3*.

Herein, we show that *ALPK3* gene therapy effectively restores contractile function and cardiac structure in both in vivo and in vitro models of *ALPK3* cardiomyopathy. Strikingly, in a mouse model of *ALPK3* cardiomyopathy, AAV9-TNNT2-*ALPK3* not only prevented cardiomyopathy when delivered to neonatal mice but also completely abrogated pathology in adult mice with established cardiomyopathy. This level of rescue is seldom observed in mouse models with established cardiomyopathy^6,8^ and suggests that the heart is very sensitive to the expression levels of ALPK3. Given that the *Alpk3*^*W1538X*^ model does not develop substantial interstitial fibrosis despite the severity of disease, future studies will be required to determine whether this level of functional rescue is still observed in hearts with pre-existing fibrosis. These studies used homozygous *Alpk3*^*W1538X*^ mice, which model the rare and severe autosomal recessive disease in humans rather than the more common autosomal dominant disease, which accounts for ~2% of all hypertrophic cardiomyopathy cases. We, and others, have not observed abnormal phenotypes in young heterozygous *Alpk3*^*W1538X*^ mice. Indeed, hypertrophic cardiomyopathy is only detectable in heterozygous *Alpk3*^+/–^ mice only after 12 months of age^14^. While this scenario models the patient trajectory well, it is not readily amenable to therapeutic testing. In this context, *ALPK3* gene therapy development is analogous to the development of *MYBPC3* gene therapies, for which phase 1b/2 clinical trials (MyPEAK-1)^27^ to assess safety and efficacy are focused on the autosomal dominant patient population, despite preclinical work using more severe homozygous knockout mice^6^. Thus, the use of homozygous animal models in preclinical studies to provide rapid proof-of-concept support for therapeutic efficacy is an established paradigm for myotropic gene therapies^6,8^.

Moreover, *ALPK3* gene therapy may have utility beyond this single indication due to the role of ALPK3 in coordinating a protein quality control hub at the M band of the sarcomere^13^. Crucially, we demonstrate that *ALPK3* gene therapy restores function in human cardiac organoids harboring *TTN*-truncating variants, the most common genetic cause of dilated cardiomyopathy. This is particularly important given that the extreme size of the *TTN* gene (>100,000 nucleotides) renders it unsuitable for conventional gene replacement therapy. These findings establish *ALPK3* gene therapy as a promising therapeutic approach for both *ALPK3* and other genetic cardiomyopathies. It will be important to determine the range of cardiomyopathies with disrupted sarcomeric proteostasis that could benefit from *ALPK3* gene therapy.

## Methods

### Ethics statement

All human stem cell work was approved by the Royal Children’s Hospital Human Research Ethics Committee (33001A and 93025). Mouse experiments were approved by the Animal Ethics Committee of the Murdoch Children’s Research Institute (A978). Research was carried out in accordance with the relevant guidelines and regulations provided by the National Health and Medical Research Council of Australia (the National Statement on Ethical Conduct in Human Research and the Australian Code for the Care and Use of Animals for Scientific Purposes).

### *Alpk3*^*W1538X*^ mouse model

The *Alpk3*^*W1538X*^ mouse model was described previously^13^ and established to recapitulate the initial *ALPK3* cardiomyopathy families identified^13^. Mice were maintained on a C57BL/6J genetic background, fed standard chow, given water ad libitum and maintained in a 12-h light–12-h dark cycle at ambient room temperature. Mice were genotyped using a custom probe set established by Transnetyx. All experiments were performed and analyzed by a researcher blinded to the treatment regimen. The number of mice used in each experiment is provided in the figure legends.

### Neonatal mouse gene therapy study protocol

Both WT and homozygous *Alpk3*^*W1538X*^ mice were used in this study (WT, *n* = 6 (4 male, 2 female); *Alpk3*^*W1538X*^ AAV9-Control, *n* = 7 (1 male, 6 female); *Alpk3*^*W1538X*^ AAV9-TNNT2-*ALPK3*, *n* = 6 (3 male, 3 female)). Mice were randomized into treatment groups (AAV9-Control or AAV9-TNNT2-*ALPK3*; VectorBuilder) on P0. AAV was delivered to P0 pups via intraperitoneal injection at a dose of 2.5 × 10^11^ viral genomes or ~2 × 10^14^ viral genomes per kg. From 4 weeks of age, mice underwent biweekly echocardiography to assess both systolic and diastolic function as described^13^. At 12 weeks of age, mice were culled, and tissues were collected for downstream analysis.

### Adult mouse gene therapy study protocol

Both WT and homozygous *Alpk3*^*W1538X*^ mice were used in this study; however, only *Alpk3*^*W1538X*^ mice were treated with AAV9 based on the minimal impact observed in WT mice in the neonatal study. Six-week-old mice (WT, *n* = 8 (4 male, 4 female); *Alpk3*^*W1538X*^ AAV9-Control, *n* = 8 (5 male, 3 female); *Alpk3*^*W1538X*^ AAV9-TNNT2-*ALPK3*, *n* = 9 (5 male, 4 female)) underwent baseline echocardiography to assess heart function and were then treated with 1 × 10^14^ viral genomes per kg of either AAV9-Control or AAV9-TNNT2-*ALPK3* via tail vein injection (VectorBuilder). From 10 weeks of age, cardiac function was assessed by echocardiography every 2 weeks. At 18 weeks of age, mice were culled and tissues were collected for downstream analysis.

### Echocardiography

Mouse echocardiography was performed as previously described^13^. Briefly, mice were anesthetized using 2–3% isoflurane and then placed in the supine position on the heated imaging stage under 1–2% isoflurane. Echocardiography was performed using a VisualSonics Vevo 3100 ultrasound machine with an MS400 18- to 38-MHz transducer (Fujifilm) under 1–2% isoflurane anesthesia. Systolic function was reported based on parasternal short-axis M-mode imaging, and diastolic function was assessed by transmitral four-chamber-view Doppler imaging. All data collection was performed blinded to the treatment group.

### Histology and immunofluorescence analysis

Following the dissection of experimental mice, mouse hearts were first perfused with PBS containing 0.1 M KCl to arrest hearts in diastole and then fixed in 4% paraformaldehyde (PFA) for ~2 h on ice. Hearts were then cryoprotected by sequential incubation with 15% sucrose and 30% sucrose in PBS before embedding in OCT. Ten-micrometer sagittal sections were adhered to Superfrost Plus glass slides and stored at −80 °C until use. Slides were brought to room temperature, rinsed with PBS and then simultaneously permeabilized and blocked (5% FBS in PBS with 0.1% Tween-20, 0.1% Triton X-100). Sections were then stained using the antibodies outlined in the Reporting Summary and imaged on either a Zeiss LSM900 or Zeiss Z2 Axio Imager. For quantification of cardiomyocyte cross-sectional area, images were taken from multiple regions of the heart and the area of myocytes in a clear cross-sectional orientation (identified as largely round and uniform in shape) was assessed using ImageJ.

### Human pluripotent stem cell culture and differentiation

For *ALPK3*^mut^ human pluripotent stem cell studies, the female HES3 (WiCell ES03) *NKX2-5*^eGFP/+^ human embryonic stem cell line was used, which has been previously described^28^. The loss-of-function stem cell line has previously been described to exhibit reduced contractile function and impaired rhythmicity^3,13^. Stem cells were maintained as previously described. Briefly, stem cells were cultured in mTeSR Plus culture medium (Stem Cell Technologies, 05825) in culture flasks coated with human embryonic stem cell-qualified Matrigel (CFAL354277) following the manufacturer’s protocols. Cells were passaged twice weekly using TrypLE (Thermo Fisher Scientific, 12604013). Pluripotent stem cells were differentiated in a monolayer as previously described ^13^. Cells were plated on day −4 with the medium changed to mTeSR-1 medium (Stem Cell Technologies, 85850) on day −1. On days 0, 1 and 2, cells were changed to RPMI-1640 plus GlutaMAX (Thermo Fisher Scientific, 61870-036) containing 500 μM AA2P, 10 U ml^−1^ penicillin-streptomycin and 2% B27 supplement minus insulin (Thermo Fisher Scientific, A1895601), 5 ng ml^−1^ BMP-4 (R&D Systems, 314BP050CF), 9 ng ml^−1^ Activin A (R&D Systems, 388AC050), 5 ng ml^−1^ bFGF (R&D Systems, 233FB025) and 1 μM CHIR99021 (Tocris, 4423). On Day 3, the medium was changed to the same RPMI-1640 + B27 minus insulin medium but containing 5 μM IWP-4 (Stem Cell Technologies, 72554). On day 6, cells were changed to RPMI-1640 + B27 medium containing insulin (Thermo Fisher Scientific, 17504044) and 5 μM IWP-4 with the medium changed every 2 days. On day 13, the medium was switched to RPMI-1640 + B27 medium containing insulin with no additions, and cells were harvested for subsequent processing on day 15.

### Cardiac organoid fabrication and viral transduction

On day 15 of differentiation, cardiac cells were harvested for the fabrication of cardiac organoids as described^13,17^. Briefly, Heart-Dyno culture inserts were manufactured with a 10:1 ratio of base to curing agent polydimethylsiloxane SYLGARD 184 silicone elastomer. Before seeding of cells, the culture inserts were coated with 3% BSA in PBS to prevent sticking of cells. Then, 5 × 10^4^ cardiac cells in serum-free medium (MEMα GlutaMAX supplemented with 200 μM AA2P, 1% penicillin-streptomycin, 4% (v/v) B27 with insulin, 10 ng ml^−1^ FGF-2 and 10 ng ml^−1^ PDGF-BB (R&D Systems))^17^ were seeded in 3.5 μl of a matrix mixture of 2.6 mg ml^−1^ acid-solubilized collagen I (Devro) that was salt balanced with 10× DMEM (Thermo Fisher Scientific) and pH neutralized with 0.1 M NaOH and 9% v/v Matrigel. Cardiac organoids were allowed to gel for 30–45 min, after which 150 μl of serum-free medium was added to each well. On day 17, the organoids were switched to maturation medium^17^ with the medium changed every second day until a switch to weaning medium^17^ on day 22; the organoids were maintained until viral transduction on day 27. Organoids were transduced with 5 × 10^5^ viral genomes per cell for 24 h. Functional assays were performed at baseline and 96 h after treatment.

### Organoid function

Organoid force and kinetics in cardiac organoids were assessed by tracking deflection of the elastic poles of each organoid. To perform this, 10-s videos (50 frames per second) were taken of each organoid using a ×5 objective on a Leica Thunder microscope^13^. Organoids were maintained in a 37 °C, 5% CO_2_ environment to prevent changes in contractile behavior. Contractility analysis was performed using Tempo.ai, developed by Dynomics^29^. Tempo.ai provides maximum force, beat rate (BPM) and kinetics, including activation and relaxation time. Arrhythmia is assessed using the rrscat variable, which measures the variability in the time intervals between successive force peaks.

### Geneformer prediction of the most damaging gene deletion combinations

We leveraged Geneformer^19^, a foundational AI model for network biology, to predict the impact of in silico deletion of genes in combination with in silico deletion of *ALPK3* on the cell state of human fetal cardiomyocytes from the Fetal Cell Atlas^20^. In silico deletion was performed using Geneformer’s InSilicoPerturber function, deleting each gene in combination with *ALPK3* and comparing the predicted impact to that of deleting *ALPK3* alone. InSilicoPerturber quantifies the predicted impact of in silico deletion as the cosine shift of the perturbed cell embedding from the original cell embedding within the Geneformer embedding space. This analysis was performed as zero-shot in silico perturbation using the Geneformer-V1 model directly without fine-tuning. The relative impact of combinations compared with random combinations was quantified and tested for statistical significance by Wilcoxon rank-sum tests with Benjamini–Hochberg correction.

### Proteomics and phosphoproteomics sample preparation

Snap-frozen left ventricular tissue was powdered and weighed under liquid nitrogen. Between 10–20 mg of powdered tissue was resuspended in 200 µl of 4% SDC lysis buffer (4% sodium deoxycholate in 100 mM Tris pH 8.5) and was heated at 95 °C for 5 min with shaking at 1,500 rpm. Once cool, tubes were spun briefly and each sample was sonicated with 30% output for a total of 15 s (5 rounds of 3 s on, 3 s off). Following centrifugation (14,000×*g* for 15 min at room temperature), the protein concentration of the supernatant was measured by BCA assay and samples were diluted to 2 mg ml^−1^ in SDC lysis buffer to a final volume of 180 µl. Samples were then reduced and alkylated with 20 µl of fresh TCEP/CAA buffer (400 mM 2-chloroacetamide, 87 mM TCEP-HCl in 400 mM KOH) at 45 °C for 5 min with shaking at 1,500 rpm. After cooling, proteins were precipitated via sequential addition of 800 µl of 100% methanol, 200 µl of 100% chloroform and 600 µl MS-grade water. After centrifugation, the top 80% was discarded and the protein was washed with 600 µl of 100% methanol and centrifuged again to pellet the protein. The resultant pellet was air-dried, resuspended in 190 µl SDC lysis buffer and sonicated again. Then, 10 µl of NaOH was mixed, followed by 840 µl ice-cold 100% acetone, and samples were allowed to precipitate overnight. The next day, samples were centrifuged at 20,000×*g* for 15 min at 4 °C, and the supernatants were discarded. After airdrying the protein pellets, each sample was resuspended in 155 µl of SDC lysis buffer and sonicated again. Protein concentration was measured by BCA assay and normalized across all groups for subsequent processing for MS. Two milligrams of protein was processed with the EasyPhos method as previously described^30^.

### Liquid chromatography and tandem MS measurement

Proteomics. MS loading buffer (2% acetonitrile (ACN), 0.3% trifluoracetic acid (TFA)) was loaded onto in-house-fabricated, 55-cm columns with a 75-µm internal diameter (ID) and packed with 1.9 µM C18 ReproSil Pur AQ particles using a Neo Vanquish HPLC instrument coupled to an Orbitrap Astral mass spectrometer (Thermo Fisher Scientific). Column temperature was maintained at 60 °C in a Sonation column oven, and peptides were separated using a binary buffer system comprising 0.1% formic acid (buffer A) and 80% ACN plus 0.1% formic acid (buffer B), at a flow rate of 400 nl min^−1^ with a gradient of 5–35% buffer B over 28 min followed by 35–60% buffer B over 7 min, resulting in ~35 min gradients. Peptides were analyzed with one full scan (380–980 *m*/*z*, *R* = 240,000) at a target of 5 × 10^6^ ions, followed by 300 data-independent acquisition (DIA) MS/MS scans (381–979 *m*/*z*) with HCD (target of 5 × 10^4^ ions, maximum injection time of 3.5 ms, isolation window of 2 *m*/*z*, NCE of 23%), with fragments detected in the Astral mass spectrometer.

Phosphoproteomics. Enriched phosphopeptides in MS loading buffer (2% ACN, 0.3% TFA) were loaded onto in-house fabricated 55-cm columns with a 75-µm I.D. and packed with 1.9 µM C18 ReproSil Pur AQ particles using a Neo Vanquish HPLC instrument coupled to an Orbitrap Astral mass spectrometer (Thermo Fisher Scientific). Column temperature was maintained at 60 °C in a Sonation column oven, and peptides were separated using a binary buffer system comprising 0.1% formic acid (buffer A) and 80% ACN plus 0.1% formic acid (buffer B), at a flow rate of 400 nl min^−1^ with a gradient of 3–19% buffer B over 20 min followed by 19–41% buffer B over 10 min, resulting in ~30-min gradients. Peptides were analyzed with one full scan (380–980 *m*/*z*, *R* = 240,000) at a target of 5 × 10^6^ ions, followed by 300 DIA MS/MS scans (381–979 *m*/*z*) with HCD (target of 5 × 10^4^ ions, maximum injection time of 3.5 ms, isolation window of 2 *m*/*z*, NCE of 23%), with fragments detected in the Astral mass spectrometer.

### Proteome preprocessing

Raw data were analyzed using MaxDIA (MaxQuant v2.6.7.0) in discovery DIA mode. A spectral library was generated against the mouse UniProt FASTA database (accessed in March 2025) with inclusion of the human ALPK3 sequence (UniProt ID: Q96L96), common contaminants and iRT standards for protein identification. Default settings were used for search parameters. The false discovery rate (FDR) for peptide-spectrum matches and protein identifications was set to 1% at the peptide level. Data analysis was performed in R v4.4.2. Proteins identified as contaminants or decoy sequences or detected in fewer than 10 of 15 samples were excluded from further analysis (66% presence). Label-free quantification (LFQ) intensities were log_2_ transformed and normalized using a median-centering approach.

For comparison of human ALPK3 versus mouse ALPK3 expression (Extended Data Fig. 5d), AAV-*ALPK3* raw data samples were analyzed using MaxDIA (MaxQuant v2.6.7.0) in discovery DIA mode, where a spectral library was generated against the human UniProt FASTA database (accessed in March 2025) with inclusion of common contaminants and iRT standards for protein identification. Default settings were used for search parameters. The FDR for peptide-spectrum matches and protein identifications was set to 1% at the peptide level. AAV-Control and untreated raw data samples were analyzed using MaxDIA (MaxQuant v2.6.7.0) in discovery DIA mode, where a spectral library was generated against the mouse UniProt FASTA database (accessed in March 2025) with inclusion of common contaminants and iRT standards for protein identification. Default settings were used for search parameters. The FDR for peptide-spectrum matches and protein identifications was set to 1% at the peptide level. Data analysis was performed in R v4.4.2. Proteins identified as contaminants or decoy sequences were excluded from further analysis. Intensity-based absolute quantification (iBAQ) intensities for human and mouse experiments were log_10_ transformed, independently median centered and then re-anchored to the prenormalization median of the combined dataset to enable direct cross-species comparison.

For TTN cardiac organoids, raw data were analyzed using MaxDIA (MaxQuant v2.6.7.0) in discovery DIA mode. A spectral library was generated against the mouse UniProt FASTA database (accessed in March 2025) with inclusion of common contaminants and iRT standards for protein identification. Default settings were used for search parameters. The FDR for peptide-spectrum matches and protein identifications was set to 1% at the peptide level. Data analysis was performed in R v4.4.2. Proteins identified as contaminants or decoy sequences or detected in fewer than six of eight samples were excluded from further analysis (75% presence). LFQ intensities were log_2_ transformed and normalized using a median-centering approach.

### Phosphoproteome preprocessing

Raw data were analyzed using direct DIA analysis in Spectronaut software (version 19.3.2410). A spectral library was generated against the mouse UniProt FASTA database (accessed in March 2025). Default settings were used for search parameters. The following preprocessing and analyses were performed in R software environment version 4.4.2. To remove unreliable phosphosites, we first removed all phosphosites that had a localization probability of <0.5; then any remaining phosphosites with a maximum localization probability of <0.75 were further removed (retaining only class I phosphosites). We further filtered out any phosphosites that were missing in at least 66% of all samples, and all values < 3 were converted to missing values as they were considered unreliable measurements indistinguishable from background noise. Raw intensities were log_2_ transformed, after which we performed median-centering normalization.

### DNA and RNA isolation and qPCR

Total RNA was extracted using TRIzol, followed by cleanup using the Qiagen mRNeasy kit according to the manufacturer’s protocol. cDNA was transcribed using SuperScript IV VILO Master Mix (Thermo Scientific, 11756050) and diluted 1:10 in nuclease-free water. DNA was isolated using the Qiagen DNeasy blood and tissue kit (69504) and diluted to 5 ng µl^−1^. SYBR Green assays were performed using QuantiNova SYBR Green PCR with the primer sequences (Integrated DNA Technologies) described in Supplementary Table 3. DNA or RNA copy number was calculated from a standard curve obtained using *ALPK3* plasmid DNA with the concentration spanning 8 orders of magnitude.

### Measurement of proteasome activity

Proteasome activity was assessed as described^31^. Briefly, 30–50 mg of tissue was pulverized while frozen and resuspended in 5 volumes of Milli-Q water containing Roche cOmplete EDTA-free protease inhibitor. After three freeze–thaw rounds with liquid nitrogen, tissue was homogenized with a rotor-stator homogenizer and centrifuged at 18,000×*g* at 4 °C for 30 min. The concentration of the supernatant was measured using the BCA assay. Thirty milligrams of protein was used to assess proteasome activity following the manufacturer’s protocol (ab107921).

### SciPlex snRNA-seq

To assess *ALPK3* gene expression in *TTN*^tv^ iPSC cardiomyocytes, we performed single-cell combinatorial indexing RNA sequencing on SciPlex, a massively parallel snRNA-seq platform based on combinatorial barcoding, as described^32^. Briefly, nuclei were isolated from cardiac monolayers using cell lysis buffer (10 mM Tris-HCl (pH 7.4), 10 mM NaCl, 3 mM MgCl_2_ + 0.1% IGEPAL CA-630 + 1% SUPERase•In RNase inhibitor + 0.02% ultrapure BSA). Replicates were labeled with well-specific hashing oligonucleotides on ice to enable sample multiplexing. Nuclei were subsequently fixed in 4% PFA on ice for 15 min and pooled. Following fixation, nuclei were permeabilized in buffer containing 10 mM Tris-HCl (pH 7.4), 10 mM NaCl and 3 mM MgCl_2_ + 0.02% ultrapure BSA + 0.25% Triton X-100. Combinatorial multiplexing was performed using sequential rounds of reverse transcription and ligation-based barcoding, with nuclei redistributed between wells at each step to generate unique barcode combinations. Following tagmentation and cDNA purification, sequencing libraries were amplified using P5 and P7 indexing primers. Hashing oligonucleotide and transcriptome libraries were pooled and sequenced on the same S2 flow cell lane of a NovaSeq 6000 platform.

Nucleus-derived transcriptomes were aligned to the human reference genome GRCh38 with 10x Genomics Cell Ranger (v7.1.0). Technical artifacts (for example, empty droplets or ambient RNA) were removed using CellBender (v2.2.0). All downstream analyses were performed using Seurat v5. Doublets were identified and removed using DoubletFinder^33^, and nuclei with >5% mitochondrial transcript content were excluded and counted using Cell Ranger (v7.1.0). Following this, counts were aggregated to create a table of unique molecular identifier (UMI) counts for 33,939 genes for each of the samples. All preprocessing and filtering steps for the datasets were carried out using the R statistical programming language (v3.6.0). Differential gene expression analysis was performed on aggregated pseudobulk profiles using the limma R package (v3.40.2). Statistical testing was performed using moderated *t* tests, accounting for the mean–variance relationship and applying robust empirical Bayes shrinkage of variance estimates. Differentially expressed genes (DEGs) were defined using TREAT tests with an FDR threshold of <0.05.

### Genome editing

The *TTN* c.72663delA (p.Pro24223fs) iPS cell line was generated by the Murdoch Children’s Research Institute iPSC Derivation and Gene Editing Core. PB010.5 iPS cells from a healthy donor were edited using a Cas9-Gem mRNA sgRNA (GCGGATCAGGGGGGTCCATA, cloned into the pSMART vector) and ssODN (CTGTAAATAAATATGGAGTGGGAGAGCCACTGGAATCAGAGCCTGTGCTTGCAGTGAATCC**A**TATGG**-**CCCCCTGATCCGCCCAAAAACCCTGAAGTGACAACTATTACTAA; synonymous T > A variant and delA locations are bolded; Integrated DNA Technologies). Cells were nucleofected with these contructs using the Neon Transfection System (1,100 V, 30 ms, 1 pulse) and plated over three wells of a Matrigel-coated six-well dish in mTesR+ medium supplemented with 10 µM Y-27632, which was removed from the medium 4 h after transfection. Gene-edited iPS cells were identified by next-generation sequencing of PCR amplicons generated with primers flanking the mutation (TTN_scF: atacatattccgtgtcatgg and TTN_scR: ctttatcacgccgttccac). Lines were tested for mycoplasma, confirmed to have a normal karyotype by SNP array and verified for pluripotency by flow cytometry of OCT3/OCT4, TRA-1-60, SSEA-6 and TRA-1-81.

### Statistics and reproducibility

The investigators were blinded to treatment groups during experiments. No statistical methods were used to predetermine the sample size. The specific statistical tests used are presented within the figure legends. For the comparison of two experimental groups, two-tailed unpaired *t* tests were used. When comparing multiple groups, one-way analysis of variance (ANOVA) with Tukey’s multiple comparisons test was applied. When comparing multiple groups across timepoints, we applied two-way ANOVA with Tukey’s multiple comparisons test. *P* < 0.05 was considered statistically significant. For single-nucleus RNA sequencing, statistical testing was performed using moderated *t* tests, accounting for the mean–variance relationship and applying robust empirical Bayes shrinkage of variance estimates. DEGs were defined using TREAT tests with an FDR threshold of <0.05. For confocal microscopy analysis, representative figures are shown, and experiments were performed a minimum of two times. All statistical analyses were performed using either RStudio (version 2024.12.1) or GraphPad Prism (version 10.6.1).

### Reporting summary

Further information on research design is available in the Nature Portfolio Reporting Summary linked to this article.

## Supplementary information

Reporting Summary

Peer Review File

Supplementary Tables 1–3 Supplementary Table 1. Neonatal mouse study echocardiography parameters. Supplementary Table 2. Adult mouse study echocardiography parameters. Supplementary Table 3. List of qPCR primers.

## Source data

Source Data Fig. 1 Statistical source data.

Source Data Fig. 2 Statistical source data.

Source Data Fig. 3 Statistical source data.

Source Data Fig. 4 Statistical source data.

Source Data Extended Data Fig. 1 Statistical source data.

Source Data Extended Data Fig. 2 Statistical source data.

Source Data Extended Data Fig. 4 Statistical source data.

Source Data Extended Data Fig. 5 Statistical source data.

Source Data Extended Data Fig. 6 Statistical source data.

Source Data Extended Data Fig. 7 Statistical source data.

Source Data Extended Data Fig. 8 Statistical source data.

## Data Availability

RNA sequencing data have been deposited in the Gene Expression Omnibus (GEO), accession number GSE328808. Proteomics data are available at PRotein IDEntifications Database (PRIDE), accession number PXD077620 (https://www.ebi.ac.uk/pride/archive/projects/PXD077620). All other data are available within the supporting files and upon reasonable request to the corresponding authors. Source data are provided with this paper.
