# Supplementary material for: Alpha protein kinase 3 gene therapy restores heart function in mouse and human models of cardiomyopathy
Source: Nat Cardiovasc Res. 2026 Aug 19;5(9):795–806. doi: 10.1038/s44161-026-00843-1 (PMC13561881; doi:10.1038/s44161-026-00843-1)
Supplement: Supplementary file 1 [file 44161_2026_843_MOESM1_ESM.pdf]

nature portfolio

Corresponding author(s): James W. McNamara
Enzo R. Porrello
David A. Elliott

Last updated by author(s): Jun 18, 2026

Reporting Summary

Nature Portfolio wishes to improve the reproducibility of the work that we publish. This form provides structure for consistency and transparency in reporting. For further information on Nature Portfolio policies, see our Editorial Policies and the Editorial Policy Checklist.

Statistics

For all statistical analyses, confirm that the following items are present in the figure legend, table legend, main text, or Methods section.

n/a	Confirmed	
☐	☒	The exact sample size (*n*) for each experimental group/condition, given as a discrete number and unit of measurement
☐	☒	A statement on whether measurements were taken from distinct samples or whether the same sample was measured repeatedly
☐	☒	The statistical test(s) used AND whether they are one- or two-sided<br>*Only common tests should be described solely by name; describe more complex techniques in the Methods section.*
☐	☒	A description of all covariates tested
☐	☒	A description of any assumptions or corrections, such as tests of normality and adjustment for multiple comparisons
☐	☒	A full description of the statistical parameters including central tendency (e.g. means) or other basic estimates (e.g. regression coefficient) AND variation (e.g. standard deviation) or associated estimates of uncertainty (e.g. confidence intervals)
☐	☒	For null hypothesis testing, the test statistic (e.g. *F*, *t*, *r*) with confidence intervals, effect sizes, degrees of freedom and *P* value noted<br>*Give P values as exact values whenever suitable.*
☒	☐	For Bayesian analysis, information on the choice of priors and Markov chain Monte Carlo settings
☒	☐	For hierarchical and complex designs, identification of the appropriate level for tests and full reporting of outcomes
☒	☐	Estimates of effect sizes (e.g. Cohen's *d*, Pearson's *r*), indicating how they were calculated

Our web collection on statistics for biologists contains articles on many of the points above.

Software and code

Policy information about availability of computer code

Data collection	Mouse Echocardiography<br>We used a FujiFILM VisualSonics Vevo3100 ultrasound machine for collection of all echocardiography data with vevo software for M-Mode, B-Mode, and Doppler image collection.<br><br>Quantitative Reverse Transcriptase Polymerase Chain Reaction<br>qPCR data was obtained using a Roche LightCycler 480 II.<br><br>Human cardiac organoids<br>We used a Leica Thunder imaging microscope with LasX software to collect stacked imaged tiffs of contracting human cardiac organoids (10 seconds at 50 frames per second).<br><br>Immunostaining<br>We collected images of cardiac monolayers using a Zeiss LSM980 with ZEISS ZEN Black software (version 2.3 SPI)
Data analysis	All statistical analysis was performed in either Graphpad Prism Version 10.6.1 or RStudio Version 2024.12.1<br>Echocardiography was analysed in FUJIFILM VisualSonics VevoLab<br>Immunostaining was analysed and processed using FIJI (ImageJ2) Version 2.14.0/1.54f<br>Organoid contractility stacked TIFFs were analysed using a commercial software, Tempo.ai, developed by Dynomics.

Immunostaining
Immunofluorescent images were processed using Fiji ImageJ (version: 2.3.0/l.53q).

For manuscripts utilizing custom algorithms or software that are central to the research but not yet described in published literature, software must be made available to editors and reviewers. We strongly encourage code deposition in a community repository (e.g. GitHub). See the Nature Portfolio guidelines for submitting code & software for further information.

Data

Policy information about availability of data

All manuscripts must include a data availability statement. This statement should provide the following information, where applicable:

- Accession codes, unique identifiers, or web links for publicly available datasets
- A description of any restrictions on data availability
- For clinical datasets or third party data, please ensure that the statement adheres to our policy

RNA sequencing data have been deposited in the Gene Expression Omnibus (GEO), accession number GSE328808. Proteomics data are available at PRotein IDEntifications Database (PRIDE), accession number PXD077620. All other data is available upon reasonable request to corresponding authors.

Research involving human participants, their data, or biological material

Policy information about studies with human participants or human data. See also policy information about sex, gender (identity/presentation), and sexual orientation and race, ethnicity and racism.

Reporting on sex and gender	Details on the sex and/or gender of human cell lines are outlined below in "Eukaryotic Cell Lines".
Reporting on race, ethnicity, or other socially relevant groupings	Where known, details on the ethnicity of human cell lines are outlined below in "Eukaryotic Cell Lines"
Population characteristics	N/A
Recruitment	N/A
Ethics oversight	All human stem cell work was approved by Royal Children's Hospital Research Ethics Committee (HREC) 33001A and 93025.

Note that full information on the approval of the study protocol must also be provided in the manuscript.

Field-specific reporting

Please select the one below that is the best fit for your research. If you are not sure, read the appropriate sections before making your selection.

☒ Life sciences ☐ Behavioural & social sciences ☐ Ecological, evolutionary & environmental sciences

For a reference copy of the document with all sections, see nature.com/documents/nr-reporting-summary-flat.pdf

Life sciences study design

All studies must disclose on these points even when the disclosure is negative.

Sample size	Based on our previous experience in screening with organoids (Mills et al. Cell Stem Cell, 2019; Mills et al. Cell 2021, Pocock et al. Nature Cardiovascular Research 2025), n=6-8 organoids are required to differentiate a functional differences >10%. Then experiments are repeated multiple times to ensure reproducibility. For animal studies, no sample size calculation was performed prior to experiments.
Data exclusions	No data were excluded from this study
Replication	The results depicted in this manuscript are representative of observations and analyses made across multiple experiments, biological replicates, and technical replicates. Each experiment included a minimum of 3 biological replicates per condition. All replication attempts were successful.
Randomization	All animal and organoids were randomised to treatment arm.
Blinding	All animal experiments were performed with the operator blinded to treatment regime.

Reporting for specific materials, systems and methods

We require information from authors about some types of materials, experimental systems and methods used in many studies. Here, indicate whether each material, system or method listed is relevant to your study. If you are not sure if a list item applies to your research, read the appropriate section before selecting a response.

Materials & experimental systems

n/a	Involved in the study
☐	☒ Antibodies
☐	☒ Eukaryotic cell lines
☒	☐ Palaeontology and archaeology
☐	☒ Animals and other organisms
☒	☐ Clinical data
☒	☐ Dual use research of concern
☒	☐ Plants

Methods

n/a	Involved in the study
☒	☐ ChIP-seq
☒	☐ Flow cytometry
☒	☐ MRI-based neuroimaging

Antibodies

Antibodies used	Mouse IgG1 Anti-Sarcomeric Alpha Actinin. Sigma-Aldrich A7811 (1:200)<br>Rabbit IgG Anti-FLAG. Cell Signalling Technologies 14793 (1:200)<br>Wheat Germ Agglutinin, 647 Conjugated. Invitrogen W32466 (1:2000)<br>Wheat Germ Agglutinin, 488 Conjugated. Invitrogen W11261 (1:2000)<br>Mouse IgG1 Anti-Cardiac Troponin-T. Abcam ab8295 (1:200)<br>Mouse IgG1 Anti-FLAG Sigma-Aldrich F9291 (1:200)<br>Rabbit IgG Anti Sarcomeric Alpha Actinin 2. Abcam ab121652 (1:500)<br>Rabbit IgG Anti-Obscurin. Abcam ab121652 (1:500)<br>Rabbit IgG Anti-MYOM1. Abcam ab205618 (1:500)<br>Goat anti-mouse IgG (H+L), Alexa Fluor 647. Invitrogen A21236 (1:400)<br>Goat anti rabbit IgG (H+L), Alexa Fluor 488. Invitrogen A11034 (1:400)
Validation	Antibodies were previously validated for immunofluorescence of human iPSCs or mouse cardiac tissues.

Eukaryotic cell lines

Policy information about cell lines and Sex and Gender in Research

Cell line source(s)	ALPK3 mutant lines were derivatives of the female HES3 NKX-GFP reporter cell line (Elliott et al. Nature Methods 2011 Oct 23;8(12)). The HES3 cell line is available from WiCell (ES03), and was originally reported in Richards et al. Nature Biotechnology 2002 Sep;20(9):933-6. The ALPK3 mutant line has been described in McNamara et al. Nature Cardiovascular Research 2023 Feb; 2: 159-173.<br><br>The TTN tuncating variant line was generated at MCRI by introducing the relevant variant (c.) into a male iPSC line MCRIi010-A-1 that was generated from blood mononuclear cells from a healthy 22 year old caucasian male. The generation of this parental line has been described in Vlahos et al. Stem Cell Research 2019 Jan; 34 :101380.
Authentication	hPSC lines are checked for pluripotency and genomic integrity by immunofluorescence of pluripotency markers and molecular karyotyping
Mycoplasma contamination	All cell lines confirmed negative for mycoplasma.
Commonly misidentified lines (See ICLAC register)	No commonly misidentified cell lines were used.

Animals and other research organisms

Policy information about studies involving animals; ARRIVE guidelines recommended for reporting animal research, and Sex and Gender in Research

Laboratory animals	C57BL/6J mice were used in this study. Both wild-type mice and littermates carrying a homozygous truncating variant in the Alpk3 gene (Alpk3-W1538X). Mice were fed standard chow and water ad libitum, and maintained in a 12-hour light/12-hour dark cycle at ambient room temperature. All animal experiments were performed with the operator blinded to treatment regime.
Wild animals	No wild animals were used in this study
Reporting on sex	All experiments were performed in mixed sex.<br>Neonatal study:<br>WT n = 17 (9M, 8F)

Hom n = 19 (8M 11F)

Adult Study:
WT n=8 (4M, 4F)
Hom n =17 (8M, 9F)

Field-collected samples	No field collected samples were used in this study.
Ethics oversight	All experiments were approved by the Animal Ethics Committee of the Murdoch Children's Research Institute, Parkville, Australia

Note that full information on the approval of the study protocol must also be provided in the manuscript.

Plants

Seed stocks	N/A
Novel plant genotypes	N/A
Authentication	N/A
