# Supplementary material for: Alpha protein kinase 3 gene therapy restores heart function in mouse and human models of cardiomyopathy
Source: Nat Cardiovasc Res. 2026 Aug 19;5(9):795–806. doi: 10.1038/s44161-026-00843-1 (PMC13561881; doi:10.1038/s44161-026-00843-1)
Supplement: Supplementary file 2 [file 44161_2026_843_MOESM2_ESM.pdf]

nature portfolio

Peer Review File

Alpha Protein Kinase 3 Gene Therapy Restores Heart Function in Mouse and Human Models of Cardiomyopathy

Corresponding Author: Dr James McNamara

This file contains all reviewer reports in order by version, followed by all author rebuttals in order by version.

Version 0:

Reviewer comments:

Reviewer #1

(Remarks to the Author)
This study by McNamara focusses on AAV-mediated gene replacement therapy to deliver full-length human Alpha Kinase 3 (ALPK3) in a mouse model bearing the human equivalent (AlpkW1538X) of the pathological mutation (AlpkW1765X). In doing so the authors were able to show that gene therapy reverses cardiomyopathy in mice, by reducing cardiac dysfunction and remodeling. The authors further show that replacement of human ALPK3 in human cardiac organoid models, harbouring either ALPK truncating variants or titin truncating variants, restored contractile function.

This is a timely and solid study, performed in human relevant disease models showing the potential impact of gene replacement therapy. There are however several issues that remain to be answered:
- The authors should demonstrate the level of ALPK3 upregulation in the mouse studies compared to control mice after gene therapy.
- It is important to show the localization of the exogenously expressed ALPK3 protein in the mouse studies?
- Did the supplemented ALPK3 protein localize at the M-band of the sarcomere and co-localization with known interacting proteins?
- The authors hypothesize that restoration of the sarcomeric structure as a main reason for the rescue effect, however, only stainings from the 2D-iPSC-CMs was shown and there were no immunohistochemistry stainings to show that this is also the case in vivo.
- This study requires a more thorough molecular phenotyping. Does the gene therapy also restore the molecular phenotype of the mice? Is there a regulation of stress markers that is reversed by the gene therapy. What are the phosphorylation targets of ALPK3 and are these rephosphorylated after gene therapy?
- The authors mention that ALPK3 coordinates a protein quality control hub to maintain contractile protein turnover. The authors should check downstream contractile protein targets regulated through this mechanism that show rescue through AAV-humanALPK3 therapy.
- The authors should try to determine the amount of cells that were targeted after gene therapy by staining for human ALPK3.
- Did the authors check the skeletal muscle and liver for expression of human ALPK3?
- Related to Figure 3B, is there a staining for the WT control? This is vital to show that the ALPK3 level after the gene therapy is back to WT levels.

Minor points:
• Is there a reason why the control group in the adult mice study is 'untreated' in WT and not AAV-control (used in neonatal study)?
• Data referral to 'extended data 1d-g' but extended data only goes to 'f'.
• Different fonts in different panels of the same figure.
• Is there a rationale for why the neonates were treated with a viral load of 2x1014 vg/kg but in adult mice 1x1014 vg/kg?
• The labelling in general is very inconsistent in the figures.
• Wrong figure call-out throughout the text, please check.
• Figure 2, missing the statistical comparison labels.
• In figure 3, authors do not specify which ALPK3 mutated cell line was used.
• For figure 3f, the authors do not refer where the single-cell data for the pseudobulk analysis came from.
• In Extended Data Figure 1b, is the 'AAV6-Empty' label in the graph a typo as all the other groups are labelled with 'AAV9'?

Reviewer #2

(Remarks to the Author)
SUMMARY

McNamara et al. propose an AAV-mediated full-length ALPK3 gene-addition strategy as therapy for ALPK3 truncation-associated cardiomyopathy, and extend the concept to TTN truncation cardiomyopathy via a shared proteostasis rationale. They report prevention of disease when dosing neonatal Alpk3 mutant mice, reversal of established disease after adult dosing, and functional rescue in human ALPK3-mutant cardiomyocytes/organoids as well as improved force generation in TTNtv organoids.

ASSESSMENT

The study presents potentially impactful results, particularly the apparent reversal of established cardiomyopathy following adult gene delivery. I think the proof of both prevention and "rescue" of established disease in the ALPK3 mouse model is very exciting. I also particularly appreciate the "epistasis" narrative using gene addition of ALPK3 to rescue a pathophysiology caused by mutation in another gene (TTN).
However, the manuscript requires some more experimental work, mechanistic depth, and controls. I have concerns regarding the AAV strategy and wonder why this approach would be more clinically meaningful than the miniALPK3 approach? Addressing these issues would substantially strengthen confidence in the conclusions and translational relevance.

MAJOR COMMENTS

1. From an AAV/packaging capacity (4.7kb) point of view, I am wondering how the authors' approach compares to the recently published miniALPK3 approach by Feng et al. https://pmc.ncbi.nlm.nih.gov/articles/PMC12626394/

ALPK3 encodes a 1705AA (~5.1 kb) CDS (without any regulatory elements) which is far above the classical AAV packaging limit (~4.7 kb). This is exactly why others went the "miniALPK3" route. The manuscript states that "full-length human ALPK3" was delivered by AAV9 under a TNNT2 promoter but does not provide a schematic of the expression cassette (promoter, WPRE, polyA, any introns, tags).

The authors should address that they are using an "oversized AAV" strategy and whether they see concatemer formation or truncated genomes on Southern blot/qPCR.

I don't mean this in a "novelty" sense re miniALPK3 but rather in a technical sense.
The authors should also show:
- a vector map and total ITR-to-ITR size.
- data confirming that the predominant expressed product in vivo is truly full-length ALPK3 (e.g. Western blot from mouse hearts, not only FLAG-IF in monolayers)
- at least a short discussion on how they see this vector being advanced clinically, given packaging limits and the competing miniALPK3 concept

It would be best to compare both systems (mini vs. full-length) side-by-side in vivo using the same AAV9 and promoter architecture.

2. The paper infers proteostasis restoration at the sarcomeric M-band to explain efficacy across ALPK3-deficient and TTN-truncation models but does not measure established proteostasis endpoints (LC3 flux, p62, ubiquitin conjugates, proteasome activity) in Figures 1–3. Absent these pathway-level readouts, the causal link between ALPK3 transduction and proteostasis normalization remains unproven, weakening rationale for indication expansion. What if ALPK3 overexpression has a completely different (beneficial) effect?

3. Vector dose, cardiac vector genomes, APLK3 mRNA/protein expression levels, and safety need more rigorous characterization.
The study uses relatively high systemic AAV doses, particularly in neonatal mice. Beyond gross measures such as body and spleen weight, additional data are needed on cardiac vector genome copy number, expression relative to endogenous ALPK3, biodistribution, and markers of cardiac and hepatic toxicity. Inclusion of WT animals treated with AAV-ALPK3 would be critical to define the consequences of supraphysiologic ALPK3 expression.
Of note, without measuring cardiac ALPK3 mRNA/protein levels and vector genome copies in the heart, it is not possible to causally associate the improvements shown in Figures 2c–2g with cardiomyocyte transduction from the timeline in Figure 2a.

4. Adult disease "reversal" requires additional endpoints.
While echocardiographic normalization after adult dosing is compelling, claims of disease reversal should be supported by histological analysis (fibrosis, cardiomyocyte size, sarcomeric organization), arrhythmia assessment, and/or potentially also longer-term durability of rescue.

5. Human ALPK3 models require clearer definition and controls.

The precise ALPK3 variant(s), zygosity, and baseline phenotypes of the human cardiomyocytes/organoids should be explicitly stated. In addition, the observed increase in force with AAV-control treatment (Fig. 3c) require a control with WT organoids with vs. without vector treatment as well as a kinase-dead ALPK3 control.

6. The TTN cardiomyopathy extension is currently somewhat underpowered.
The TTNtv data are limited to expression correlations and force rescue in organoids. Stronger mechanistic support is needed to justify broader claims, including direct readouts of proteostasis, sarcomere integrity etc.

MINOR COMMENTS

7. Model-to-patient alignment should be discussed more carefully.
The use of a homozygous truncation mouse model with early and severe disease contrasts with the typically dominant, later-onset human ALPK3 cardiomyopathy. The authors should discuss this discrepancy explicitly and clarify how well the current models reflect the intended clinical population.

8. The authors should consider deposition of key datasets rather than data availability upon request.

Reviewer #3

(Remarks to the Author)
In this manuscript, McNamara et. al. demonstrated overexpression of Alpk3 delivered by AAV9 with cardiomyocyte specific promoter in Alpk3W1538X mice, a model of cardiomyopathy in loss of function manner, prevent and reverse cardiomyopathy. Futhermore, they showed AAV-Alpk3 restored cardiac organoids contraction in TTNtv variant.

This study is very interesting, and suggests overexpression of Alpk3 can be a novel approach for the treatment of TTNtv variant, the most common cause of genetic DCM. Overall, it is well written manuscript, however, I have several concerns.

In page 3, 3rd paragraph, they stated “Homozygous mice are born with enlarged hearts”, but this is not described in the original paper reporting this mouse model. Please provide the evidence of neonatal cardiac enlargement.

They use ejection fraction as a parameter of cardiac systolic function but since mouse heart rate is very fast, it is more appropriate to use fractional shortening avoiding potential error inflation.

Since this is a mouse model of HCM AND DCM, they should present LVDd in main figure.

They use IVRT as a parameter of diastolic function which I agree that it is quite reliable method, however, since IVRT is a duration that was affected strongly by HR, and again, mouse HR is very high and varies, I would recommend to use IVRT adjusted to heart rate (normalized IVRT).

Extended data 1b was not appeared in the text body. Why wt is with AAV6 not AAV9?

In page 4, 1st paragraph, they claim there are no sign of off-target organ pathologies. Since the virus titer was relatively high which is ~2x1014 vg/kg, and hepatotoxicity is one of the most common side effects, please confirm that there are no findings to suggest hepatotoxicity.

Reversed HCM AND DCM phenotype in adult mice by AAV9-Tnnt2-Alpk3 was determined based on echo data. LVPW reduced its thickness by approximately 0.2mm in only 2 weeks in adult treatment group which is a relatively acute change. Are there any histological changes, ie fibrosis status? Do they reverse fibrosis as well? Please provide histological assessment.

They performed single nucRNA sequencing of TTNtv/tv, TTNtv/+ and TTN+/+ only to prove relative ALPK3 expression level alters by TTN gene dosage. They should provide more intensive analysis to examine ie other gene expression in the complex at M-band. The mechanism of how TTNtv variant cause decrement of ALPK3 expression is not clear. It would be beneficial to have mechanistic insights to understand how ALPK3 expression decreases and hence the ALPK3 supplement improve CM contractile function in TTNtv variant.

They should describe more detailed methods such as echocardiography or functional assessment of organoid. How they identify arrhythmogenicity of organoid? Even though they have used the same protocol as previously described elsewhere, brief description should be provided.

There is a typo in mouse description in method section. C57/BL6J should be C57BL/6J.

Version 1:

Reviewer comments:

Reviewer #1

(Remarks to the Author)
This manuscript presents convincing and solid data regarding the applicability of using Alpk3 gene therapy for cardiomyopathy. The data support the conclusions and provide confidence that this approach could become of potential clinical benefit. This is especially timely given that several studies using gene therapies are starting to show positive outcomes in patients suffering from different forms of cardiomyopathy.

Reviewer #2

(Remarks to the Author)
Thanks a lot for addressing all of my concerns.
I think the manuscript is in great shape now.
Congrats!

Two minor follow-up comments:

1.
The claim that the mini-version lacks "functionally important domains" rests on a cross-study comparison with different capsids and routes. I would soften that wording in the discussion.

2.
Could the authors potentially provide any kind of quantification for the sarcomere claim in Fig. 15e?

Reviewer #3

(Remarks to the Author)
I have three minor concerns.
#1, The quantification of cell size were presented in Fig. 2f, Extended Data Fig. 1c, d. It is not clearly stated how they collected the data. Since the cardiomyocyte area measurement depends on the cell alignment, ie cross section over longitudinal, they should collect multiple different layers from each heart, from basal to the apex. What is their definition of cross section?
#2, Do the authors have any thoughts why they have not observed any fibrosis in the Alpk3W1538X model? ALPK3 cardiomyopathy is known to have extensive fibrosis. They have performed WGA staining, but how about Masson’s trichrome or Sirius Red staining? Did snRNAseq data present any changes in collagen or other fibrosis related genes? Fibrosis reduction/regression should be one of the key components in terms of the ALPK3 cardiomyopathy treatment.
#3, In echocardiography method, please clarify at what HR they collected the data. Cardiac function is altered by HR. Bradycardia causes reduced FS even in WT mice. They have changed EF to FS in the figures, but there are several sentences still stating ejection fraction in main body.

Version 2:

Reviewer comments:

Reviewer #3

(Remarks to the Author)
The authors answered all my questions, and I have no further comments/concerns.

Open Access This Peer Review File is licensed under a Creative Commons Attribution 4.0 International License, which permits use, sharing, adaptation, distribution and reproduction in any medium or format, as long as you give appropriate credit to the original author(s) and the source, provide a link to the Creative Commons license, and indicate if changes were made.
In cases where reviewers are anonymous, credit should be given to 'Anonymous Referee' and the source.
The images or other third party material in this Peer Review File are included in the article's Creative Commons license, unless indicated otherwise in a credit line to the material. If material is not included in the article's Creative Commons license and your intended use is not permitted by statutory regulation or exceeds the permitted use, you will need to obtain permission directly from the copyright holder.
To view a copy of this license, visit https://creativecommons.org/licenses/by/4.0/

Summary.

We thank the reviewers for the time and effort taken to review our manuscript entitled "***Alpha Protein Kinase 3 Gene Therapy Restores Heart Function in Mouse and Human Models of Cardiomyopathy***". We appreciate the constructive and positive feedback and have gone to considerable efforts to address the concerns raised. We agree with the broad assessment of the reviewers regarding the potential impact of such a gene therapy and have considerably revised our manuscript in response to their thoughtful suggestions. As a result, we believe the manuscript is much stronger. The major changes included in the revised manuscript are as follows:

1. Delivery of AAV9-TNNT2-*ALPK3* to adult *Alpk3*^{W1538X} mice ***with established cardiomyopathy*** almost completely reverses the disease following 12 weeks of treatment.
2. In-depth molecular profiling of the response of adult *Alpk3*^{W1538X} mice to AAV9-TNNT2-*ALPK3* treatment, including transcriptomics, proteomics, and phosphoproteomics. These rich datasets reveal that 95% proteomic, and 98% of phosphoproteomic, disease signature is reversed by AAV mediated ALPK3 expression. Notably, we observe that pT269/S272 Sqstm1, previously identified as a possible target of Alpk3, is returned to wild-type levels in mutant mice upon delivery of AAV9-TNNT2-*ALPK3.* Thus, this comprehensive molecular phenotyping is consistent with restoration of normal heart function observed in *Alpk3*^{W1538X} adult mice treated with AAV9-TNNT2-*ALPK3*.
3. Mechanistic insights into the potential of AAV9-TNNT2-*ALPK3* to improve contractility in cardiomyopathy arising from *TTN* truncating variants. Proteomic analysis of single cardiac organoids from *TTN*$^{tv/+}$ cardiac organoids treated AAV6-*ALPK3* revealed that ALPK3 restored cellular proteostasis. Furthermore, combining mouse and human proteomics data identified a core ALPK3-regulated set of proteins that demonstrate that regulation of cellular proteostasis is the primary mode of action of *ALPK3* gene therapy.
4. To identify genes that exacerbate the ALPK3 phenotype we performed unbiased in silico analysis using the Geneformer transfer learning model which showed that TTN loss resulted in the largest disruption of the cardiomyocyte transcriptome. This provides orthogonal support for our observation that ALPK3 overexpression compensates for impaired sarcomere function in *TTN* cardiomyopathy.
5. Demonstration that delivery of AAV9-TNNT2-*ALPK3* at clinically high doses (1-2x10^{14} vg/kg) does not impart liver stress as measured by DNA/RNA copy number, serum measures of liver damage (ALT/AST) or histology.

Below we address each issue raised by the reviewers (Blue text) in detail. In the accompanying manuscript changes to the text are highlighted in blue text to assist in the review process. We trust the reviewers agree that the revised manuscript includes a comprehensive dataset that addresses the major issues raised and, as a result, is substantially improved.

Reviewer #1 (Remarks to the Author):

1. The authors should demonstrate the level of ALPK3 upregulation in the mouse studies compared to control mice after gene therapy.

This is an important point. To address this, we have performed qPCR, RNAseq, and proteomics across cardiac tissues in both neonatal and adult studies.

First, qPCR analysis demonstrated a significant increase in human *ALPK3* (hs*ALPK3*) transcript levels in mice treated at P0 **(Response Figure 1a;** *in manuscript* **Figure 1i**). Delivery of AAV9-TNNT2-*ALPK3* in adult mice similarly resulted in robust expression of hs*ALPK3* transgene, corresponding to approximately $3.6x10^7$ copies per μg RNA (**Response Figure 1b, c;** *in manuscript* **Figure 3a).**

RNAseq analysis of the same adult-treated mice further confirmed strong transgene expression, with hs*ALPK3* detected at ~250 counts per million (CPM) compared with ~30 CPM for ms*Alpk3* in wild-type mice (**Response Figure 1d and e;** *in manuscript* **Extended Data Figure 5a and b**). These data demonstrate robust hs*ALPK3* transcript expression following AAV9-TNNT2-*ALPK3* delivery.

To determine whether transcriptional upregulation translated to protein expression, we performed quantitative proteomics on cardiac tissue isolated from the adult-treated group. Using this dataset, we analysed human-specific ALPK3 peptides within $Alpk3^{KI/KI}$ AAV9-TNNT2-*ALPK3* group. Human ALPK3 peptides were detected across the length of the protein with the exception of two regions due to the technical challenge of those regions possessing fewer tryptic peptides (**Response Figure 1f;** *in manuscript* **Extended Data Figure 5c**; detected peptides presented in blue, with predicted tryptic peptides in purple), confirming expression of the full-length transgene product. Importantly, quantitative analysis showed that human ALPK3 protein levels were equivalent to, or higher than, endogenous mouse Alpk3 in wild-type hearts, as measured by log_{10}iBAQ intensity (**Response Figure 1g;** *in manuscript* **Extended Data Figure 5d**)**.**Collectively, these data demonstrate robust expression of human ALPK3 in mouse myocardium following AAV9-TNNT2-*ALPK3* delivery, reaching levels comparable to or modestly exceeding endogenous msAlpk3.

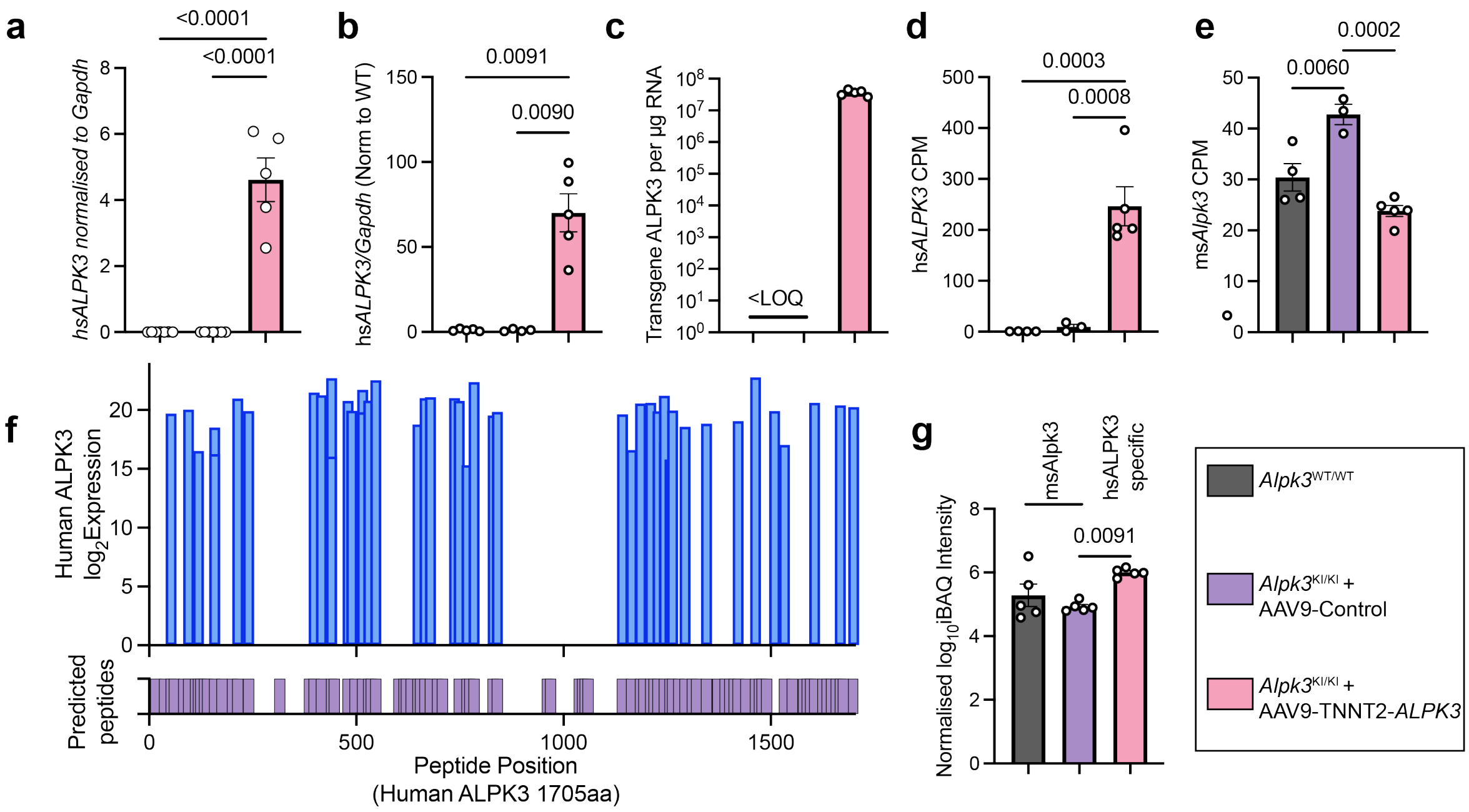

Response Figure 1. Upregulation of hsALPK3 in both neonatal and adult study groups. **a.** qPCR expression of hsALPK3 across WT-AAV9-Control, *Alpk3*$^{KI/KI}$-AAV9-Control and *Alpk3*$^{KI/KI}$-AAV9-TNNT2-*ALPK3* mice treated at P0 and analysed at P84; and **b.** qPCR expression of hsALPK3 in mice treated at P42 and P126 both show robust upregulation of hs*ALPK3*. **c.** Copy number analysis by qPCR using a standard curve demonstrate ~3.6x10^7 copies of hsALPK3 per microgram RNA. **d.** RNAseq from adult-treated mice show ~250 counts per million of hs*ALPK3*. **e.** Similar analysis of ms*Alpk3* demonstrate normalisation of ms*ALPK3* in *Alpk3*$^{KI/KI}$-AAV9-TNNT2-*ALPK3* back to WT levels. **f.** Analysis of human specific ALPK3 peptides in *Alpk3*$^{KI/KI}$-AAV9-TNNT2-*ALPK3* hearts shows consistent expression across the transgene with predicted tryptic peptides presented below in purple. **g.** iBAQ abundance analysis demonstrates hsALPK3 protein expression equivalent or slightly higher in *Alpk3*$^{KI/KI}$-AAV9-TNNT2-*ALPK3* than mouse *Alpk3* in WT-AAV9-Control and *Alpk3*$^{KI/KI}$-AAV9-Control.

2. It is important to show the localization of the exogenously expressed ALPK3 protein in the mouse studies?
and
3. Did the supplemented ALPK3 protein localize at the M-band of the sarcomere and co-localization with known interacting proteins?

We agree with the reviewer that demonstrating correct localisation of the exogenously expressed ALPK3 protein in heart tissue is important. To assess this, we performed immunofluorescence labelling of *Alpk3*$^{KI/KI}$ mouse hearts treated with AAV9-CMV-ALPK3 containing a C-terminal FLAG epitope tag.

This strategy was necessary because, despite extensive testing of both commercially available and custom-made antibodies, we have been unable to validate a specific ALPK3 antibody. We have to date tested 8 commercially available ALPK3 antibodies using our ALPK3 mutant cell lines as controls for specificity. However, **none** of these commercial antibodies specifically detected ALPK3. Furthermore, we have tried to generate our own primary antibodies (7 monoclonal, 3 polyclonal, and 3 alpaca-derived nanobodies) against ALPK3 using multiple commercial suppliers, but we have been unable to generate a specific antibody. The use of a FLAG tag therefore provides a reliable method to detect the transgenic protein while also providing specificity for the human transgene over the endogenous mouse Alpk3.

Cardiac sections were labelled with a mouse IgG1 anti-FLAG antibody (Sigma F1804) and either a rabbit ACTN2 (abcam ab68167) or OBSCN (ab121652), which mark the sarcomeric Z-disc or M-band, respectively. Consistent with our *in vitro* experiments, transgenic flag-tagged hsALPK3 demonstrated the characteristic interdigitation between FLAG and the Z-disc ACTN2 (**Response Figure 2a,b;** *in manuscript* **Extended Data Figure 3a, c)**, and co-localisation with the M-Band protein OBSCN (**Response Figure 2c,d;** *in manuscript* **Extended Data Figure 3b,c).** These findings are also consistent with recent observations reported by Feng et al. (2025, *Circulation*) who observed similar M-band localisation using a mini-ALPK3 construct. Thus, AAV expressed hsALPK3 correctly localised to the M-band of the sarcomere *in vivo*.

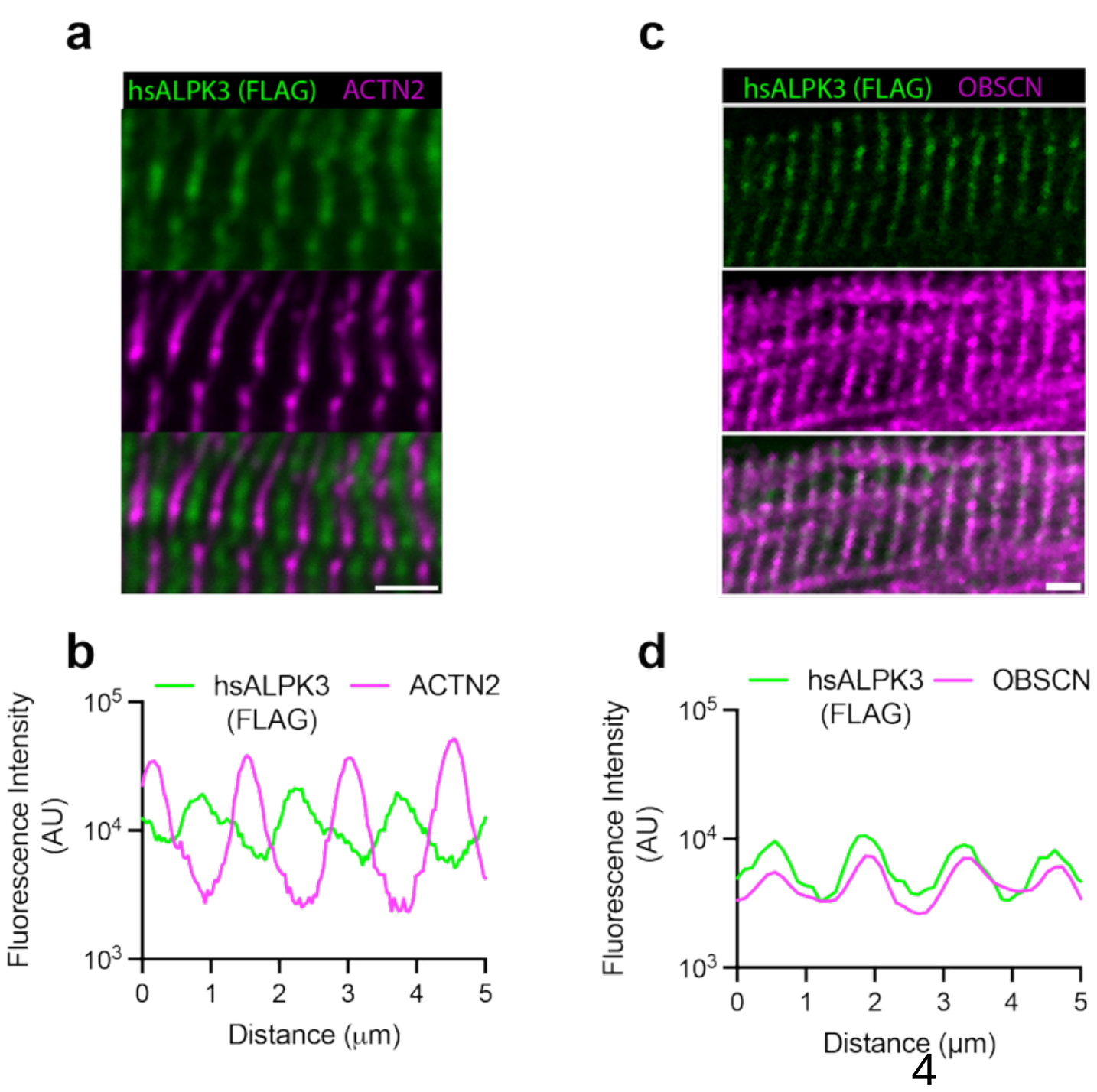

Response Figure 2. AAV9-expressed ALPK3 correctly localises to the cardiac M-band. **a.** Immunofluorescence labelling of *Alpk3*$^{KI/KI}$ treated with AAV9-CMV-ALPK3-FLAG demonstrate interdigitation of ACTN2 and FLAG, consistent with M-band localisation of ALPK3. **b.** Representative line profile demonstrating interdigitation of ACTN2 and FLAG. **c and d.** Co-localisation of M-band marker OBSCN and FLAG in the same mice further supports M-band localisation of transgenic ALPK3. Scale bar = 2μm.

4. The authors hypothesize that restoration of the sarcomeric structure as a main reason for the rescue effect, however, only staining from the 2D-iPSC-CMs was shown and there were no immunohistochemistry staining to show that this is also the case in vivo.

To address this point, we have performed immunofluorescent staining of cardiac tissue from both neonatal-treated mice (P0 injection; analysed at P82) and adult-treated mice (P42 injection; analysed at P126) to assess sarcomeric structure.

Sections were stained for ACTN2 and MYOM1, which mark the sarcomeric Z-disc and M-band, respectively. In neonatal treated mice, *Alpk3*$^{KI/KI}$ AAV9-Control mice displayed marked sarcomeric disorganisation compared with *Alpk3*$^{WT/WT}$ mice. This defect was particularly evident in the disruption of MYOM1 M-band structure, consistent with previous observations in this model (McNamara et al., 2023, *Nature Cardiovascular Research* ***Extended Data Figure 4d***).

In contrast, treatment of *Alpk3*$^{KI/KI}$ mice with 2x10^{14} vg/kg of AAV9-TNNT2-*ALPK3* largely restored sarcomeric organisation, with re-establishment of the characteristic interdigitation of ACTN2 and MYOM1 (**Response Figure 3a;** *in manuscript* **Extended Data Figure 3d)**. These data demonstrate that AAV9-mediated expression of hsALPK3 could restore pre-existing sarcomeric disarray, which in this model is evident as early as P21 (McNamara et al.,2023, *Nature Cardiovascular Research*).

To further assess structural remodelling, we stained heart sections from the adult-treated cohort with wheat germ agglutinin (WGA) to assess cardiomyocyte cross sectional area. This analysis showed that cardiomyocyte hypertrophy observed in the *Alpk3*$^{KI/KI}$ mice was also reversed following AAV9-TNNT2-*ALPK3* treatment (**Response Figure 3b,c;** *in manuscript* **Figure 2f**).

From these data, it is clear that *in vivo* expression of hsALPK3 restores sarcomeric organisation and reverses cardiomyocyte hypertrophy, supporting the therapeutic benefit of this novel gene therapy.

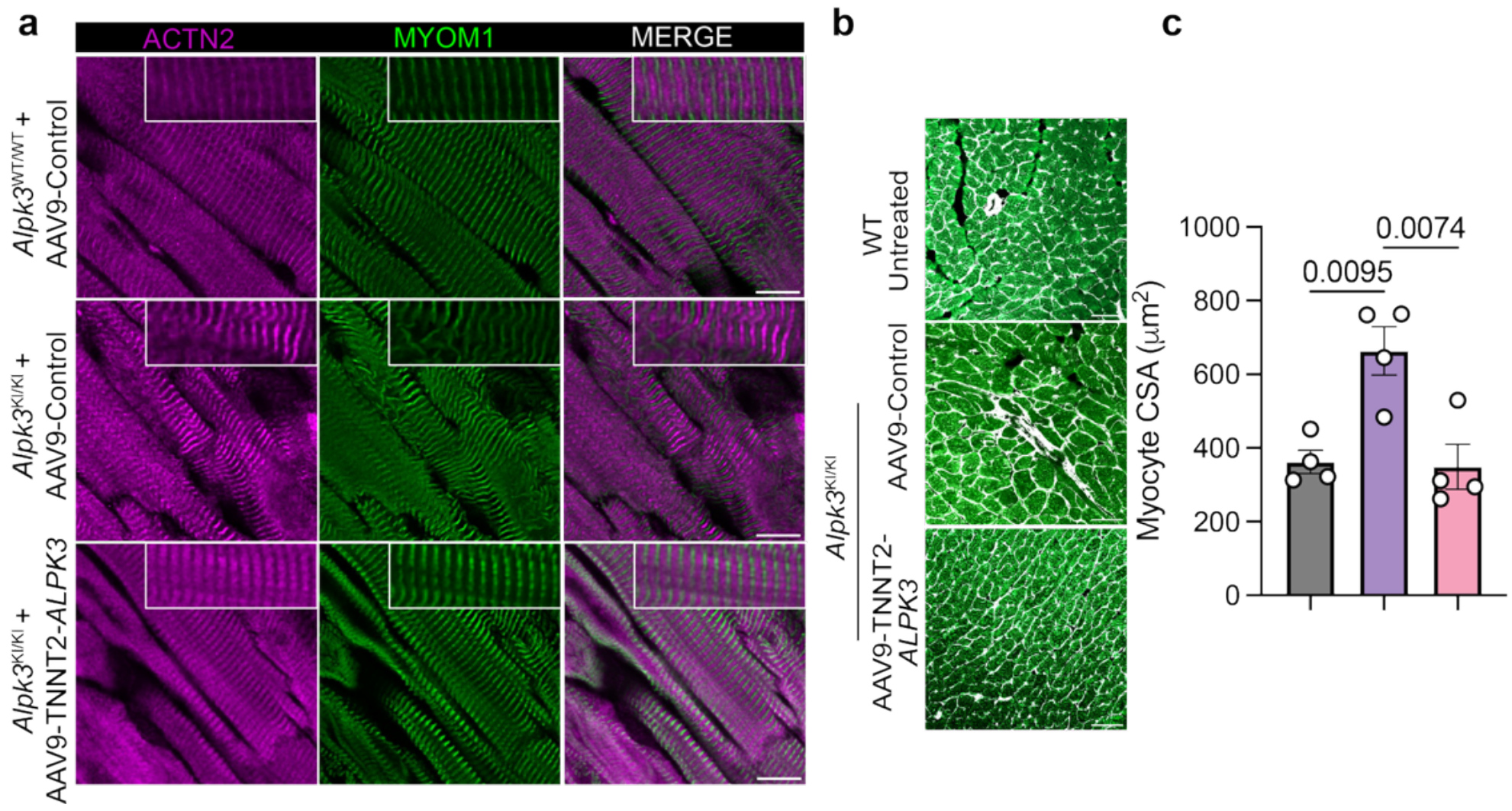

***Response Figure* 3. Restoration of cardiac structure following *ALPK3* gene therapy treatment. a.** Left ventricular (LV) tissue from neonatal study mice stained with Z-disk marker ACTN2 and M-band marker MYOM1 shows sarcomeric disorganisation evident from the abnormal banding pattern in *Alpk3*$^{KI/KI}$ AAV9-Control, which is restored in *Alpk3*$^{KI/KI}$ mice treated with AAV9-TNNT2-*ALPK3*. **b.** LV tissue from adult study stained with wheat germ agglutinin demonstrates reversal of myocyte hypertrophy in *Alpk3*$^{KI/KI}$ mice treated with AAV9-TNNT2-*ALPK3*. **c.** Quantification of cross-sectional area (CSA) of cardiac myocytes. Data show the mean of ~100 CMs from each mouse (n=4), error bars show SEM.

5. This study requires a more thorough molecular phenotyping. Does the gene therapy also restore the molecular phenotype of the mice? Is there a regulation of stress markers that is reversed by the gene therapy. What are the phosphorylation targets of ALPK3 and are these rephosphorylated after gene therapy?

Previously, we showed that treatment of P0 *Alpk3*$^{KI/KI}$ pups with AAV9-TNNT2-*ALPK3* restored both *Myh7* and *Nppa* to *Alpk3*$^{WT/WT}$ levels (**Response Figure 4a and b;** *in manuscript* **Figure 1i and j**). We have now extended this analysis to adult-treated mice, demonstrating that the cardiac stress markers *Myh7*, *Nppa*, and *Nppb* in *Alpk3*$^{KI/KI}$ mice are all significantly reduced back to WT levels with treatment of AAV9-TNNT2-*ALPK3* (**Response Figure 4c-e;** *in manuscript* **Figure 3b-d).**

To assess network-wide disease reversal we performed quantitative proteomics and phosphoproteomics on heart tissue from *Alpk3*$^{WT/WT}$, *Alpk3*$^{KI/KI}$ AAV9-Control and *Alpk3*$^{KI/KI}$ AAV9-TNNT2-ALPK3 groups from the adult-treated cohort. These were performed at depth, detecting 6022 proteins and 19759 phosphopeptides (from 4109 proteins). Principal component analysis revealed tight clustering of *Alpk3*$^{WT/WT}$ and *Alpk3*$^{KI/KI}$ AAV9-TNNT2-ALPK3 relative to *Alpk3*$^{KI/KI}$ AAV9-Control (**Response Figure 4f;** *in manuscript* **Figure 3e**). While there were 1612 differentially expressed proteins between *Alpk3*$^{WT/WT}$ and *Alpk3*$^{KI/KI}$ AAV9-Control (**Response Figure 4g;** *in manuscript* **Figure 3f**), only 83 remained dysregulated between *Alpk3*$^{WT/WT}$ and *Alpk3*$^{KI/KI}$ AAV9-TNNT2-ALPK3 (**Response Figure 4h,** *in manuscript* **Figure 3i**). This striking finding shows that 95% dysregulated proteins are restored to wildtype levels by AAV9-TNNT2-ALPK3 transduction, which clearly demonstrates a marked reversal of the molecular phenotype. This data is presented in full as **Figure 3** of the revised manuscript.

Furthermore, phosphoproteomic analysis revealed 1004 differentially phosphorylated peptides (from 417 proteins) between *Alpk3*$^{WT/WT}$ and *Alpk3*$^{KI/KI}$ AAV9-Control (**Response Figure 4i;** *in manuscript* **Extended Data Figure 7a-c**). This was dramatically reduced to just 27 peptides (from 20 proteins) between *Alpk3*$^{WT/WT}$ and *Alpk3*$^{KI/KI}$ AAV9-TNNT2-ALPK3 – representing a 98% restoration of the phosphoproteome towards WT (**Response Figure 4j;** *in manuscript* **Extended Data Figure 7d**).

Regarding ALPK3 phosphorylation targets, we note that whether ALPK3 functions as an active or pseudokinase remains an open question (PMIDs: 39196058, 36321451, 38048395). Clarifying this biology is outside the scope of the current manuscript. However, leveraging our phosphoproteomic dataset, we examined the previously reported potential ALPK3 dependant phosphosites, T269/S272 of SQSTM1 (McNamara et al., 2023, *Nature Cardiovascular Research*). pT269/S272 Sqstm1, normalised to total Sqstm1, was reduced in *Alpk3*$^{KI/KI}$ AAV9-Control hearts compared to *Alpk3*$^{WT/WT}$ and was restored to wildtype levels in the *Alpk3*$^{KI/KI}$ AAV9-TNNT2-ALPK3 group (**Response Figure 4k;** *in manuscript* **Extended Data Figure 7e**). We note that this finding is consistent with, but does not in itself prove, direct kinase activity as secondary effects cannot be excluded.

Taken collectively, these multiomic datasets provide a thorough molecular phenotype and demonstrate a near complete restoration of the molecular signature of disease following hsALPK3 gene therapy in this mouse model.

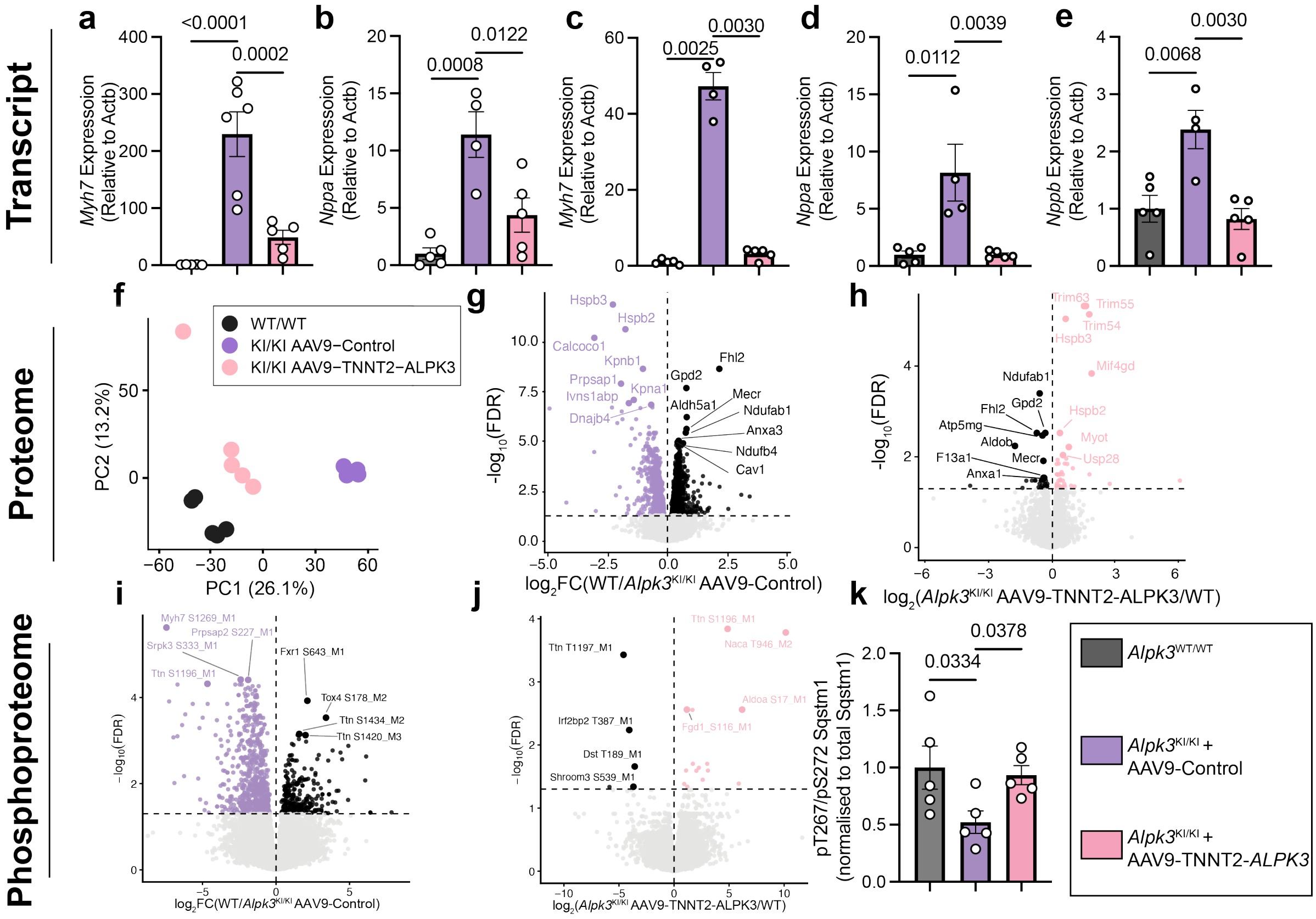

Response Figure 4. Restoration of molecular signatures of cardiomyopathy in Alpk3$^{KI/KI}$ AAV9-TNNT2-ALPK3 treated mice. **a.** qPCR expression of *Myh7*, and **b.** *Nppa* in mice treated at P0 and analysed at P84 shows reduction in cardiac hypertrophy markers in *Alpk3*$^{KI/KI}$ AAV9-TNNT2-*ALPK3* . qPCR expression analysis of **c.** *Myh7*, **d.** *Nppa*, and **e.** *Nppb* also show adult treatment (P42) of *Alpk3*$^{KI/KI}$ with AAV9-TNNT2-*ALPK3* reduces cardiac hypertrophy markers to wild-type levels. **f.** Principal component analysis of proteomic data. **g.** Volcano plot of *Alpk3*$^{WT/WT}$ vs. *Alpk3*$^{KI/KI}$ AAV9-Control showing >1600 differentially expressed proteins compared to **h.** 81 differentially expressed proteins between *Alpk3*$^{WT/WT}$ vs. *Alpk3*$^{KI/KI}$ AAV9-TNNT2-*ALPK3*. Volcano plots of differentially phosphorylated peptides between **i.** *Alpk3*$^{WT/WT}$ vs. *Alpk3*$^{KI/KI}$ AAV9-Control and **j.** *Alpk3*$^{WT/WT}$ vs. *Alpk3*$^{KI/KI}$ AAV9-TNNT2-*ALPK3* revealed a 98% shift towards wild-type. **k.** Phosphorylation status of the potential ALPK3 target, pT269/S272 Sqstm1, showed expression of hsALPK3 increased pT269/S272 back to wild-type levels.

6. The authors mention that ALPK3 coordinates a protein quality control hub to maintain contractile protein turnover. The authors should check downstream contractile protein targets regulated through this mechanism that show rescue through AAV-human ALPK3 therapy.

We thank the reviewer for this insightful suggestion and agree that defining downstream contractile protein targets regulated by ALPK3-dependent proteostasis would further strengthen our conclusions.

To address this, we performed Gene Ontology (GO) enrichment analysis using g:Profiler (PMID: 21646343) on our quantitative proteomics dataset. Comparison of *Alpk3*$^{WT/WT}$ vs. *Alpk3*$^{KI/KI}$ + AAV9-Control hearts revealed enrichment of pathways in *Alpk3*$^{KI/KI}$ hearts associated with protein quality control, including "deubiquitinase activity", "ubiquitin-like peptidase activity", "response to heat", and "proteolysis" (**Response Figure 5a;** *in manuscript* **Figure 3g**). In contrast, pathways enriched in *Alpk3*$^{WT/WT}$ were largely related to mitochondria and translational machinery (**Response Figure 5b;** *in manuscript* **Figure 3h**). Together, these data indicate broad dysregulation of proteostasis in ALPK3-deficient hearts, spanning protein synthesis, folding, and degradation.

To determine whether these pathways are restored following gene therapy, we intersected differentially expressed proteins between *Alpk3*$^{WT/WT}$ vs. *Alpk3*$^{KI/KI}$ + AAV9-Control and *Alpk3*$^{KI/KI}$ + AAV9-TNNT2-*ALPK3* vs. *Alpk3*$^{KI/KI}$ + AAV9-Control. We identified 398 commonly upregulated and 509 commonly down regulated proteins (**Response Figure 5 c and d;** *in manuscript* **Figure 3j and k**). GO enrichment of commonly up regulated proteins were enriched in pathways related to the mitochondrion and translation, closely mirroring those in **Response Figure 5b**. Conversely, commonly down regulated proteins were enriched for "protein ubiquitination" and "response to heat", indicating a normalisation of proteostatic pathways. Taken together, these analyses demonstrate that AAV9-TNNT2-*ALPK3* gene therapy restores global proteostasis networks in the *Alpk3*$^{KI/KI}$ myocardium.

Focusing specifically on the contractile apparatus, we observed enrichment of the GO term "A band" in *ALPK3*$^{KI/KI}$ hearts, which was normalised upon AAV9-TNNT2-*ALPK3* treatment (**Response Figures 5a, d**). This prompted deeper analysis of sarcomeric protein abundance. Consistent with prior reports (Feng et. al, 2025, *Circulation*) we observed accumulation of thick filament proteins in *ALPK3*$^{KI/KI}$ hearts (**Response Figure 5e, Figure3l**). Additionally, we identified a significant reduction in thin filament protein levels, indicating a catastrophic imbalance in contractile protein stoichiometry in *ALPK3*$^{KI/KI}$ hearts. Importantly, AAV9-TNNT2-*ALPK3* treatment resulted in a near complete restoration of this balance (**Response Figure 5e;** *in manuscript* **Figure3l**), supporting our hypothesis that ALPK3 regulates sarcomere proteostasis.

To further explore regulatory mechanisms related to the role of ALPK3 in sarcomere proteostasis, we examined proteins that were selectively upregulated upon AAV9-TNNT2-*ALPK3* treatment. Notably, the most significantly increased proteins were Trim54 (Murf3), Trim55 (Murf2), and Trim63 (Murf1) (**Response Figures 5f-l;** *in manuscript* **Extended Data Figure 5i-k**). These are muscle enriched E3 ubiquitin ligases with established roles in the turnover of sarcomeric proteins (e.g. PMIDs: 15967462, 15199100, 34179788, 7983583). Consistent with this finding, we previously demonstrated an interaction between ALPK3 and TRIM55 in human pluripotent stem cell-derived cardiomyocytes, suggesting that ALPK3

contributes to sarcomeric proteostasis through recruitment or organisation of ubiquitin-mediated degradation machinery at the sarcomere.

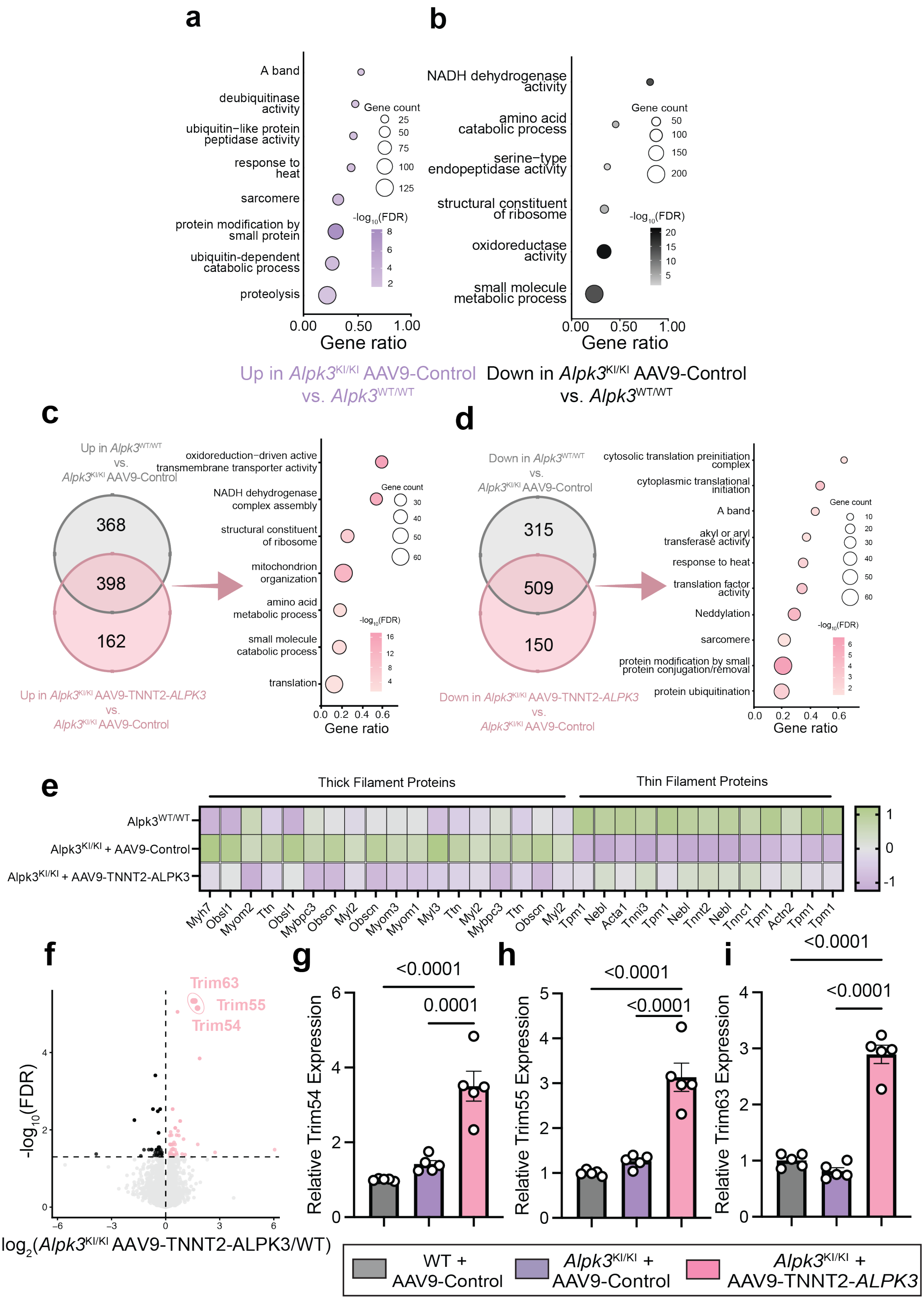

***Response Figure 5. ALPK3 gene therapy regulates protein quality control networks*. a.** Gene ontology (GO) terms enriched in *Alpk3*$^{KI/KI}$ AAV9-Control versus wild-type and **b.** GO terms repressed in *Alpk3*$^{KI/KI}$ AAV9-Control versus wild-type demonstrate dysregulation of proteostasis networks. **c. and d.** Intersection differentially expressed proteins shared between *Alpk3*$^{KI/KI}$ AAV9-TNNT2-*ALPK3* and WT versus *Alpk3*$^{KI/KI}$ AAV9-Control shows restoration of proteostasis terms. **e.** Analysis of sarcomeric protein expression between WT and *Alpk3*$^{KI/KI}$ AAV9-Control revealed abnormal accumulation of thick filament proteins and loss of thin filament proteins, which was normalised by AAV9-TNNT2-*ALPK3* treatment. **f-i.** AAV9-TNNT2-*ALPK3* treatment specifically increases expression of sarcomeric E3 ligases Trim 54, 55, and 63.

7.The authors should try to determine the amount of cells that were targeted after gene therapy by staining for human ALPK3.

Confirmation of the number of murine cardiomyocytes transduced by AAV9-TNNT2-*ALPK3* would be informative to understand the proportion of targeted cells required to achieve the complete disease reversal observed in this study. Unfortunately, we have been unable to validate an antibody, commercial or custom-synthesised, that is specific to ALPK3, and our therapeutic vector contains no fusion epitope tags (for a more detailed response see response to reviewer 1 points 2 and 3).

To overcome this, we attempted to perform fluorescence *in situ* hybridisation on mouse heart sections using a probe set from Molecular Instruments. Unfortunately, the probe set was unable to distinguish *Alpk3*$^{KI/KI}$ AAV9-TNNT2-*ALPK3* hearts from *ALPK3*$^{WT/WT}$ stained samples (**Response Figure 6**). Due to the proprietary nature of the probe set, we were unable to determine if this was due to the probe cross-reacting with mouse *Alpk3* or if there is another, non-specific binding event within mouse tissue.

While we are unable to directly address the reviewer's concern in this instance, we do not believe that this impacts the major findings of the manuscript. Firstly, in mouse studies, we have used the well-defined AAV9 capsid with known tropism for the heart. Previous studies using AAV9 delivered by intravenous injection at similar doses have reported cardiac transduction efficiencies well above 80% at equivalent doses of >1x10^{12} vector genomes when delivered intravenously (PMIDs: 16713360, 25082846, 20703310, 33580817, 26173818). Secondly, as described above, we have demonstrated robust overexpression of both human ALPK3 transcript and protein at levels that meet or exceed endogenous mouse *Alpk3* levels (**Response Figure 1**). Finally, and most critically, using the AAV9-mediated delivery of *ALPK3*, we have been able to completely restore both functional and molecular features of cardiomyopathy to a level that, to the best of our knowledge, has not previously been reported via gene therapy. This has included restoration of systolic and diastolic function, reversal of established cardiac and cellular hypertrophy, and >95% restoration of proteomic and phosphoproteomic signatures of disease. Given the likely scaffolding role of ALPK3 within the cardiac sarcomere, this level of restoration would not be expected if just a small fraction of cells were transduced. In summary, given the extensive phenotypic rescue by AAV9-TNNT2-*ALPK3* there is no reason to expect cardiac transduction levels to be appreciably different from published work using the AAV9 capsid.

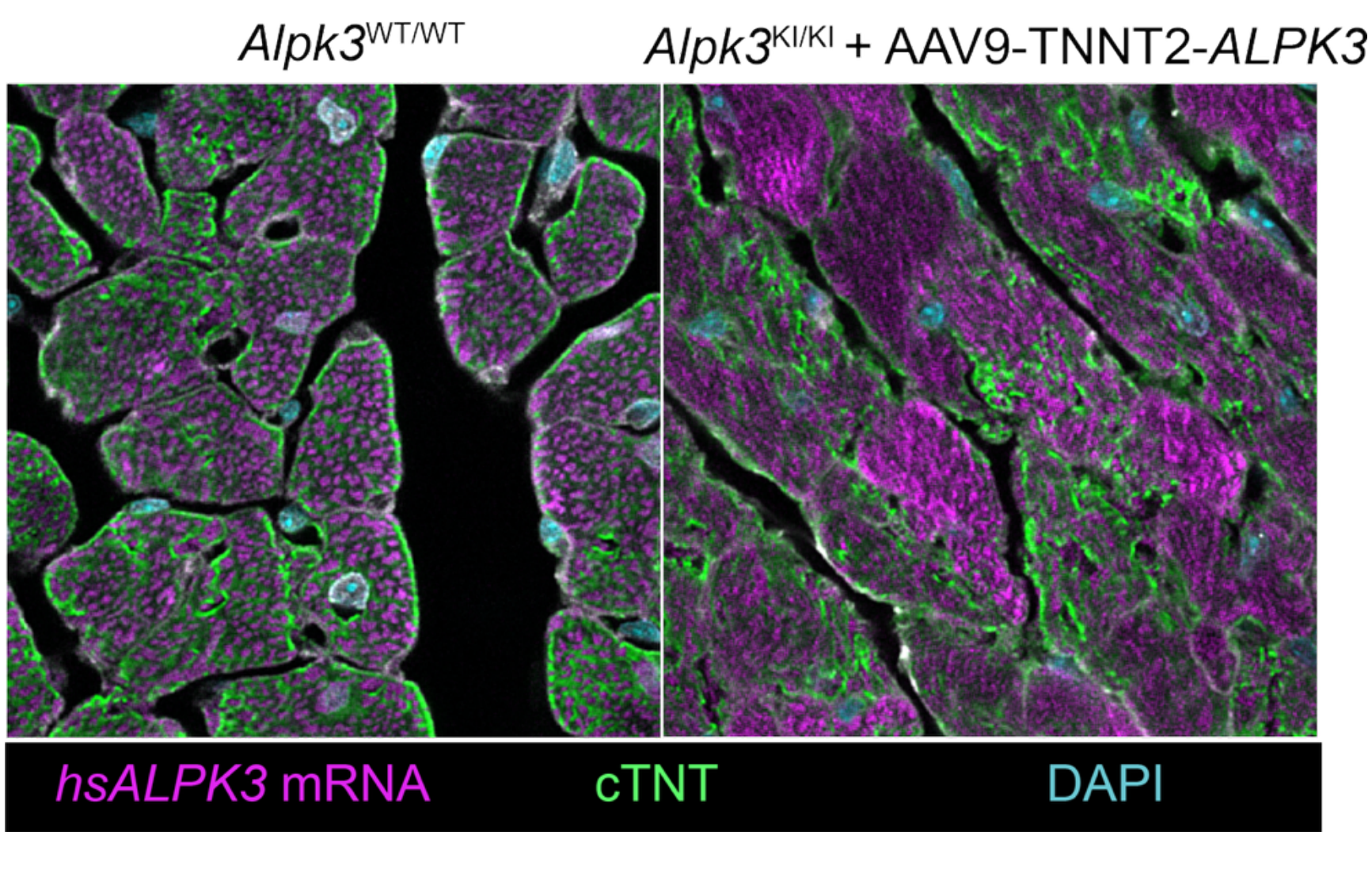

Response Figure 6.* Transduction efficiency of AAV9-TNNT2-*ALPK3. Myocardial sections were stained using a fluorescent *in situ* hybridisation probe against human *ALPK3* (magenta) and co-stained for cTNT protein (green) and DAPI (cyan). Unfortunately, no specific staining could be observed over wild-type hearts limiting this measurement.

8. Did the authors check the skeletal muscle and liver for expression of human ALPK3?

To assess the question of tissue specificity of transgene expression, we performed qPCR to assess both DNA and RNA levels in the heart, liver, and hindlimb muscle (tibialis anterior), using standard curves generated from *ALPK3* DNA of known copy number.

In cardiac tissue, we observed 4.6 ± 0.8 copies of *ALPK3* transgene DNA per diploid cell (**Response Figure 7a;** *in manuscript* **Extended Data Figure 2f**), corresponding to 3.6 ± 0.3x10^7 transgene copies per μg of RNA, indicating robust transgene expression (**Response Figure 7b;** *in manuscript* **Figure 3a)**.

As expected for systemically delivered AAV9, liver tissue exhibited substantially higher vector DNA levels, with 83 ± 16 copies per diploid cell (**Response Figure 7c, Extended Data Figure 6a**). However, despite the 20-fold increase in DNA copy number compared to heart tissue, hs*ALPK3* transcripts were only detected in one of three liver samples and at a markedly lower level (2.6 x10^6 transgene copies per μg of RNA; **Response Figure 7d;** *in manuscript* **Extended Data Figure 6b**).

In skeletal muscle, transgene DNA copy number was comparable to cardiac tissue (7 ± 2 DNA copies per diploid cell), but similar to liver, transcript expression was minimal, being detected in only one sample and at a substantially lower level (8.6 x10^5 copies per μg of RNA; **Response Figure 7e,f;** *in manuscript* **Extended Data Figure 6a,b**).

Together these data demonstrate a clear uncoupling of transgene vector biodistribution and transcript expression in non-cardiac tissues, consistent with effective transcriptional restriction. This is likely mediated by the cardiac specific *TNNT2* promoter and inclusion of miR-122a-5p target sites to suppress expression in the liver. Collectively, these data support cardiac selective expression of the ALPK3 transgene with minimal off-target impacts in the liver and skeletal muscle of this preclinical model.

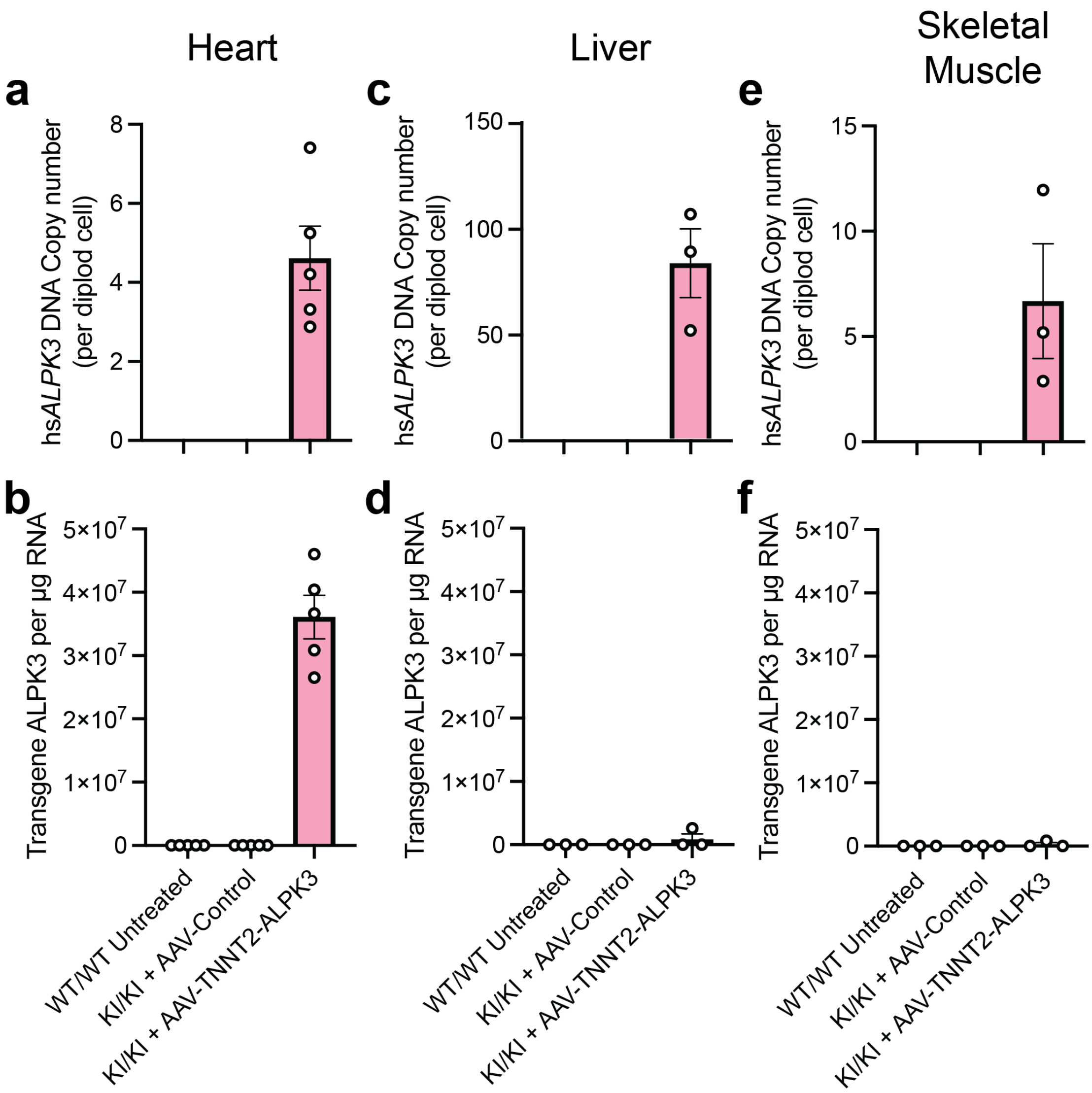

Response Figure 7. Restriction of ALPK3 transgene to cardiac tissues. **a.** qPCR and **b.** RT-qPCR against *ALPK3* demonstrated robust expression of *ALPK3* transgene mRNA in mouse hearts treated with AAV9-TNNT2-*ALPK3*, with ~3.6×10^7 copies of *ALPK3* mRNA per microgram of RNA. In liver, despite high vector DNA expression (**c**), *ALPK3* RNA was only detected in one out of three samples and at an order of magnitude lower than in the heart (**d**). Similar findings were observed in hindlimb skeletal muscle with vector DNA comparable to cardiac tissue (**e**), but *ALPK3* RNA only observed in one out of three samples, and 40-times lower than observed in cardiac tissue.

9. Related to Figure 3B, is there a staining for the WT control? This is vital to show that the ALPK3 level after the gene therapy is back to WT levels.

Unfortunately, we are unable to detect endogenous ALPK3 protein due to the inability to validate a commercially available or generate a custom-made antibody (see Response to Reviewer 1, Points 2 and 3). However, to partially address this concern, we have transduced monolayers of WT iPSC-CMs with AAV6-ALPK3-FLAG and stained for ALPK3 using an anti-FLAG antibody as a proxy of ALPK3 localisation. Here, we show that even in the presence of WT ALPK3, exogenous ALPK3 will localise to the M-band of the cardiac sarcomere (**Response Figure 8;** *in manuscript* **Extended Data Figure 8e**). While this does not directly assess the return of ALPK3 levels back to that of a WT level, it does demonstrate that the transgene localises to the correct cellular compartment. As outlined in detail in **response point 1 above**, RNA sequencing and proteomics of mouse cardiac tissue revealed that the delivery of AAV9-TNNT2-ALPK3 resulted in an expression level of hs*ALPK3* of ~250CPM versus ms*Alpk3* ~30CPM in wild-type mice (**Response Figure 1d,e**), and a hsALPK3 protein expression equivalent to wild-type mouse Alpk3 levels (**Response Figure 1g**). These data, together with the complete functional restoration observed in mouse and iPSC models, collectively demonstrating the ability to safely and efficaciously express ALPK3 at levels equivalent or higher than WT levels

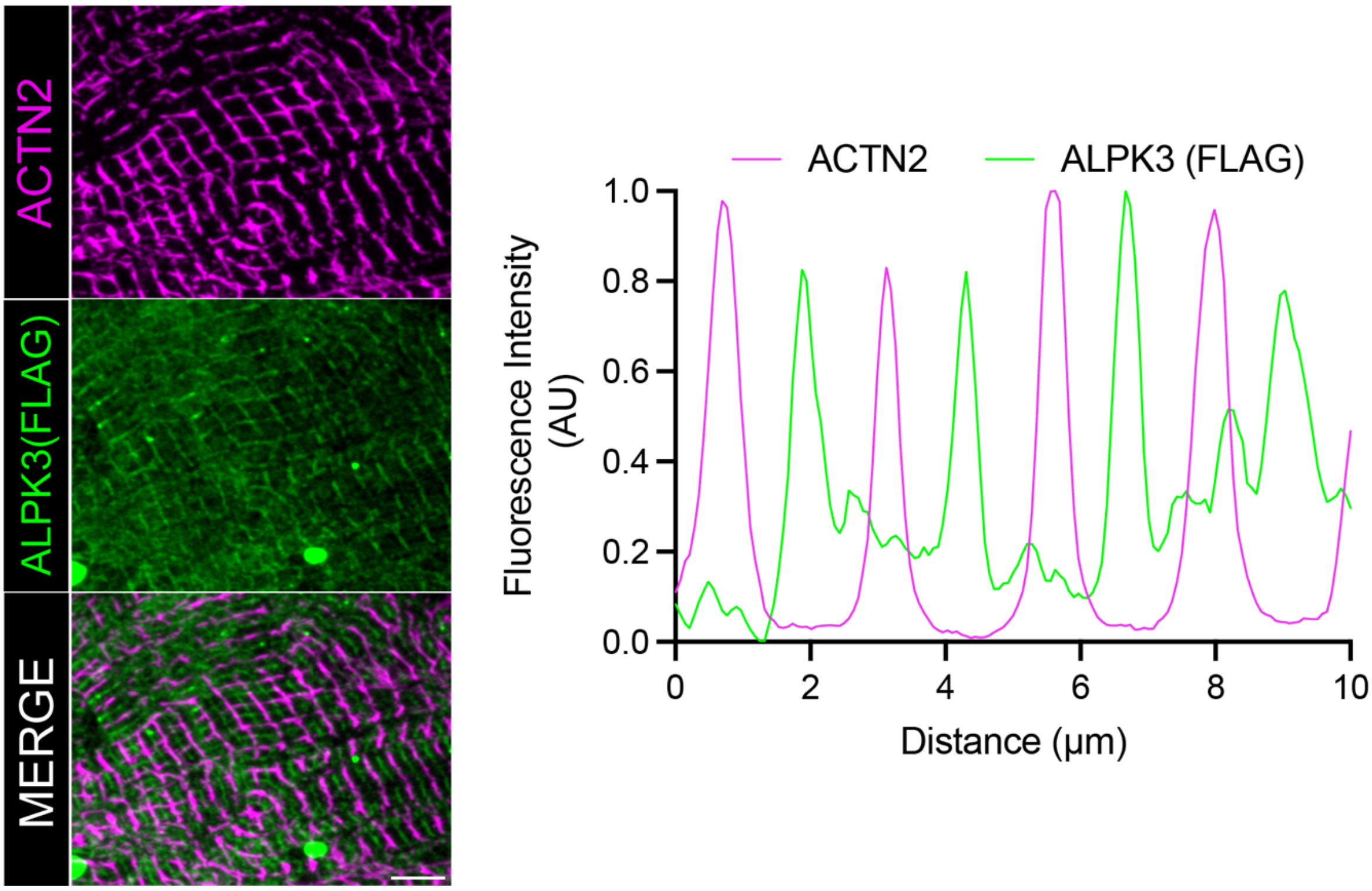

Response Figure 8. AAV6-ALPK3 correctly localises to M-Band in WT-treated hPSC-CMs. WT hPSC-CMs were treated with an AAV6-ALPK3-FLAG construct for 96 hours. Immunofluorescent staining of ALPK3 via FLAG-tag demonstrated clear localisation to the M-Band evident from its interdigitation with ACTN2, demonstrating the capacity to correctly localise transgenic ALPK3 in a wild-type setting.

Minor points:

1. Is there a reason why the control group in the adult mice study is 'untreated' in WT and not AAV-control (used in neonatal study)?

When we performed the neonatal study (treat P0, analyse P84, **Figure 1**), we included additional groups in which WT mice were treated with AAV9-TNNT2-*ALPK3*. This data was not included in the initial submission for brevity. However, these studies showed that over-expression of ALPK3 in a WT mouse had no impact on cardiac function (**Response Figure 9;** *in manuscript* **Extended Data Figure 4a-h**). Given this, we chose to focus on the impact of AAV9-TNNT2-*ALPK3* within only *Alpk3* KI mice. Thus, the WT AAV9-control group was not required as a control group. We therefore used only untreated WT mice as a control group to quantify the level of rescue in *Alpk3* KI mice following treatment with AAV9-TNNT2-*ALPK3.*

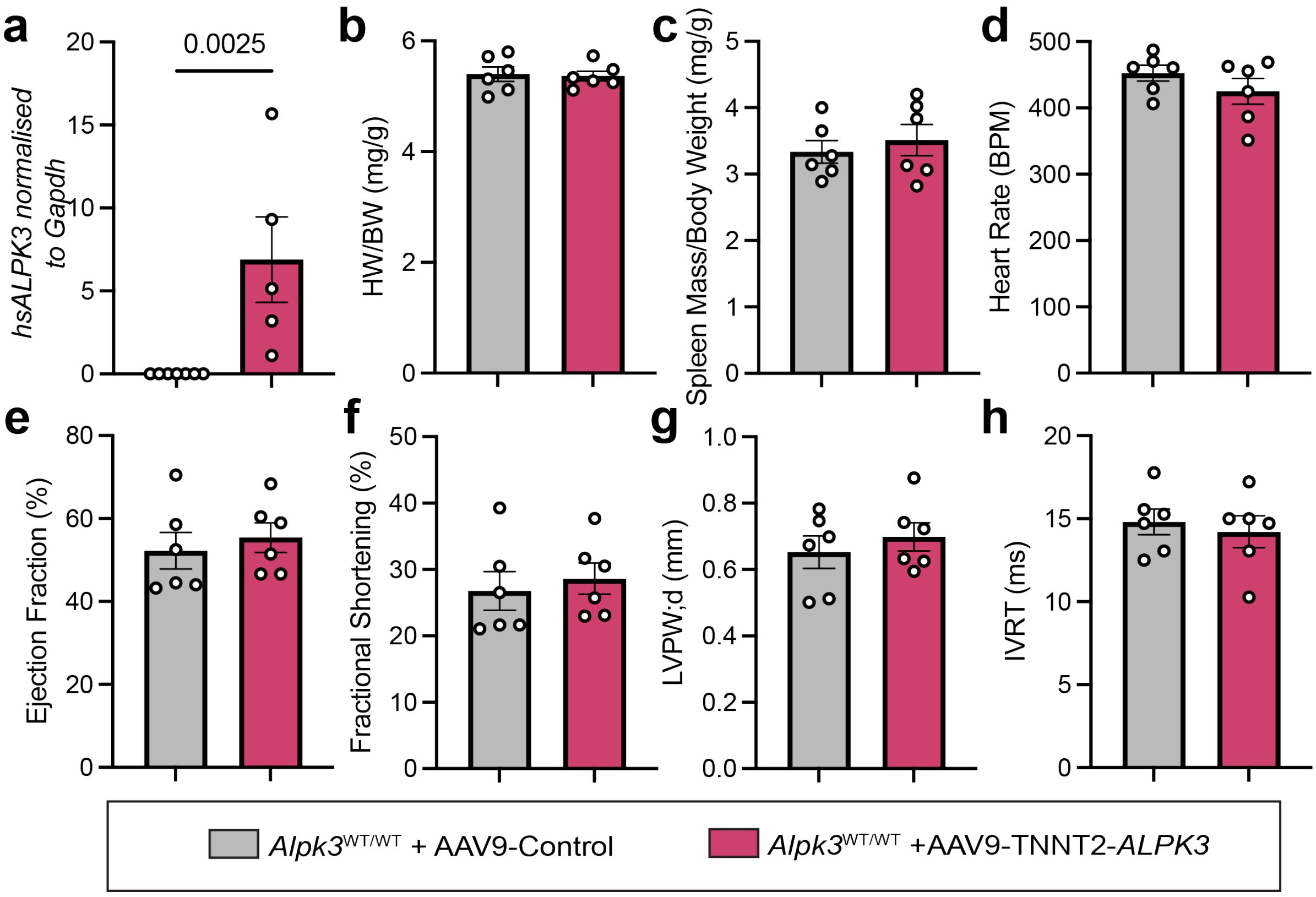

Response Figure 9. Overexpression of ALPK3 in a wild-type mouse minimally impacts cardiac function. **a.** hs*ALPK3* gene expression shows robust overexpression compared to control. No differences were observed in **b.** heart weight to body weight, **c.** spleen weight to body weight; or echocardiography parameters heart rate (**d**), ejection fraction (**e**), fractional shortening (**f**), left ventricular posterior wall thickness (**g**), or isovolumic relaxation time (**h**).

2. Data referral to 'extended data 1d-g' but extended data only goes to 'f'.
and
3. Different fonts in different panels of the same figure.

We apologise for these errors. Within this revised version, we have maintained fonts across all panels of the manuscript.

4. Is there a rationale for why the neonates were treated with a viral load of 2x1014 vg/kg but in adult mice 1x1014 vg/kg?

The neonatal study was performed prior to the adult study and we chose to use a dose at the high end of the clinical safety spectrum ($2x10^{14}$ vg/kg). Given the strong results observed in this study, we decided to attempt a dose reduction in the adult mice. This clearly demonstrated the ability to reduce the viral load when delivered intravenously. Future studies will further address this via a dose de-escalation study to attempt to reduce viral load further.

5. The labelling in general is very inconsistent in the figures.

We have reviewed all figures and now have consistent presentation.

6. Wrong figure call-out throughout the text, please check.

In this revised version, we have carefully cross checked each figure callout within the text to ensure the data are presented clearly.

7. Figure 2, missing the statistical comparison labels.

Given the large effect sizes, we chose to omit statistical comparison labels in Figure 2 to improve viewability of the data and instead included a supplementary table outlining the Two-way ANOVA tests. However upon completing a longer-term study for this adult intervention, we now include these labels.

8. In figure 3, authors do not specify which ALPK3 mutated cell line was used.

We use the $ALPK3^{mut}$ hPSC line previously published in Phelan, et. al (*European Heart Journal, 2016*) McNamara, et. al (Nature Cardiovascular Research, 2023). This line is a female human embryonic stem cell line (HES3, WiCell ES03) that has been edited to carry the $NKX2\text{-}5^{GFP/+}$ allele and a homozygous deletion within exon 1. This has now been included within the methodology.

9. For figure 3f, the authors do not refer where the single-cell data for the pseudobulk analysis came from.

We apologise for the confusion. This data has been generated within the laboratory using the same iPSC line carrying a *TTN* truncating variant. This has been updated in the methodology, and the relevant single cell dataset has been deposited to the Gene Expression Omnibus to be publicly available (GSE328808; reviewer token gfudsoiknrulxqd). Additionally, all proteomics datasets have been deposited to the PRotein IDEntifications Database (PRIDE) (accession number PXD077620; reviewer only token 3l4h7uhFAZil).

10. In Extended Data Figure 1b, is the 'AAV6-Empty' label in the graph a typo as all the other groups are labelled with 'AAV9'?

Thank you for pointing out this typographical error. We have corrected to "AAV9-Control" for consistency with the rest of the manuscript.

Reviewer #2 (Remarks to the Author):
MAJOR COMMENTS
1. From an AAV/packaging capacity (4.7kb) point of view, I am wondering how the authors' approach compares to the recently published miniALPK3 approach by Feng et al. ALPK3 encodes a 1705AA (~5.1 kb) CDS (without any regulatory elements) which is far above the classical AAV packaging limit (~4.7 kb). This is exactly why others went the "miniALPK3" route. The manuscript states that "full-length human ALPK3" was delivered by AAV9 under a TNNT2 promoter but does not provide a schematic of the expression cassette (promoter, WPRE, polyA, any introns, tags). The authors should address that they are using an "oversized AAV" strategy and whether they see concatemer formation or truncated genomes on Southern blot/qPCR. I don't mean this in a "novelty" sense re miniALPK3 but rather in a technical sense. The authors should also show: - a vector map and total ITR-to-ITR size.

The reviewer raises several important considerations. Full-length *ALPK3* coding sequence (1705 aa; ~5.1 kb) exceeds the classical AAV packaging limit of ~4.7 kb. Through rational vector design, including deletion of non-essential stuffer sequences and replacement of the 208 bp BGH polyadenylation sequence with a 50 bp synthetic polyadenylation sequence, we were able to substantially reduce the size of the expression cassette. This optimisation reduced the packaging insert from our original design of ~6.2 kb to ~5.8 kb. As suggested by the reviewer, we now include a schematic of the expression cassette and total construct architecture (**Response Figure 10a;** *in manuscript* **Extended Data Figure 2a**).

In vivo, this optimisation resulted in approximately a five-fold increase in expression relative to the original 6.2 kb design, despite the use of the weaker, cardiac-specific TNNT2 promoter (**Response Figure 10b,** *in manuscript* **Extended Data Figure 2b**). Notably, this increase in expression correlated with greater functional protection compared with the original CMV-driven construct (**Response Figure 10c-e;** *in manuscript* **Extended Data Figure 2c-e**), supporting the positive functional impact of ALPK3 expression. We have added new text addressing this within paragraph 3 as follows: "Pilot studies demonstrated five-fold stronger hs*ALPK3* expression using the cardiotropic *TNNT2* promoter (Extended Data Fig. 2a) compared to the ubiquitous CMV promoter (Extended Data Fig. 2b-e). This likely results from the optimisation of this therapeutic vector over the CMV-driven construct promoter, including replacement of the bovine growth hormone polyadenylation sequence with a 53bp synthetic polyadenylation signal sequence and removal of non-regulatory spacer sequence, ultimately resulting in a 300-nucleotide reduction in vector genome size."

While these data support the feasibility of the approach, they do not directly address the possibility of truncated vector genomes. To examine this, we performed qPCR on cardiac tissue using primer pairs located at the 5′ and 3′ ends of the transgene. Although both DNA and RNA copy number showed a modest trend toward reduction at the 3′ end of the transgene, this difference was not statistically significant (**Response Figure 10f ,g;** *in manuscript* **Extended Data Figure 2g,h**).

- data confirming that the predominant expressed product in vivo is truly full-length ALPK3 (e.g. Western blot from mouse hearts, not only FLAG-IF in monolayers)

To further assess whether full-length protein is produced in vivo, we performed quantitative proteomics on mouse hearts treated with AAV9-TNNT2-ALPK3 and examined the distribution

of human-specific ALPK3 peptides across the length of the protein (**Response Figure 10h; Extended Data Figure 5c**). Peptides were detected across the entire protein with comparable log_2expression levels, indicating that full-length human ALPK3 protein is produced in vivo. Indeed, the abundance of human ALPK3 peptides was equivalent to or greater than endogenous mouse Alpk3 (**Response Figure 1g;** *in manuscript* **Extended Data Figure 5d**). We do note that due to the lower abundance of tryptic peptides between amino acids 840 and 1142 (shown in purple), we could not detect protein that region. However we believe that results as a technical artefact of mass spectrometry rather than truncation of the ALPK3 product, supported by the qPCR analysis. To demonstrate the reduced density of tryptic peptides in this region, we have plotted the detected peptides against the predicted peptides (assessed using Expasy Peptide Cutter). Together, these data strongly support that despite the likelihood of some truncated vector genomes, functional full-length ALPK3 protein is expressed in the heart.

- at least a short discussion on how they see this vector being advanced clinically, given packaging limits and the competing miniALPK3 concept. It would be best to compare both systems (mini vs. full-length) side-by-side in vivo using the same AAV9 and promoter architecture.

It is important to note that oversized vectors have been clinically approved, for example Roctavian (valoctocogene roxaparvovec, PMID 29292164) was approved for the treatment of haemophilia. This suggests that with robust quality control measures in vector production, oversized AAV vectors with strong therapeutic efficacy can be advanced clinically.

The reviewer also raises an important point regarding the recently published miniALPK3 gene therapy strategy described (PMID: 41248220). This is an elegant study in which a miniaturised ALPK3 construct was designed based on sequence conservation across species. In their neonatal protection model, delivery of 2×10^{14} vg/kg miniALPK3 under control of the TNNT2 promoter using a myoAAV capsid resulted in partial functional protection (Fractional Shortening (%): WT ~45%; $Alpk3^{-/-}$ ~22%; $Alpk3^{-/-}$ + myoAAV-miniALPK3 ~37%).

In comparison, our neonatal protection study using the same dose (albeit delivered via IP injection) of AAV9-TNNT2-ALPK3 resulted in near-complete functional protection (Fractional Shortening (%): WT ~27%; *Alpk3*$^{KI/KI}$ ~14%; *Alpk3*$^{KI/KI}$ + AAV9-TNNT2-ALPK3 ~27%), and we additionally observed complete restoration of function in an adult disease-reversal model (Fractional Shortening (%): WT ~26%; *Alpk3*$^{KI/KI}$ ~14%; *Alpk3*$^{KI/KI}$ + AAV9-TNNT2-ALPK3 ~27%). While these studies differ in model, capsid, and delivery route, these findings raise the possibility that regions removed in the miniALPK3 construct may contain functionally important elements required for full ALPK3 activity.

Another consideration for miniaturised therapeutic proteins is the potential generation of neo-epitopes at the junctions created by domain fusion. In other gene-therapy contexts, such as micro-dystrophin constructs developed for Duchenne muscular dystrophy, truncated or fusion proteins have been reported to provoke immune responses in some experimental settings, including inflammatory myopathy and transient transgene expression (PMIDs: 34569971, 37314712, 20925545 among others). Although this does not preclude the success of miniaturised constructs, it highlights the importance of carefully evaluating immunogenicity

and long-term safety. Whether similar considerations will apply to miniALPK3 constructs in human therapy remains to be determined.

Therefore, while reducing vector genome size is an important consideration for the development of ALPK3 gene therapies, both full-length and miniaturised approaches present potential advantages and limitations. Importantly, the robust disease reversal observed following adult treatment with AAV9-TNNT2-ALPK3 in our study supports the continued development of the full-length strategy as a tractable therapeutic approach. We have included text addressing these points within the discussion starting as follows:

"The use of an oversized AAV like AAV9-TNNT2-*ALPK3* is an important consideration for a clinical development program. Importantly, our oversized construct resulted in expression of full-length ALPK3 and resulted in high efficacy, supporting the clinical translatability of this approach. The feasibility of clinical development of oversized AAV constructs is further supported by the clinical approval and therapeutic success of Roctavian for the treatment of haemophilia.[23] An alternate strategy for larger genes is to miniaturise the protein while retaining key functional domains which has been championed in gene therapies for Duchenne Muscular Dystrophy.[24] Recent work has described a miniALPK3 construct delivered by the myoAAV capsid, which partially restored cardiac function in *Alpk3* knockout mice treated neonatally via retro-orbital injection.[25] However, the miniALPK3 offered only partial functional rescue in a neonatal protection model (Fractional Shortening (%): WT ~45%; Alpk3$^{-/-}$ ~22%; Alpk3$^{-/-}$ + myoAAV-miniALPK3 ~37%).[25] In comparison, neonatal injection at a similar dose using full-length AAV9-TNNT2-*ALPK3* in this study, albeit delivered via the less efficient IP injection route, resulted in near-complete prevention of cardiac dysfunction (Fractional Shortening (%): WT ~27%; *Alpk3*$^{KI/KI}$ ~14%; *Alpk3*$^{KI/KI}$ + AAV9-TNNT2-ALPK3 ~27%). Furthermore, we observed complete restoration of function in an adult disease-reversal model (Fractional Shortening (%): WT ~26%; *Alpk3*$^{KI/KI}$ ~14%; *Alpk3*$^{KI/KI}$ + AAV9-TNNT2-ALPK3 ~27%). These findings raise the possibility that the miniALPK3 construct lacks functionally important domains of ALPK3. Additionally, miniaturised gene therapies have the potential for the generation of immune-modulatory "neo-epitopes" at the junctions created by domain fusion of the miniaturised protein. For example, in micro-dystrophin constructs developed for Duchenne muscular dystrophy, the miniaturised proteins have been reported to provoke immune responses resulting in inflammatory myopathy and transient transgene expression.[26] Although this does not preclude the success of miniaturised constructs, it highlights the importance of carefully evaluating immunogenicity and long-term safety. Whether similar considerations will apply to miniALPK3 constructs in human therapy remains to be determined. Therefore, while reducing vector genome size is an important consideration for the development of *ALPK3* gene therapies, our data support the use of full-length *ALPK3*."

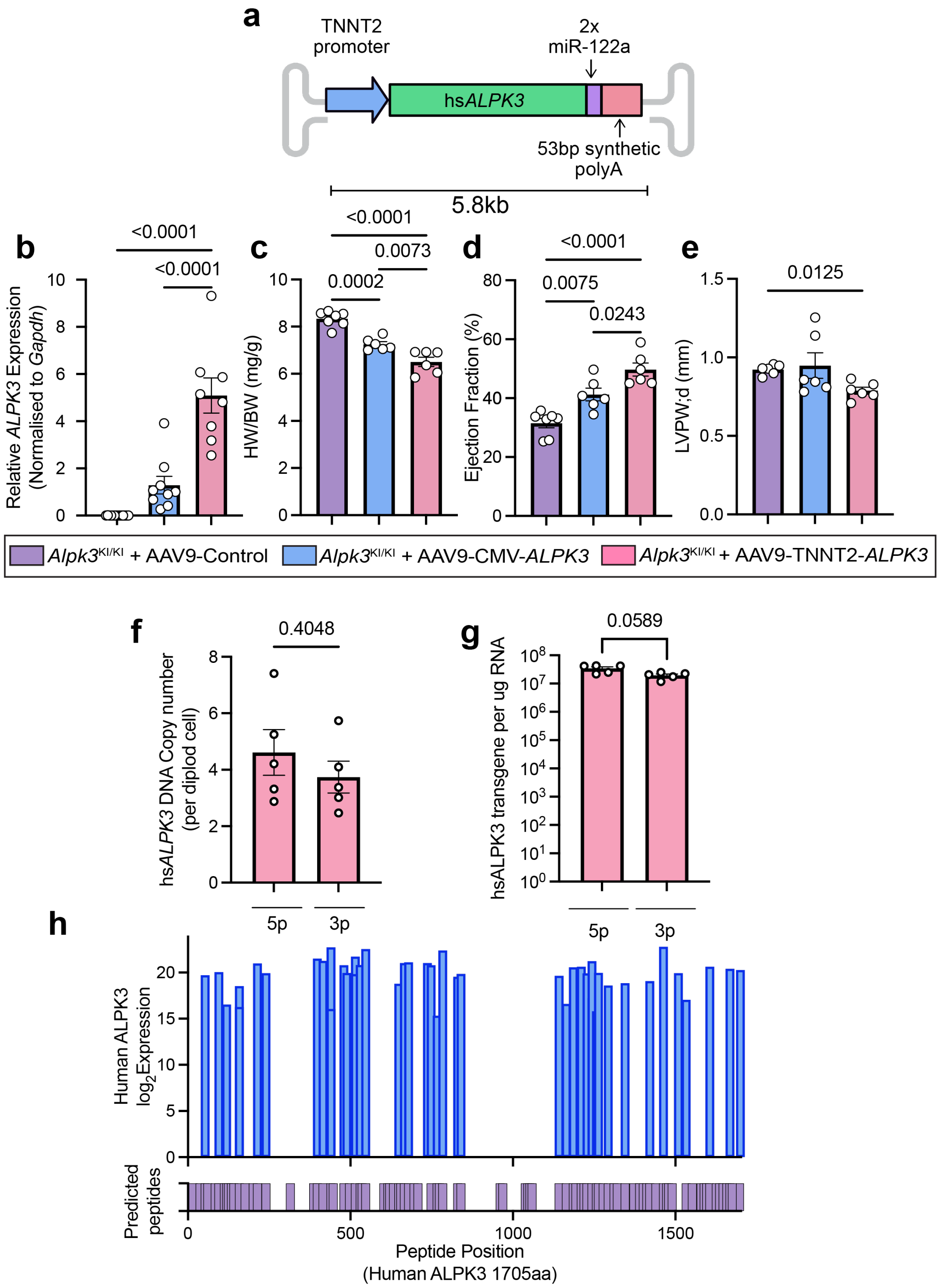

Response Figure 10. Delivery of oversized AAV ALPK3 construct demonstrates expression of full-length products at therapeutic levels. **a.** Schematic of the AAV-TNNT2-*ALPK3* expression construct. Comparison of the optimised AAV9-TNNT2-*ALPK3* to AAV9-CMV-*ALPK3* revealed significantly greater cardiac transgene expression (**b**), reduced cardiac mass (**c**), increased ejection fraction (**d**), and reduced ventricular wall thickness (**e**). To assess potential truncations due to the oversized genome, qPCR and RT-qPCR was performed using primer pairs a the 5p and 3p end of the transgene No difference was observed between the expression of vector *ALPK3* at the 5p and 3p (**f**), while a trend was observed for slightly reduced expression of 3p *ALPK3* compared to 5p *ALPK3* (**g**). However, this did not impact protein expression levels, with analysis of human specific peptides in mouse hearts treated with AAV9-TNNT2-*ALPK3* showing comparable expression across the length of the protein (**h**). The predicted tryptic peptides are shown below in purple.

2. The paper infers proteostasis restoration at the sarcomeric M-band to explain efficacy across ALPK3-deficient and TTN-truncation models but does not measure established proteostasis endpoints (LC3 flux, p62, ubiquitin conjugates, proteasome activity) in Figures 1– Absent these pathway-level readouts, the causal link between ALPK3 transduction and proteostasis normalization remains unproven, weakening rationale for indication expansion.

We thank the reviewer for raising this important point regarding the need for established proteostasis readouts and the causal relationship between ALPK3 expression and proteostasis restoration.

We performed quantitative proteomic and pathway-level analyses to evaluate proteostasis networks *in vivo*. In adult treated hearts, pathway enrichment analysis of differentially expressed proteins (FDR < 0.05) identified significant dysregulation of proteostasis-associated pathways in *Alpk3*$^{KI/KI}$ AAV9-Control hearts, including proteolysis, ubiquitin-like protein peptidase activity, heat shock response, and protein degradation pathways (**Response Figure 11a;** *in manuscript* **Figure 3g**), alongside reductions in pathways related to amino acid metabolism and endopeptidase activity (**Response Figure 11b;** *in manuscript* **Figure 4h**). To further validate these findings, we performed a chymotrypsin-like activity assay on ventricular tissue. This revealed reduced proteasome activity in *Alpk3*$^{KI/KI}$ AAV9-Control, which was completely normalised to wild-type levels with AAV9-TNNT2-*ALPK3* (**Response Figure 11c;** *in manuscript* **Extended Data Figure 5l**). Importantly, these pathways were normalised following AAV9-TNNT2-ALPK3 treatment, as demonstrated by analysis of shared differentially expressed proteins across comparisons (**Response Figure 11d;** *in manuscript* **Figure 3j,k**), indicating restoration of global proteostasis networks. At more granular level, we observed dysregulation of several well-established proteostasis markers. For example, Sqstm1/p62, Calpain-2, and αB-crystallin (Cryab) were elevated in *Alpk3*$^{KI/KI}$ AAV9-Control hearts and restored to wild-type levels following *ALPK3* gene therapy. Conversely, the ER chaperone BiP (Hspa5) was reduced in disease and similarly normalised with treatment (**Response Figure 11e;** *in manuscript* **Extended Data Figure 5e-h**). These data provide clear support for impaired proteostasis in ALPK3-deficient hearts and its restoration following gene therapy.

Thus, the revised manuscript provides substantial, multiparametric, evidence supporting the hypothesis that the restoration of proteostasis a major component of the phenotypic rescue elicited by the ALPK3 gene therapy. While we did not directly assess canonical flux-based assays (e.g. LC3 flux or proteasome activity) in this study, they remain a priority in our group and the subject of on-going experiments Defining the precise biochemical pathways (e.g. autophagic flux) underlying this effect, will require dedicated follow-up studies, that are on-going and lie outside the scope of this manuscript. However, the convergent normalisation of proteostasis-associated pathways, key regulatory proteins, and sarcomeric structure (**Response Figure 3**) strongly supports a model in which ALPK3 expression restores proteostatic balance in the heart.

With respect to the extension of this mechanism to TTN-truncation (*TTNtv*) cardiomyopathy, prior studies have demonstrated that *TTNtv* leads to sarcomeric proteinopathy, altered E3 ligase localisation, and dysregulated ubiquitination (PMIDs: 36525964, 40473933, 26315439). Together with our observation of a shared ALPK3–TTN interactome enriched for autophagy

and proteasome regulators (*in manuscript* **Figure 4f**), these findings provided a sound rationale for testing *ALPK3* gene therapy in *TTNtv* -related cardiomyopathy.

To further explore this relationship, we applied Geneformer, a foundational AI model for network biology, to perform *in silico* genome-wide combinatorial gene perturbation, deleting each gene in combination with *ALPK3* and comparing the predicted impact to deleting *ALPK3* alone. The input data corpus for this analysis was human foetal cardiomyocytes from the Fetal Cell Atlas (PMID: 33184181). Notably, combined deletion of *ALPK3* and *TTN* produced the most pronounced predicted disruption in cardiomyocyte state (**Response Figure 11f**; *in manuscript* **Figure 4e**), supporting the concept that these proteins function within a shared regulatory network.

To understand the molecular consequences of ALPK3 expression in $TTN^{tv/+}$ cardiac organoids, proteomics was performed to compare the signature of $TTN^{tv/+}$ AAV6-Control and $TTN^{tv/+}$ AAV6-*ALPK3*. Differentially expressed proteins were enriched for pathways related to proteasome function, proteolysis, cytoskeletal organisation, and ribonucleotide metabolism (**Response Figure 11g,h;** *in manuscript* **Figure 4j,k**), consistent with modulation of proteostasis networks. Furthermore, integration of mouse and human datasets identified 59 shared proteins, enriched for proteins involved in proteostasis, cellular stress responses, cytoskeletal organisation, and metabolic regulation (**Response Figure 11i;** *in manuscript* **Figure 4l**).

Collectively, our multi-layered analyses across pathway enrichment, protein-level changes, cross-model validation, and functional rescue provide strong evidence that *ALPK3* gene therapy restores proteostasis networks in diseased myocardium. These findings support, but do not exclusively prove, a proteostasis-mediated mechanism underlying the therapeutic benefit.

Response Figure 11 (next page). ALPK3 gene therapy restores proteostasis in ALPK3 mouse hearts and TTNtv cardiac organoids. GO terms enriched (**a**) and repressed (**b**) in *ALPK3*$^{KI/KI}$ AAV9-control versus *Alpk3*$^{WT/WT}$ demonstrate global dysregulation of proteostasis networks including heat shock proteins, ubiquitin-dependent catalysis, and translation. **c.** Chymotrypsin-like activity assays confirmed impaired proteasome function in *ALPK3*$^{KI/KI}$ AAV9-control, which was normalised with AAV9-TNNT2-*ALPK3* treatment. **d.** Intersection of differentially expressed proteins shared between *Alpk3*$^{KI/KI}$ AAV9-TNNT2-*ALPK3* and WT versus *Alpk3*$^{KI/KI}$ AAV9-Control shows restoration of global proteostasis upon *ALPK3* gene therapy. **e.** Proteomics analysis demonstrated dysregulated expression of proteostasis regulator Sqstm1 (p62), Capn2, Cryab, Hspa5 (Bip), which was abrogated by *ALPK3* gene therapy. **f.** Geneformer prediction of impact of dual gene deletion identified *TTN* as the most damaging combination in tandem with *ALPK3.* **g.** Volcano plot of proteomic data comparing *TTN*$^{tv/+}$ organoids treated with AAV6-Control or AAV6-ALPK3. **h.** GO term enrichment of differentially expressed proteins demonstrates regulation of proteostasis pathways by *ALPK3* gene therapy. **i.** Intersection of differentially expressed proteins in mouse LV tissue (*Alpk3*$^{KI/KI}$ AAV9-Control vs. AAV9-TNNT2-*ALPK3*) and human organoid tissue (*TTN*$^{tv/+}$ AAV6-Control vs. AAV6-*ALPK3*) identified a core regulatory network of ALPK3-regulated proteins.

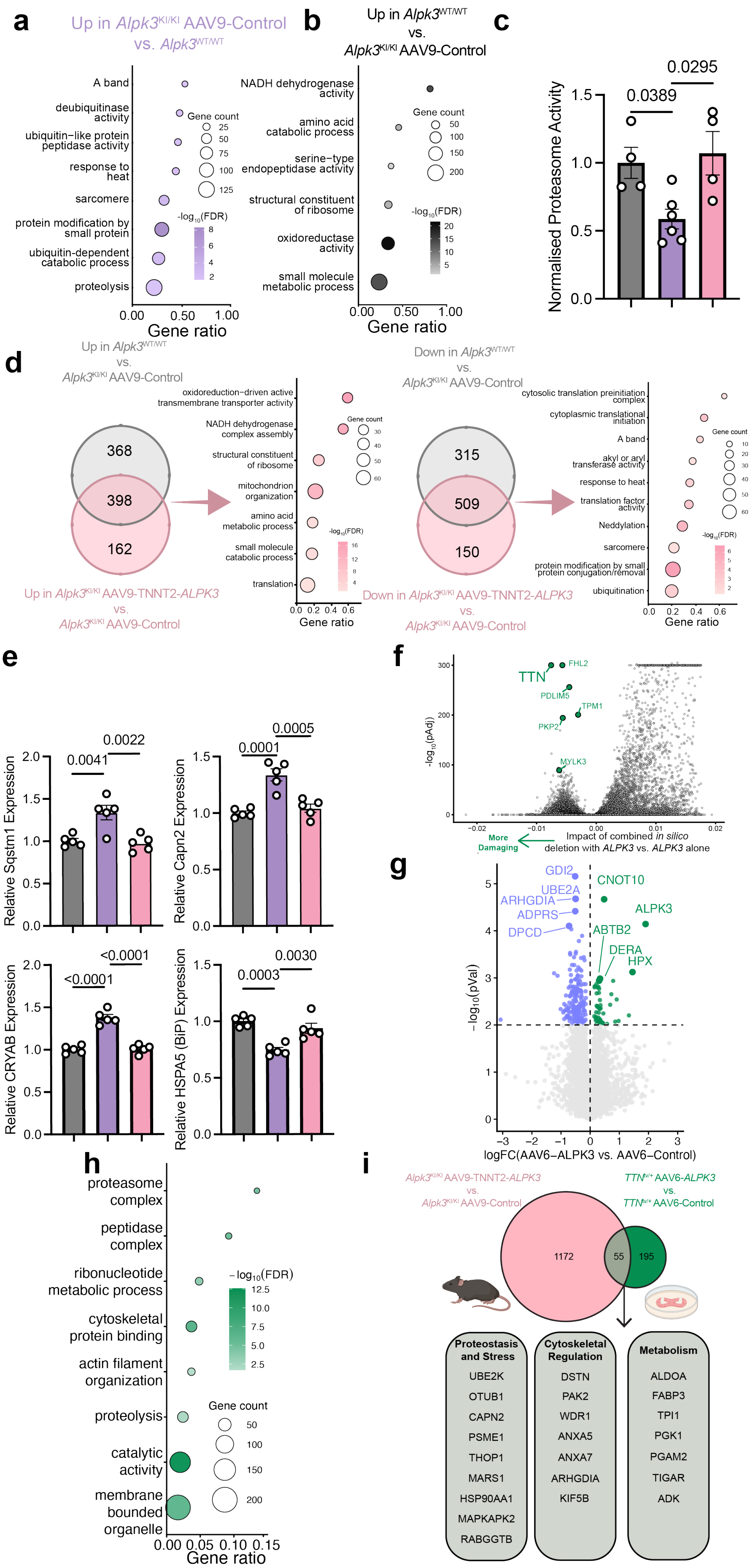

a
Up in Alpk3KI/KI AAV9-Control vs. Alpk3WT/WT
A band
deubiquitinase activity
ubiquitin−like protein peptidase activity
response to heat
sarcomere
protein modification by small protein
ubiquitin-dependent catabolic process
proteolysis
Gene count
25
50
75
100
125
-log10(FDR)
8
6
4
2
0.00
0.50
1.00
Gene ratio
b
Up in Alpk3WT/WT vs. Alpk3KI/KI AAV9-Control
NADH dehydrogenase activity
amino acid catabolic process
serine−type endopeptidase activity
structural constituent of ribosome
oxidoreductase activity
small molecule metabolic process
Gene count
50
100
150
200
-log10(FDR)
20
15
10
5
0.00
0.50
1.00
Gene ratio
c
Normalised Proteasome Activity
0.0
0.5
1.0
1.5
0.0389
0.0295
d
Up in Alpk3WT/WT vs. Alpk3KI/KI AAV9-Control
368
398
162
Up in Alpk3KI/KI AAV9-TNNT2-ALPK3 vs. Alpk3KI/KI AAV9-Control
oxidoreduction−driven active transmembrane transporter activity
NADH dehydrogenase complex assembly
structural constituent of ribosome
mitochondrion organization
amino acid metabolic process
small molecule catabolic process
translation
Gene count
30
40
50
60
-log10(FDR)
16
12
8
4
0.0
0.2
0.4
0.6
Gene ratio
Down in Alpk3WT/WT vs. Alpk3KI/KI AAV9-Control
315
509
150
Down in Alpk3KI/KI AAV9-TNNT2-ALPK3 vs. Alpk3KI/KI AAV9-Control
cytosolic translation preinitiation complex
cytoplasmic translational initiation
A band
akyl or aryl transferase activity
response to heat
translation factor activity
Neddylation
sarcomere
protein modification by small protein conjugation/removal
ubiquitination
Gene count
10
20
30
40
50
60
-log10(FDR)
6
5
4
3
2
0.0
0.2
0.4
0.6
Gene ratio
e
Relative Sqstm1 Expression
0.0
0.5
1.0
1.5
2.0
0.0041
0.0022
Relative Capn2 Expression
0.0
0.5
1.0
1.5
0.0001
0.0005
Relative CRYAB Expression
0.0
0.5
1.0
1.5
2.0
<0.0001
<0.0001
Relative HSPA5 (BiP) Expression
0.0
0.5
1.0
1.5
0.0003
0.0030
f
-log10(pAdj)
0
100
200
300
TTN
FHL2
PDLIM5
TPM1
PKP2
MYLK3
-0.02
-0.01
0.00
0.01
0.02
More Damaging
Impact of combined in silico deletion with ALPK3 vs. ALPK3 alone
g
GDI2
UBE2A
ARHGDIA
ADPRS
DPCD
CNOT10
ALPK3
ABTB2
DERA
HPX
−log10(pVal)
0
1
2
3
4
5
−3
−2
−1
0
1
2
3
logFC(AAV6−ALPK3 vs. AAV6−Control)
h
proteasome complex
peptidase complex
ribonucleotide metabolic process
cytoskeletal protein binding
actin filament organization
proteolysis
catalytic activity
membrane bounded organelle
−log10(FDR)
12.5
10.0
7.5
5.0
2.5
Gene count
50
100
150
200
0.00
0.05
0.10
0.15
Gene ratio
i
Alpk3KI/KI AAV9-TNNT2-ALPK3 vs. Alpk3KI/KI AAV9-Control
TTNsl/+ AAV6-ALPK3 vs. TTNsl/+ AAV6-Control
1172
55
195
Proteostasis and Stress
UBE2K
OTUB1
CAPN2
PSME1
THOP1
MARS1
HSP90AA1
MAPKAPK2
RABGGTB
Cytoskeletal Regulation
DSTN
PAK2
WDR1
ANXA5
ANXA7
ARHGDIA
KIF5B
Metabolism
ALDOA
FABP3
TPI1
PGK1
PGAM2
TIGAR
ADK

3. Vector dose, cardiac vector genomes, ALPK3 mRNA/protein expression levels, and safety need more rigorous characterization. The study uses relatively high systemic AAV doses, particularly in neonatal mice. Beyond gross measures such as body and spleen weight, additional data are needed on cardiac vector genome copy number, expression relative to endogenous ALPK3, biodistribution, and markers of cardiac and hepatic toxicity. Inclusion of WT animals treated with AAV-ALPK3 would be critical to define the consequences of supraphysiologic ALPK3 expression. Of note, without measuring cardiac ALPK3 mRNA/protein levels and vector genome copies in the heart, it is not possible to causally associate the improvements shown in Figures 2c–2g with cardiomyocyte transduction from the timeline in Figure 2a.

We acknowledge and agree with the reviewer doses used in this study are at the higher end of those typically considered for clinical translation. However, given the rapid and highly efficacious rescue observed, we expect that dose de-escalation will be feasible, and this forms part of our planned future studies.

While not included in the initial submission, we did assess the consequences of supraphysiological *ALPK3* expression in wild-type mice treated with AAV9-TNNT2-*ALPK3*. We have now included this data (**Response Figure 9;** *in manuscript* **Extended Data Figure 4a-h**), which resulted in no detectable impairment in cardiac function, suggesting that elevated *ALPK3* expression is well tolerated in the healthy heart.

We have now performed an analysis of vector genome and transgene copy number across tissues. In adult mice, we observed approximately 5, 83, and 7 copies of *ALPK3* vector DNA per diploid cell in heart, liver, and skeletal muscle, respectively (**Response Figure 7**). Despite the higher expression of vector DNA copies in the liver, this did not result in equivalent transcriptional output. Specifically, human *ALPK3* transcripts were robustly detected in the heart (3.6×10^7 copies per μg of RNA), but were only detected in one liver and skeletal muscle sample and at lower levels than cardiac tissue (2.6×10^6, and 8.6×10^5 transgene copies per μg of RNA, respectively, **Response Figure 7**). These data are consistent with cardiac-restricted transgene expression, likely mediated by the cardiac specific *TNNT2* promoter and inclusion of miR-122a-5p target sites to suppress hepatic expression. Importantly, these data establish a clear relationship between cardiac transgene expression and functional rescue, supporting the conclusion that the improvements in cardiac function arise from efficient *ALPK3* transduction.

With respect to safety, we have performed histological and biochemical assessment of off-target toxicity. Haematoxylin and eosin staining of liver and spleen from WT, *Alpk3*$^{KI/KI}$ AAV9-Control, and *Alpk3*$^{KI/KI}$ AAV9-TNNT2-*ALPK3* revealed no evidence of histopathology in either neonatal or adult cohorts (**Response Figure 12a, b;** *in manuscript* **Extended Data Figure 6g,h**). These findings were supported by organ weight (**Response Figure 12c, d;** *in manuscript* **Extended Data Figure 6c,d**) and serum analysis of the liver injury markers alanine aminotransferase (ALT) and Aspartate aminotransferase (AST), which showed no difference between groups (**Response Figure 12e, f;** *in manuscript* **Extended Data Figure 6e,f**). Thus, we have provided a more comprehensive characterisation of vector biodistribution, transgene expression, and short-term safety of this viral vector in the revised manuscript.

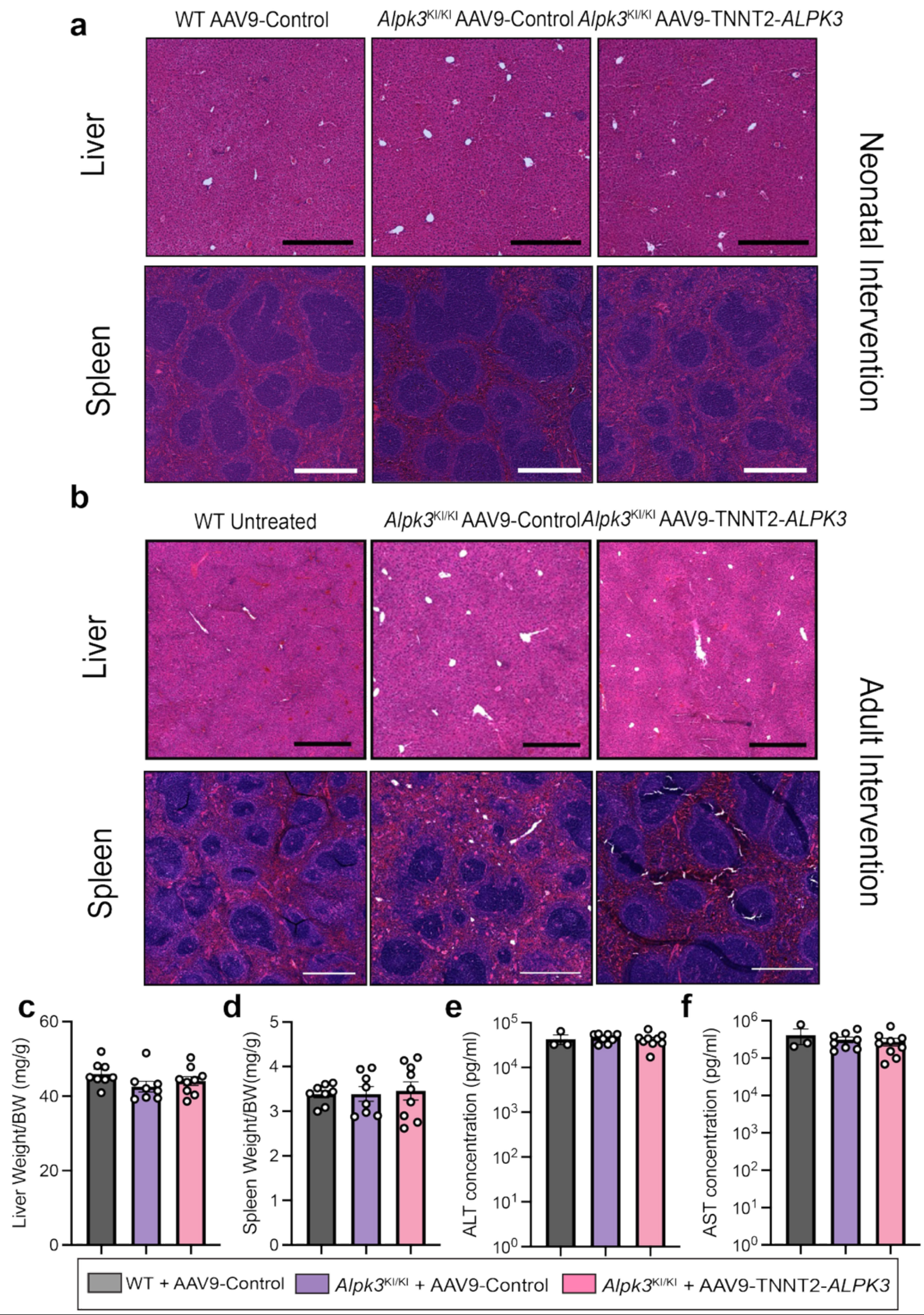

Response Figure 12. No evidence of off-target organ toxicity due to ALPK3 gene therapy. No detectable histopathology was observed in the livers or spleens of neonatal (**a**) or adult (**b**) intervention studies. **c. and d.** No changes in gross liver or spleen weight in adult intervention study. **e.** ALT and **f.** AST serum concentration across groups.

4. Adult disease "reversal" requires additional endpoints. While echocardiographic normalization after adult dosing is compelling, claims of disease reversal should be supported by histological analysis (fibrosis, cardiomyocyte size, sarcomeric organization), arrhythmia assessment, and/or potentially also longer-term durability of rescue.

We have extended our adult treatment study to include a longer-term follow-up to 18 weeks of age**.** Echocardiographic assessment demonstrated sustained rescue of cardiac function**,** including increased fractional shortening, reduced diastolic left ventricular posterior wall thickness, and improved diastolic function, as evidenced by an increased E/A ratio and reduced isovolumic relaxation time (**Response Figure 13a;** *in manuscript* **Figure 2d**).

Consistent with these functional improvements, heart weight to body weight ratio in *Alpk3*$^{KI/KI}$ mice treated with AAV9-TNNT2-ALPK3 was restored to levels comparable to wild-type controls (**Response Figure 13b;** *in manuscript* **Figure 2c**), indicating normalisation of gross cardiac remodelling.

At the molecular level, we observed reversal of key cardiac stress markers, with *Myh7, Nppa,* and *Nppb* reduced to near wild-type levels following treatment (**Response Figure 13c–e;** *in manuscript* **Figure 3b–d**). In addition, global profiling demonstrated an approximate 95% normalisation of the proteomic disease signature and 98% normalisation of the phosphoproteomic signature (**Response Figure 4**), supporting broad molecular remodelling toward a healthy state.

Histological analyses further confirmed structural rescue. Haematoxylin and eosin staining of four-chamber sections demonstrated restoration of overall cardiac architecture (**Response Figure 13f;** *in manuscript* **Figure 2e**). Moreover, wheat germ agglutinin staining revealed that cardiomyocyte cross-sectional area was increased approximately 50% in *Alpk3*$^{KI/KI}$ + AAV9-Control hearts compared with wild-type. This hypertrophy was fully normalised following AAV9-TNNT2-ALPK3 treatment (**Response Figure 13g;** *in manuscript* **Figure 2f**). Notably, cardiomyocyte hypertrophy is evident from early postnatal stages in this model (**Response Figure 16;** *in manuscript* **Extended Data Figure 1a-d**), indicating that treatment reverses an established phenotype.

Although arrhythmia assessment was not performed in the current study, the combination of sustained functional recovery, reversal of structural remodelling, and normalisation of molecular signatures over extended follow-up provides strong evidence supporting disease reversal in this model. We agree that additional longer-term durability studies (e.g. beyond 12 months) would be valuable to further assess the persistence of therapeutic benefit. However, given the breadth and consistency of the phenotypic, structural, and molecular rescue demonstrated here, we believe these studies extend beyond the scope of the current manuscript and represent an important direction for future investigation.

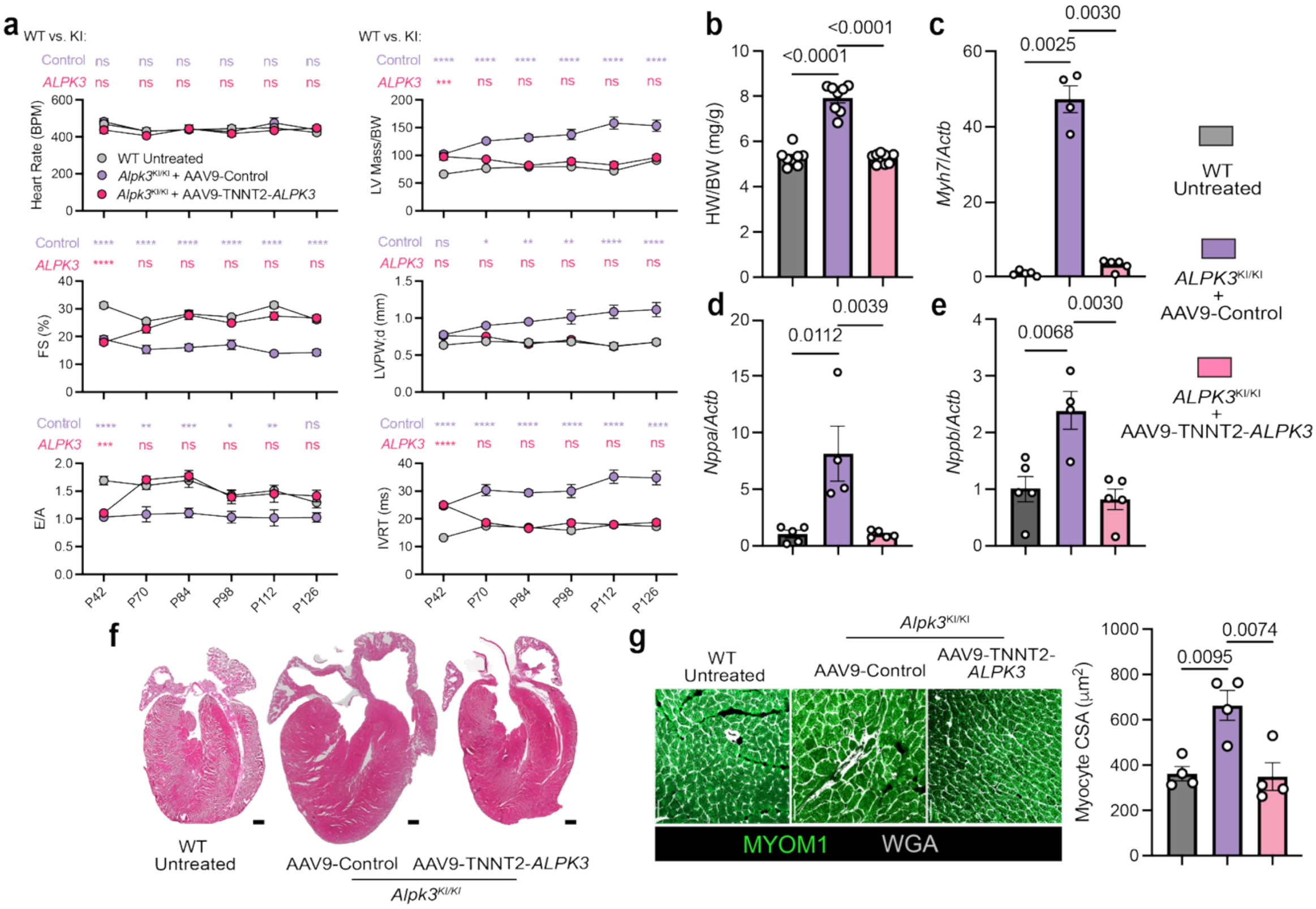

Response Figure 13. In-depth analysis of adult disease reversal following ALPK3 gene therapy. **a.** Extension of echocardiography analysis to P126 (12 weeks of treatment) confirms rescue of *Alpk3*$^{KI/KI}$ mice following gene therapy. **b.** End-point heart weight to body weight ratio. **c.** *Myh7,* **d.** *Nppa*, **e.** *Nppb* gene expression from RT-qPCR performed on left ventricular tissue. **f.** Haematoxylin and Eosin heart sections per group. **g.** Assessment of cardiomyocyte hypertrophy by wheat germ agglutinin staining.

5. Human ALPK3 models require clearer definition and controls. The precise ALPK3 variant(s), zygosity, and baseline phenotypes of the human cardiomyocytes/organoids should be explicitly stated. In addition, the observed increase in force with AAV-control treatment (Fig. 3c) require a control with WT organoids with vs. without vector treatment as well as a kinase-dead ALPK3 control.

We agree with the reviewer that additional detail would strengthen the description of the human cardiac organoid models. These details were abbreviated in the original submission due to word limits associated with the Letter format, but have now been fully incorporated into the Methods section, including specification of ALPK3 variants, zygosity, and baseline phenotypes.

The reviewer raises an important point regarding the observed increase in baseline force following AAV-control treatment (**Figure 4c**). To address this, we have now included additional data comparing wild-type organoids under untreated, AAV6-control, and AAV6-ALPK3 conditions. These analyses show that while AAV treatment itself results in a modest increase in contractile force, overexpression of ALPK3 does not further augment force generation in wild-type organoids (**Response Figure 14a;** *in manuscript* **Extended Data Figure 8a**). In addition, no differences were observed in beating rate or contraction and relaxation kinetics across these conditions (**Response Figure 14b–d;** *in manuscript* **Extended Data Figure 8b-d**).

These data indicate that the increase in force associated with AAV delivery is a vector-related effect, whereas the enhanced functional response observed with AAV6-ALPK3 in *ALPK3*^{mut}mutant and *TTN*$^{tv/+}$ organoids is genotype-specific. This interpretation is further supported by the minimal functional impact of AAV9-TNNT2-ALPK3 observed in wild-type mice (**Response Figure 9;** *in manuscript* **Extended Data Figure 4a-h)**.

With respect to the inclusion of a kinase-dead ALPK3, as discussed in response to reviewer 1, the precise kinase activity of ALPK3 has yet to be resolved (PMIDs: 39196058, 36321451, 38048395). We agree that inclusion of a kinase-dead ALPK3 construct could provide additional mechanistic insight, for example in distinguishing scaffolding versus catalytic functions. Notably, prior studies have shown that mice harbouring a kinase-dead ALPK3 mutation do not exhibit a cardiac phenotype (Feng et al., *Circulation*, 2023), supporting the notion that the therapeutic effects observed here may be mediated through non-catalytic mechanisms. In that context, at present this experiment requires further dissection of ALPK3 molecular function which is beyond the scope of the current study.

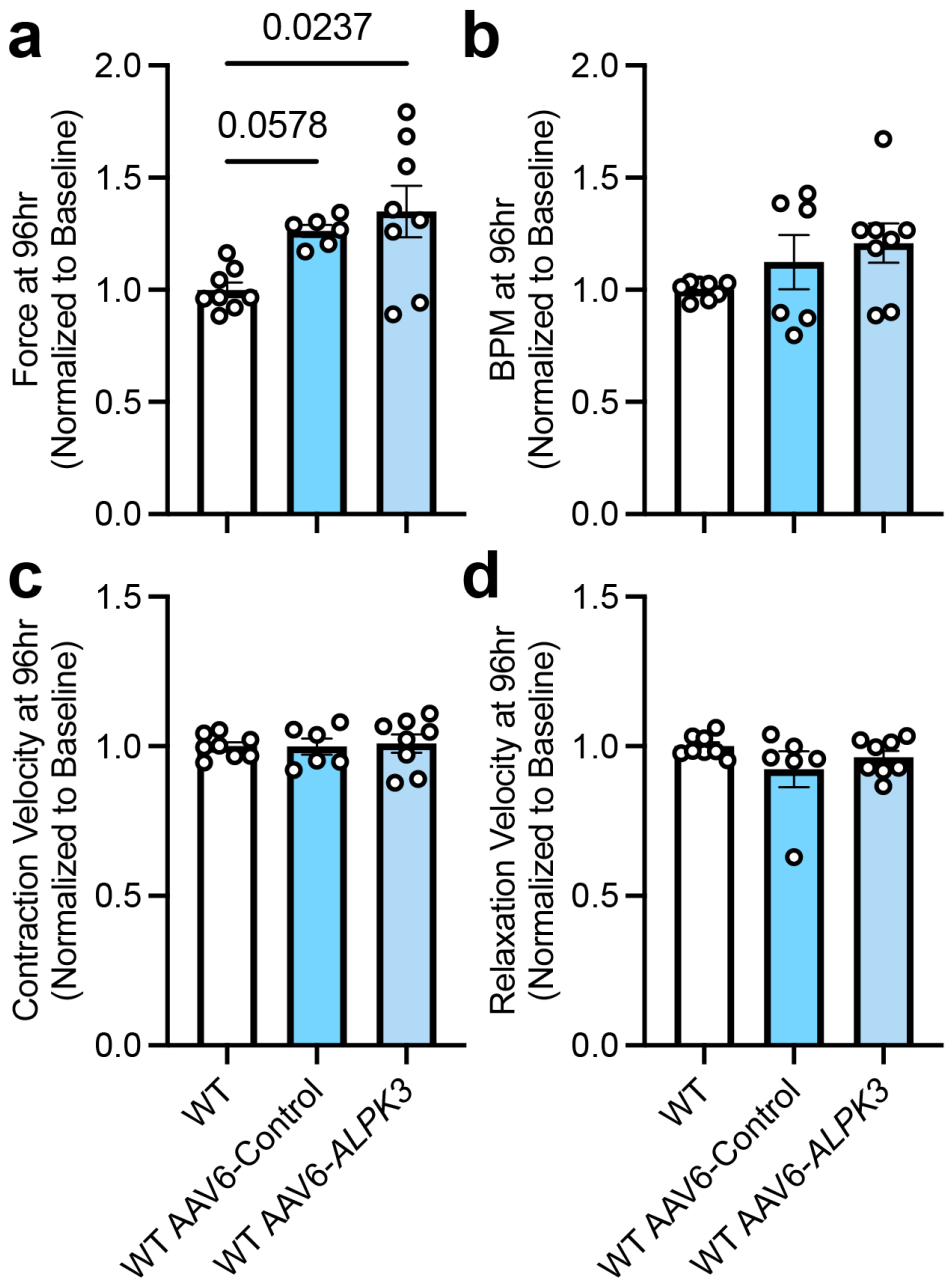

Response Figure 14. Minimal functional impact of ALPK3 gene therapy on wild-type cardiac organoids. **a.** Force at 96 hours of treatment normalised to baseline treatment. **b.** Baseline normalised beating rate, **c.** contraction velocity, and **d.** relaxation velocity.

6. The TTN cardiomyopathy extension is currently somewhat underpowered. The TTNtv data are limited to expression correlations and force rescue in organoids. Stronger mechanistic support is needed to justify broader claims, including direct readouts of proteostasis, sarcomere integrity etc.

We thank the reviewer for this important point. Previously, we had shown the raw force of each organoid data following 96 hours of treatment with AAV6-*ALPK3.* However, this approach did not consider the force of each organoid prior to treatment, and thus masked biological signal due to treatment. Following the reviewer's comment, we have reanalysed this organoid data to normalise endpoint force (96 hours) to baseline force, in line with our ALPK3 data and previously published approaches (PMIDs: 33811809, 37105170, 40562874). This demonstrated strong and consistent increases in force in *TTN*$^{tv/+}$ organoids that were specific to AAV6-*ALPK3* (**Response Figure 15a;** *in manuscript* **Figure 4i**). This normalisation approach is well-powered to detect statistically meaningful outcomes at 95% confidence and 90% power, and unmasks biologically meaningful impacts due to the intervention. No other contractile parameter was changed in *TTN*$^{tv/+}$ organoids treated with AAV6-*ALPK3* compared to AAV6-Control (**Response Figure 15b-d;** *in manuscript* **Extended Data Figure 8f-h**).

As outlined in **reviewer 2 response 2** (and associated **Response Figure 11**), we also performed proteomics to compare the signature of *TTN*$^{tv/+}$ organoids treated with either AAV6-*ALPK3* or AAV6-Control. This demonstrated dynamic regulation of proteins enriched for pathways related to proteasome function, proteolysis, cytoskeletal organisation, and ribonucleotide metabolism (**Response Figure 11g,h;** *in manuscript* **Figure 4j,k**), consistent with modulation of proteostasis networks. Here, we have additionally stained for ACTN2 in *TTN*$^{tv/+}$ monolayers, which showed disorganised sarcomeres in *TTN*$^{tv/+}$ compared to WT, which appeared rescued by treatment with AAV6-*ALPK3* (**Response Figure 15e;** *in manuscript* **Extended Data Figure 8i**). Together with the existing evidence for proteinopathy in the literature, altered E3 ligase localisation, and dysregulated ubiquitination, we believe this provides sufficient evidence and rationale for our claims of rescue via restored proteostasis.

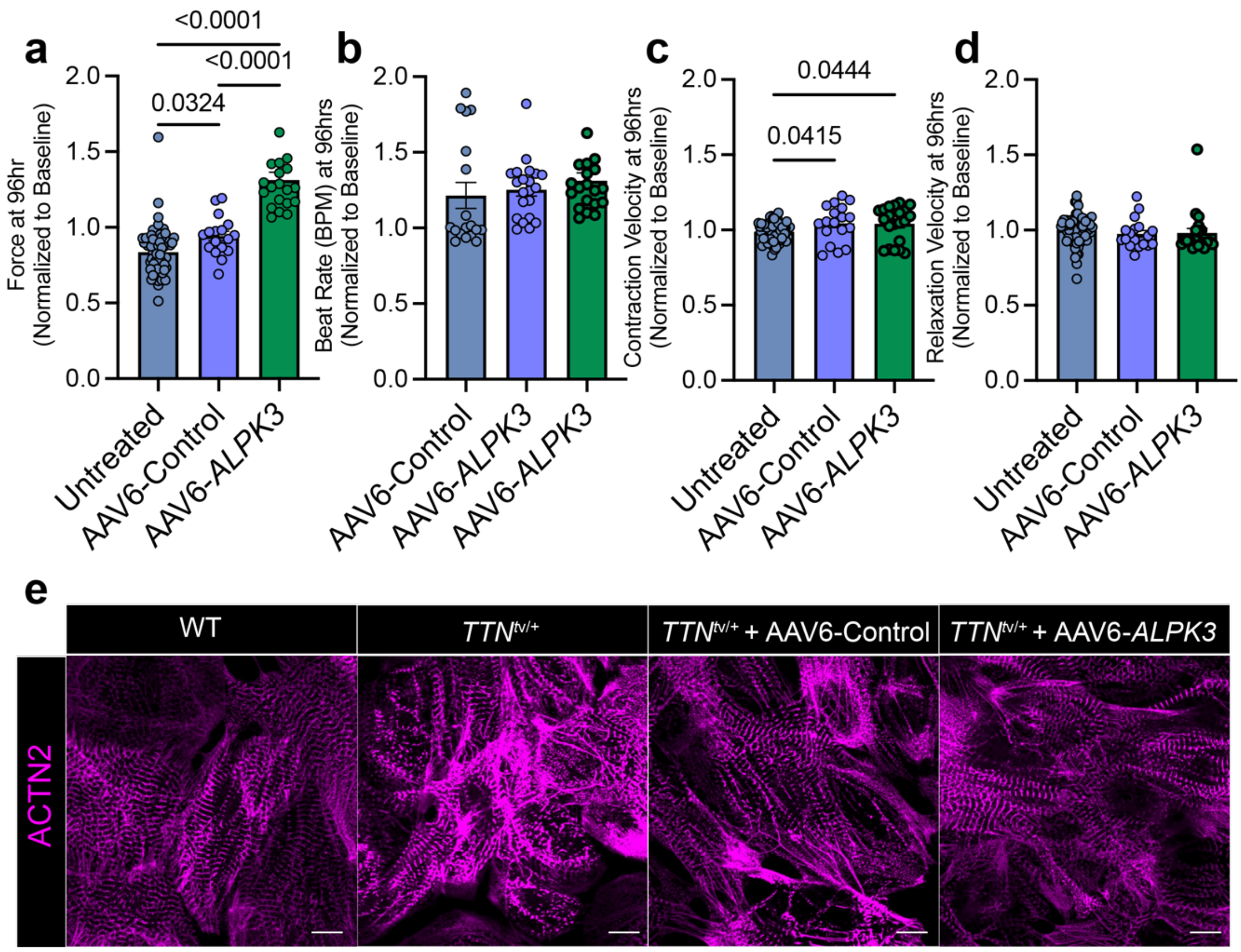

Response Figure 15. Improved analysis of TTN$^{tv/+}$ organoid data. **a,** Force at 96 hours normalised to baseline measurements reduces variability and power to detect significant differences. **b.-d.** No differences were observed in other measures of organoid function. **e.** Representative ACTN2 staining of *TTN$^{tv/+}$* and its isogenic control demonstrating restoration of proteinopathy in *TTN$^{tv/+}$* following *ALPK3* gene therapy.

MINOR COMMENTS

7. Model-to-patient alignment should be discussed more carefully.

The use of a homozygous truncation mouse model with early and severe disease contrasts with the typically dominant, later-onset human ALPK3 cardiomyopathy. The authors should discuss this discrepancy explicitly and clarify how well the current models reflect the intended clinical population.

This is an important consideration raised by the reviewer. It is true that homozygous *Alpk3* mice present with severe and early disease that mimics this rare patient population. These are not likely to be the main target population for this therapy but may qualify as a compassionate use case.

The more feasible target population would be the autosomal dominant *ALPK3* patients with an adult-onset cardiomyopathy. These patients account for between 1-2% of the hypertrophic cardiomyopathy population. The heterozygous *Alpk3*^{W1538X} mouse would therefore be the ideal model to study the impact of this gene therapy.

In a separate study that is in preparation, we have investigated phenotype of heterozygous *Alpk3* mice. As we previously reported (PMID 39196058), young heterozygous mice exhibit no cardiac phenotype. Indeed, in this study, we needed to age mice past 12 months to unearth a classical hypertrophic cardiomyopathy phenotype of preserved ejection fraction, ventricular wall thickening, and diastolic dysfunction. Clearly this is not a model that is readily amenable to therapeutic testing in the academic setting, but would be a focus of future development. These observations are quite similar to the *Mybpc3* mouse model which has been the focus of a gene therapy development program with the aim to restore haploinsufficiency. The *Mybpc3* heterozygous mouse similar develops cardiomyopathy after 12 months of age, so preclinical studies have focused on phenotype reversal in younger homozygous mice (PMIDs: 40038304, 25463264). Regardless, this AAV9 gene therapy developed by Tenaya Therapeutics has reached Phase 1b/2 clinical trials – MyPEAK-1 – with a focus on safety and efficacy in the autosomal dominant patient population (PMID: 41206746). There is clearly well-established rationale for using these homozygous models in preclinical studies.

We have added discussion to this point as follows: "These studies utilised homozygous *Alpk3*^{W1538X} mice, which model the rare and severe autosomal recessive disease rather than the more common autosomal dominant patients, which account for ~2% of all HCM. We, and others, have not observed phenotypes in young heterozygous *Alpk3*^{W1538X} mice. Indeed, HCM is only detectable in these mice after 12 months of age (data not shown). While this models the patient trajectory well, it not readily amenable to therapeutic testing. In this context, *ALPK3* gene therapy development is analogous to the development of *MYBPC3* gene therapies, in which Phase 1b/2 clinical trials (MyPEAK-1)[26] to assess safety and efficacy are focussed on the autosomal dominant patient population, despite pre-clinical work using more severe homozygous knockout mice.[27] Thus, the use of homozygous animal models in preclinical studies to provide rapid proof-of-concept support for therapeutic efficacy is an established paradigm for myotropic gene therapies.[3,5]"

8. The authors should consider deposition of key datasets rather than data availability upon request.

We agree with the reviewer on this point. We have deposited key datasets to online repositories. Single nucleus RNA sequencing has been deposited to Gene Expression Omnibus (GSE328808; reviewer token gfudsoiknrulxqd). Additionally, all proteomics datasets have been deposited to the PRotein IDEntifications Database (PRIDE) (accession number PXD077620; reviewer token 3l4h7uhFAZil). These will be made publicly available upon publication of the manuscript.

Reviewer #3 (Remarks to the Author):

1. In page 3, 3rd paragraph, they stated “Homozygous mice are born with enlarged hearts”, but this is not described in the original paper reporting this mouse model. Please provide the evidence of neonatal cardiac enlargement.

The reviewer raises an important point regarding the early onset of disease in this model. We have now systematically assessed the cardiac phenotype in P0 mice, comparing wild-type to *Alpk3*$^{KI/KI}$ mice. We observed a significant increase in the heart weight to body ratio in *Alpk3*$^{KI/KI}$ mice (6.3 ± 0.2 mg/g vs. 5.2 ± 0.2mg/g, p < 0.0001; **Repsonse Figure 16a;** *in manuscript* **Extended Data Figure 1a**), and clear organ hypertrophy by H&E staining (**Repsonse Figure 16b;** *in manuscript* **Extended Data Figure 1b**). We stained the left ventricles of these mice with wheat gern agglutinin and cardiac troponin-T to assess whether this hypertrophy arose from cardiomyocyte growth (**Repsonse Figure 16c;** *in manuscript* **Extended Data Figure 1c**). Quantification of this imaging confirmed a significant increase in cardiomyocyte size (37 ± 2 μm^2 vs 34 ± 1μm^2, p = 0.0141; (**Repsonse Figure 16d;** *in manuscript* **Extended Data Figure 1d**). Collectively, these data confirm the statement that homozygous mice are born with enlarged hearts.

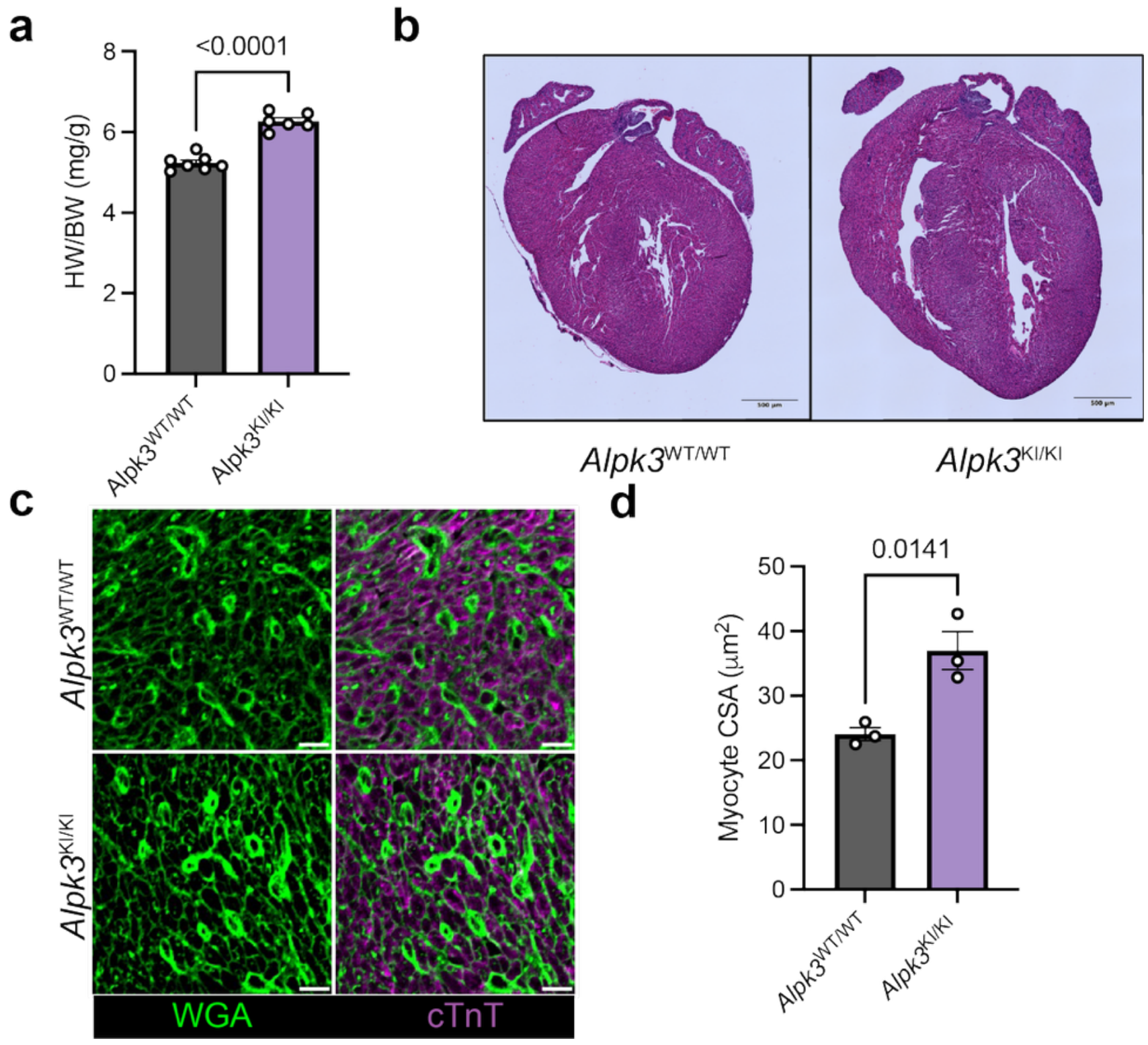

Response Figure 16. Neonatal onset of cardiomyopathy in P0 *Alpk3*$^{KI/KI}$ mice. a. Heart weight to body weight ratio of wild-type and homozygous P0 mice. **b.** Representative H&E image of the same mice demonstrating organ hypertrophy. **c.** Representative WGA (green) and cardiac troponin-T (purple) staining of P0 left ventricle tissue to assess myocyte hypertrophy, and **d.** quantification of cardiomyocyte cross sectional area (average of ~100 myocytes per mouse, 3 mice per group).

2. They use ejection fraction as a parameter of cardiac systolic function but since mouse heart rate is very fast, it is more appropriate to use fractional shortening avoiding potential error inflation.

Within Figures 1 and 2, we have replaced ejection fraction with fractional shortening. We note that this has not impacted the results or their interpretation.

3. Since this is a mouse model of HCM AND DCM, they should present LVDd in main figure.

We appreciate the reviewers' request to include LVIDd in the main figure. Having analysed all echocardiography parameters, LVID;d is one of the few to display little to no significant change, while LVID;s is indeed rescued back to healthy levels, thereby increasing ejection fraction and fractional shortening. Given the limited space in this Letter format, we have chosen to show just the most pertinent values, key to our findings. However, we now include two supplementary tables "Supplementary-Table-1_Neonatal-Echocardiography" and "Supplementary-Table-2_Adult-Echocardiography" which provide the full comprehensive echocardiography data for all timepoints in neonatal and adult studies.

4. They use IVRT as a parameter of diastolic function which I agree that it is quite reliable method, however, since IVRT is a duration that was affected strongly by HR, and again, mouse HR is very high and varies, I would recommend to use IVRT adjusted to heart rate (normalized IVRT).

As requested, we have replaced IVRT to a heart rate normalised measurement for both neonatal and adult intervention studies (**Response Figure 17a,b;** *in manuscript* **Figures 1g and 2d**). We note that this has not changed the interpretation of the data.

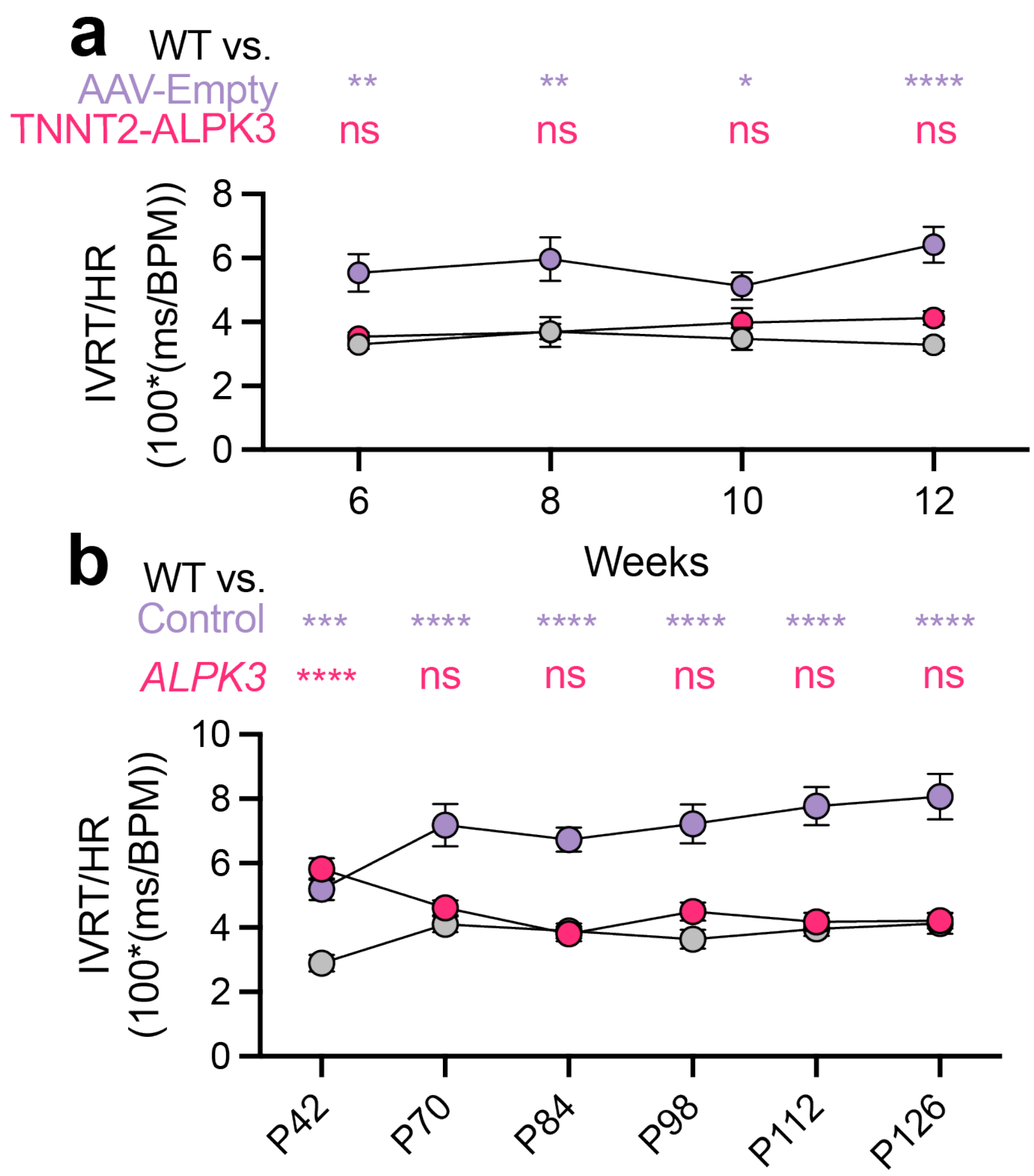

Response Figure 17. Heart rate normalisation of isovolumic relaxation time (IVRT) confirms diastolic dysfunction. Normalised IVRT in **a.** neonatal mice and **b.** adult mice show elongation of IVRT in *Alpk3*$^{KI/KI}$ mice which is normalised upon treatment with AAV9-TNNT2-*ALPK3*.

5. Extended data 1b was not appeared in the text body. Why wt is with AAV6 not AAV9?

We apologise for this oversight. In the resubmission, we have corrected these errors.

6. In page 4, 1st paragraph, they claim there are no sign of off-target organ pathologies. Since the virus titer was relatively high which is ~2x1014 vg/kg, and hepatotoxicity is one of the most common side effects, please confirm that there are no findings to suggest hepatotoxicity.

As also requested (and outlined in detail in response to) by **Reviewer 2, Point 3** (and associated **Response Figure 12**), we have performed qPCR for vector and transgene copy number, histology for structural changes, and ELISAs for the liver damage markers ALT and AST, no evidence of hepatotoxicity could be observed 12 weeks following treatment. For ease of viewing, **Response Figure 12** is presented again below.

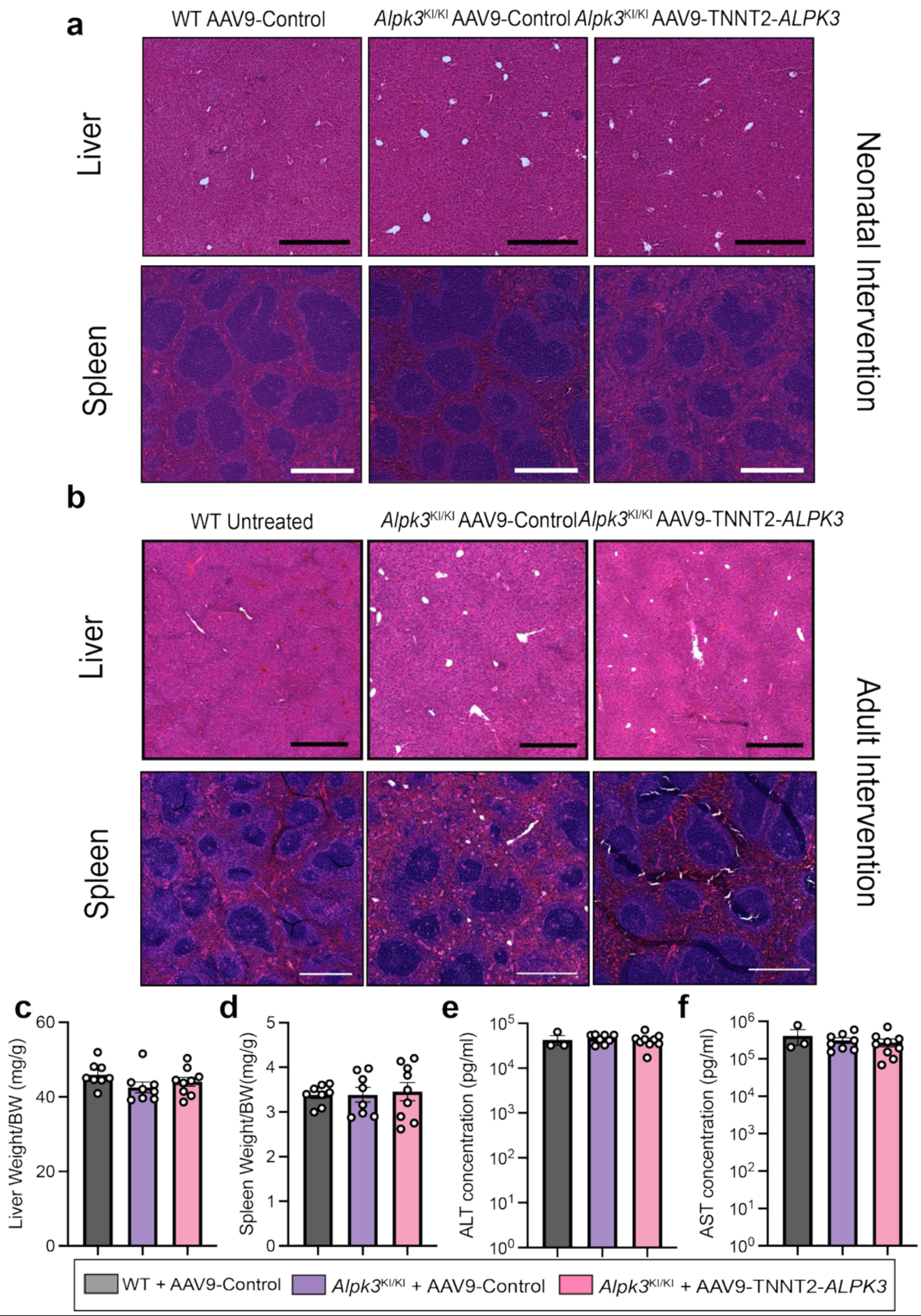

Response Figure 12. No evidence of off-target organ toxicity due to ALPK3 gene therapy. No detectable histopathology was observed in the livers or spleens of neonatal (**a**) or adult (**b**) intervention studies. **c. and d.** No changes in gross liver or spleen weight in adult intervention study. **e.** ALT and **f.** AST serum concentration across groups.

7. Reversed HCM AND DCM phenotype in adult mice by AAV9-Tnnt2-Alpk3 was determined based on echo data. LVPW reduced its thickness by approximately 0.2mm in only 2 weeks in adult treatment group which is a relatively acute change. Are there any histological changes, ie fibrosis status? Do they reverse fibrosis as well? Please provide histological assessment.

We agree with the reviewer that the reduction in left ventricular wall thickness was surprisingly acute, potentially suggesting the high sensitivity of the heart to ALPK3 expression. We have now provided histology (H&E). Please see response to Reviewer 2 (**Response Figure 13**). Interestingly, we have not observed any fibrosis in the *Alpk3*^{W1538X} model despite the substantial cardiac dysfunction.

8. They performed single nucRNA sequencing of TTNtv/tv, TTNtv/+ and TTN+/+ only to prove relative ALPK3 expression level alters by TTN gene dosage. They should provide more intensive analysis to examine ie other gene expression in the complex at M-band. The mechanism of how TTNtv variant cause decrement of ALPK3 expression is not clear. It would be beneficial to have mechanistic insights to understand how ALPK3 expression decreases and hence the ALPK3 supplement improve CM contractile function in TTNtv variant.

We agree with the reviewer that the observation of reduced *ALPK3* expression in *TTN*tv cardiac organoids is intriguing and warrants further mechanistic investigation. Elucidating the pathways linking TTN truncating variants to reduced ALPK3 expression would indeed provide valuable biological insight. However, we consider this to be a substantial line of investigation that extends beyond the scope of the current study. However, to further explore the relationship between ALPK3 and TTN, we applied Geneformer, a foundational AI model for network biology, to perform *in silico* genome-wide combinatorial gene perturbation, deleting each gene in combination with *ALPK3* and comparing the predicted impact to deleting *ALPK3* alone (See Response to Reviewer 2, point 2 and **Response Figure 11e**; *in manuscript* **Figure 4e)**. Notably, combined deletion of *ALPK3* and *TTN* produced the most pronounced predicted disruption in cardiomyocyte state, supporting the concept that these proteins function within a shared regulatory network.

The central finding of this manuscript is that restoration of sarcomeric proteostasis—an established pathogenic mechanism in TTN cardiomyopathy—via ALPK3 is sufficient to improve cardiac function. Importantly, this therapeutic effect does not require ALPK3 expression to be reduced per se, but rather highlights sarcomeric proteostasis as a tractable target of intervention.

To further address this point, we have now performed quantitative proteomic analyses of TTNtv/+ cardiac organoids treated with either AAV-ALPK3 or AAV-Control (**Response Fig. 11f–i; Fig. 4f–i**). These data demonstrate that ALPK3 gene therapy modulates key proteostasis pathways, including the proteasome and RNA metabolism, supporting a role for ALPK3 as a regulator of cardiac proteostasis in this context.

In addition, we examined expression of other components of the ALPK3–TTN-associated M-band complex using our single-nucleus RNA sequencing dataset (**Response Fig. 18**). In line with the reviewer's suggestion, we observed that, alongside gene dosage-dependent effects on *TTN* and *ALPK3*, several M-band-associated genes, including *MYH7*, *TRIM55*, *MYOM1*, and *MYOM2*, are dysregulated in TTNtv organoids. While these findings support broader disruption

of the M-band proteostasis network in response to *TTNtv*, the process by which sarcomeric dysfunction is interpreted by the cardiomyocyte to elicit a transcriptional response remains poorly understood.

Given the descriptive nature of these observations and the absence of a defined mechanistic pathway, we have chosen not to include these data in the main manuscript. However, we believe they further support our overall conclusion that TTN truncating variants disrupt a coordinated sarcomeric proteostasis network that can be functionally restored by ALPK3 gene therapy. Future studies will be directed toward defining the molecular mechanisms linking TTN dysfunction to ALPK3 regulation.

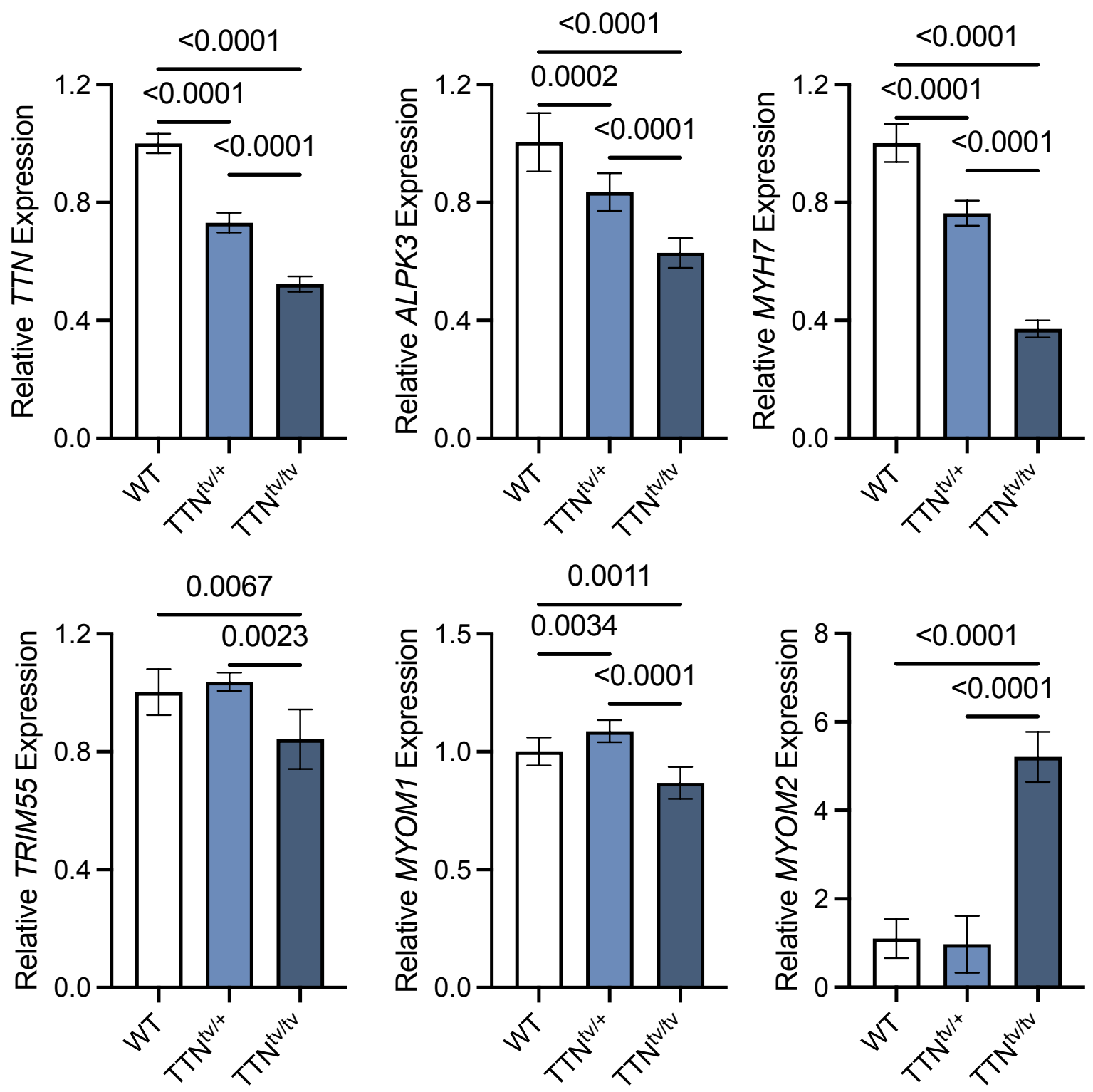

Response Figure 18. Dysregulation of the ALPK3-TTN complex at the M-band. *Relative expression of a selection of genes encoding M-Band proteins, quantified from snRNAseq data (n=8 per group).*

9.They should describe more detailed methods such as echocardiography or functional assessment of organoid. How they identify arrhythmogenicity of organoid? Even though they have used the same protocol as previously described elsewhere, brief description should be provided.

As noted above, some technical details that have been previously published were not included as this publication was submitted as a brief Letter format. To address the reviewer's concern, we have included greater detail of echocardiography and organoid function in the revised manuscript.

10. There is a typo in mouse description in method section. C57/BL6J should be C57BL/6J.

Thank you for capturing this typographical error. It has been corrected

We would like to thank the reviewers for their time and consideration of our revised manuscript. We appreciate the overall positive response to our revision. Below, we provide responses to the minor concerns raised by Reviewers 2 and 3. We hope that by addressing these concerns, the manuscript will now be considered suitable for publication in *Nature Cardiovascular Research*. Relevant changes within the manuscript have been highlighted in **blue text.**

Reviewer #2 (Remarks to the Author):

1.The claim that the mini-version lacks "functionally important domains" rests on a cross-study comparison with different capsids and routes. I would soften that wording in the discussion.

We appreciate the reviewer's concern on this point and agree that without direct comparisons between the miniALPK3 and full-length ALPK3 viral constructs in the same mouse model, viral capsid, and delivery route, it is difficult to ascertain whether the miniALPK3 protein indeed lacks functionality of the full-length protein. However, we do believe this is an important consideration for any miniaturised gene therapy. Therefore, we have softened the wording as follows: "While this is based on a cross-study comparison between two independent studies that use different mouse models, viral capsids, and delivery routes, these findings raise the possibility that the miniALPK3 construct may not fully recapitulate the activity of full-length ALPK3. Further studies would be required to systematically assess the functionality of the intrinsically disordered region deleted in the miniALPK3 construct."

2.Could the authors potentially provide any kind of quantification for the sarcomere claim in Fig. 15e?

We apologise for the confusion, but our manuscript does not have a Fig. 15e. We believe the reviewer must be referring to Extended data figure **8e**, where we claim that ALPK3 correctly localises to the sarcomere in a wild-type setting. Here we provide a reviewer only figure demonstrating a line profile of fluorescent intensity that clearly shows ALPK3 interdigitating with ACTN2, demonstrating correct localisation to the sarcomeric M-Band (**Response figure 1).** For any other sarcomeric quantification, since we are now unblinded to the study, we risk producing inadvertent bias into the study and would be hesitant to quantify. The sarcomeric disarray reported in our study aligns with other reports (PMIDs: 21441111, 36321451, 39196058), and the substantial phenotypic and molecular reversal agrees with the histological finding of restoration of sarcomeric structure.

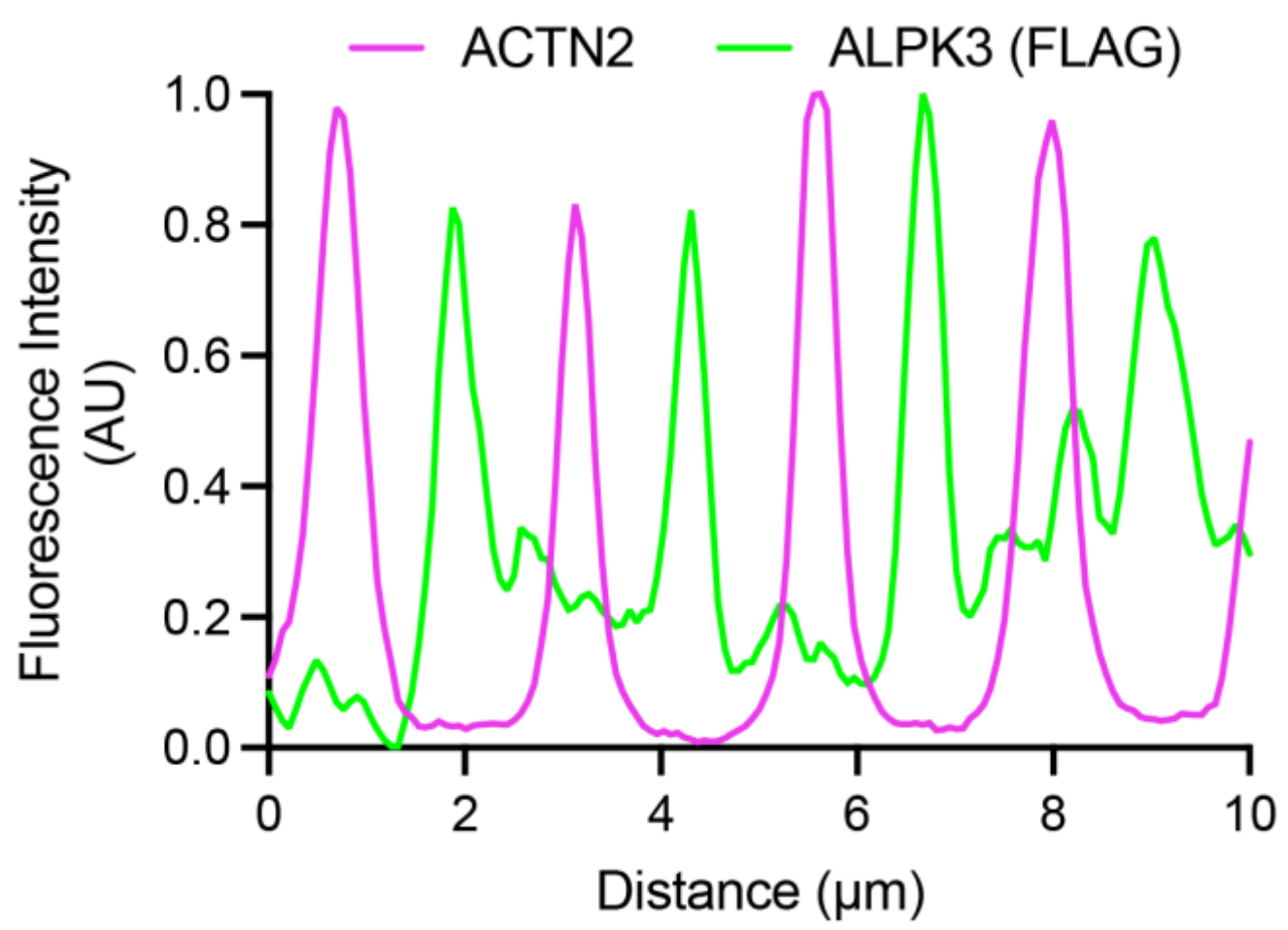

Response figure 1. Correct localisation of ALPK3 in WT cardiomyocytes. Representative line profile pertaining to **extended data figure 8e** shows interdigitation of ALPK3-FLAG and ACTN2 confirming the correct localisation to the cardiac M-Band.

Reviewer #3 (Remarks to the Author):

I have three minor concerns.

#1, The quantification of cell size were presented in Fig. 2f, Extended Data Fig. 1c, d. It is not clearly stated how they collected the data. Since the cardiomyocyte area measurement depends on the cell alignment, ie cross section over longitudinal, they should collect multiple different layers from each heart, from basal to the apex. What is their definition of cross section?

Cardiomyocyte cross sectional area was assessed using wheat germ agglutinin to label the cell walls and extracellular matrix in the heart. Multiple images from the left ventricle were collected from apex to base in sagittal sections. Cell area was assessed using ImageJ software in cardiomyocytes with a clear cross-sectional orientation (i.e. cells with a relatively uniform round shape). Cardiomyocytes oriented longitudinally or obliquely were not considered for cross-sectional area measurements. This approach has been used widely in the field (e.g. PMIDs: 32817558, 21111744, 30586742, 36351918). Approximately 100 myocytes per mouse were analysed. We have added methodology to the paper and hope this clarifies the issue.

#2, Do the authors have any thoughts why they have not observed any fibrosis in the Alpk3W1538X model? ALPK3 cardiomyopathy is known to have extensive fibrosis. They have performed WGA staining, but how about Masson's trichrome or Sirius Red staining? Did snRNAseq data present any changes in collagen or other fibrosis related genes? Fibrosis reduction/regression should be one of the key components in terms of the ALPK3 cardiomyopathy treatment.

The reviewer raises an interesting question. We have also been surprised by the absence of appreciable fibrosis in such a severe model of cardiomyopathy. The reasons behind this remain unclear, but we note that this is consistent with data published by Van Sligtenhorst et al. (PMID: 21441111), who noted an absence of interstitial fibrosis in the hearts of *Alpk3*$^{-/-}$ mice that is classically observed with pathological cardiac hypertrophy. Feng et al. (PMID: 41248220) did observe a mild increase in the mRNA expression of *Col1a1* and *Col3a1* but did not report on other indices of fibrosis. Similarly, Agarwal et al. (PMID: 36321451) did not report fibrosis indices. One speculative reason for the absence of fibrosis is mouse strain. Both our *Alpk3*^{W1538X} and the Van Sligtenhorst *Alpk3*$^{-/-}$ mouse models were maintained on the C57BL/6J substrain, while the Feng *Alpk3*$^{-/-}$ mouse model is only reported as a C57BL/6 strain, but appears to be a mixture of 6J and 6N substrains. The C57BL/6J strain has been demonstrated to be more resistant to fibrosis in response to pathological stimuli (e.g. angiotensin II, pressure overload) than its C57BL/6N counterpart (PMIDs: 23709599, 25069667, 26256392).

We have compiled RNA sequencing data that further demonstrates an absence of appreciable fibrotic remodelling in the *Alpk3*^{W1538X} mouse model, assessed by *Col1a1*, *Col3a1*, and *Postn* expression (**Response figure 2a-c**). Further, we have analysed collagen and periostin abundance from an unpublished proteomics dataset that assesses the temporal disease signature of *Alpk3*^{W1538X} hearts between 3 and 18 months of age (**Response figure 2d**). This demonstrated an age-dependent onset of a fibrotic signature in homozygous *Alpk3* mice very late in the disease process (18 months of age).

We have added text to page 10 of the main text to acknowledge this limitation of the Alpk3 mouse in modelling the fibrosis observed in ALPK3 cardiomyopathy patients: "Given the

Alpk3^{W1538X} model does not develop substantial interstitial fibrosis despite the severity of disease, future studies will be required to determine if this level of functional rescue is still observed in hearts with pre-existing fibrosis."

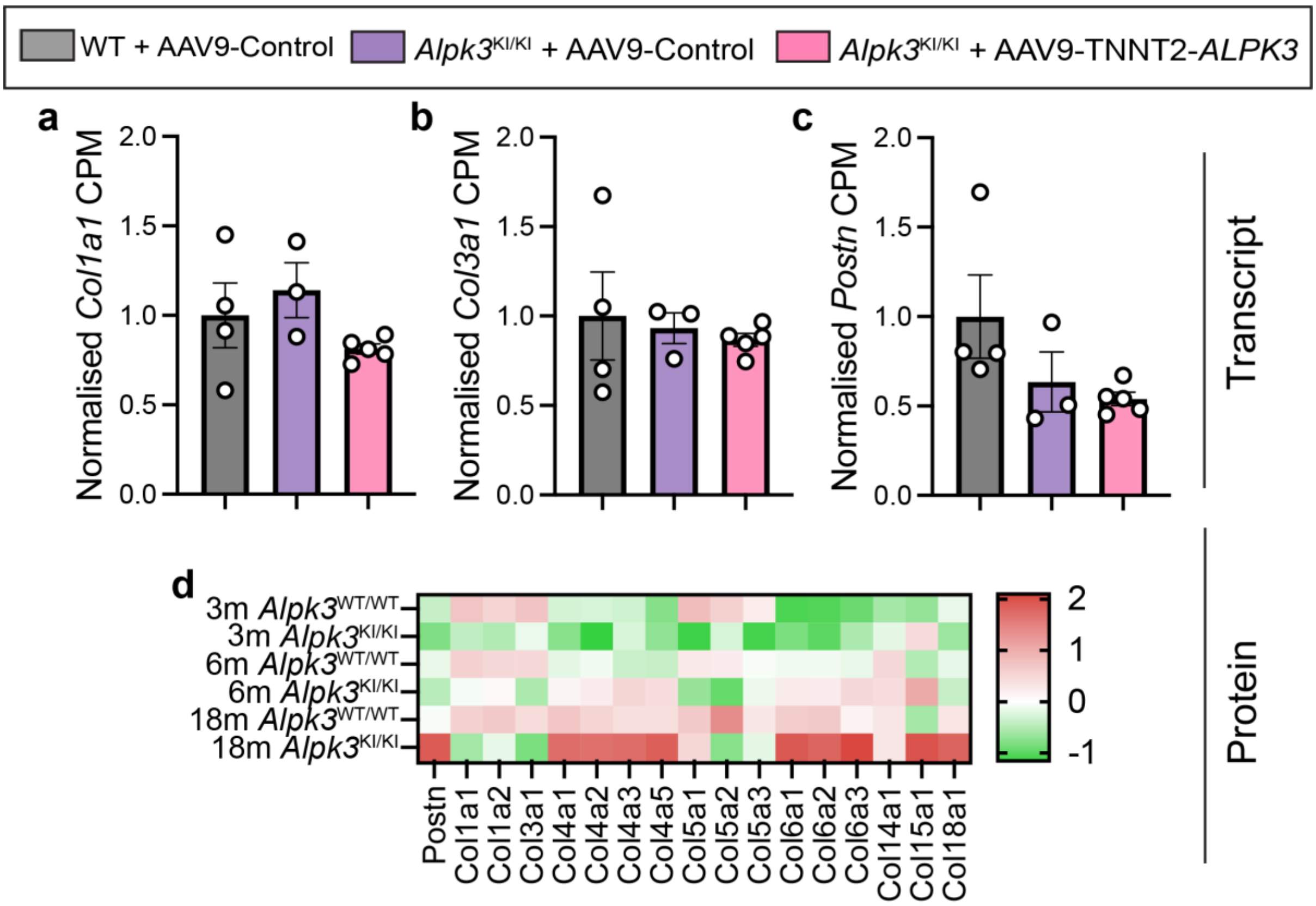

Response figure 1. Absence of fibrosis in *Alpk3*^{W1538X} mouse hearts. a, *Col1a1*, **b,** *Col3a1*, and **c,** *Postn* gene expression between WT mice and *Alpk3*^{W1538X} mice treated with either AAV9-Control or AAV9-TNNT2-*ALPK3* demonstrates no appreciable differences in classical fibrosis markers. **d,** heatmap of proteomics data at ages 3-, 6-, and 18-months of age show no appreciable increase in periostin and collagen abundance until 18-months of age.

#3, In echocardiography method, please clarify at what HR they collected the data. Cardiac function is altered by HR. Bradycardia causes reduced FS even in WT mice. They have changed EF to FS in the figures, but there are several sentences still stating ejection fraction in main body.

Echocardiography data was collected with an average heart rate of 470bpm and 440bpm for neonatal and adult studies, respectively. No significant differences were observed between groups at any timepoint, suggesting heart rate does not impact echocardiography measurements between groups. This heart rate data is readily available in Figure 2d (adult study), "Supplementary-Table-1_Neonatal-Echocardiography", and "Supplementary-Table-2_Adult-Echocardiography". We apologise for the remaining references to ejection fraction and have now replaced these with fractional shortening, where appropriate.
